# The Extent and Impact of Variation in ADME Genes in Sub-Saharan African Populations

**DOI:** 10.3389/fphar.2021.634016

**Published:** 2021-04-28

**Authors:** Jorge E. B. da Rocha, Houcemeddine Othman, Gerrit Botha, Laura Cottino, David Twesigomwe, Samah Ahmed, Britt I. Drögemöller, Faisal M. Fadlelmola, Philip Machanick, Mamana Mbiyavanga, Sumir Panji, Galen E. B. Wright, Clement Adebamowo, Mogomotsi Matshaba, Michéle Ramsay, Gustave Simo, Martin C. Simuunza, Caroline T. Tiemessen, Sandra Baldwin, Mathias Chiano, Charles Cox, Annette S. Gross, Pamela Thomas, Francisco-Javier Gamo, Scott Hazelhurst

**Affiliations:** ^1^ Sydney Brenner Institute for Molecular Bioscience (SBIMB), Faculty of Health Sciences, University of the Witwatersrand, Johannesburg, South Africa; ^2^ Division of Human Genetics, National Health Laboratory Service and School of Pathology, Faculty of Health Sciences, University of the Witwatersrand, Johannesburg, South Africa; ^3^ Computational Biology Division and H3ABioNet, Department of Integrative Biomedical Sciences, University of Cape Town, Cape Town, South Africa; ^4^ Centre for Bioinformatics and Systems Biology, Faculty of Science, University of Khartoum, Khartoum, Sudan; ^5^ Department of Biochemistry and Medical Genetics, University of Manitoba, Winnipeg, MB, Canada; ^6^ Department of Computer Science, Rhodes University, Makhanda, South Africa; ^7^ Neuroscience Research Program, Winnipeg Health Sciences Centre and Max Rady College of Medicine, Kleysen for Advanced Medicine, University of Manitoba, Winnipeg, MB, Canada; ^8^ Department of Pharmacology and Therapeutics, Rady Faculty of Health Sciences, University of Manitoba, Winnipeg, MB, Canada; ^9^ Institute for Human Virology, Abuja, Nigeria; ^10^ Institute of Human Virology and Greenebaum Comprehensive Cancer Center, University of Maryland School of Medicine, Baltimore, MD, United States; ^11^ Botswana-Baylor Children’s Clinical Center of Excellence, Gaborone, Botswana; ^12^ Baylor College of Medicine, Houston, TX, United States; ^13^ Molecular Parasitology and Entomology Unit, Department of Biochemistry, Faculty of Science, University of Dschang, Dschang, Cameroon; ^14^ Department of Disease Control, School of Veterinary Medicine, University of Zambia, Lusaka, Zambia; ^15^ Centre for HIV and STIs, National Institute for Communicable Diseases, National Health Laboratory Services and Faculty of Health Sciences, University of the Witwatersrand, Johannesburg, South Africa; ^16^ Drug Metabolism and Pharmacokinetics, GlaxoSmithKline R&D, Ware, United Kingdom; ^17^ Human Genetics, GlaxoSmithKline R&D, Stevenage, United Kingdom; ^18^ Clinical Pharmacology Modelling and Simulation, GlaxoSmithKline R&D, Sydney, NSW, Australia; ^19^ Data and Computational Sciences, GlaxoSmithKline R&D, Stevenage, United Kingdom; ^20^ Global Health, GlaxoSmithKline R&D, Tres Cantos, Madrid, Spain; ^21^ School of Electrical and Information Engineering, University of the Witwatersrand, Johannesburg, South Africa

**Keywords:** ADME, genetic diversity, Africa, pharmacogenomics, CNV

## Abstract

**Introduction:** Investigating variation in genes involved in the absorption, distribution, metabolism, and excretion (ADME) of drugs are key to characterizing pharmacogenomic (PGx) relationships. ADME gene variation is relatively well characterized in European and Asian populations, but data from African populations are under-studied—which has implications for drug safety and effective use in Africa.

**Results:** We identified significant ADME gene variation in African populations using data from 458 high-coverage whole genome sequences, 412 of which are novel, and from previously available African sequences from the 1,000 Genomes Project. ADME variation was not uniform across African populations, particularly within high impact coding variation. Copy number variation was detected in 116 ADME genes, with equal ratios of duplications/deletions. We identified 930 potential high impact coding variants, of which most are discrete to a single African population cluster. Large frequency differences (i.e., >10%) were seen in common high impact variants between clusters. Several novel variants are predicted to have a significant impact on protein structure, but additional functional work is needed to confirm the outcome of these for PGx use. Most variants of known clinical outcome are rare in Africa compared to European populations, potentially reflecting a clinical PGx research bias to European populations.

**Discussion:** The genetic diversity of ADME genes across sub-Saharan African populations is large. The Southern African population cluster is most distinct from that of far West Africa. PGx strategies based on European variants will be of limited use in African populations. Although established variants are important, PGx must take into account the full range of African variation. This work urges further characterization of variants in African populations including *in vitro* and *in silico* studies, and to consider the unique African ADME landscape when developing precision medicine guidelines and tools for African populations.

## 1 Introduction and Background

Pharmacogenomics (PGx) aims to improve drug safety and efficacy using genomic knowledge for genes involved in drug action (Roden et al., 2019) with a focus on genes that have important roles in drug safety, pharmacokinetics and pharmacodynamics. Genes involved in pharmacokinetics are typically defined by the role they play in the absorption, distribution, metabolism and excretion (ADME) of drug molecules.

Variation in ADME genes play an important role in determining the response to drug treatment in an individual patient. We characterize the extent and impact of variation in these genes in a novel, high-coverage whole genome sequence dataset from a diverse group of Africans.

ADME genes have different functions: 1) phase I metabolizing enzymes, 2) phase II metabolizing enzymes, 3) drug transporters and 4) modifiers. PharmaADME (http://pharmaadme.org) classifies the ADME genes in two classes. The 32 *core* genes have known biomarkers linked to ADME outcomes. For the 267 *extended* ADME genes, there is weaker evidence of functional consequences *in vitro* or *in vivo*, or they are important for a limited number of drugs only.

### 1.1 Rationale

Currently the majority of patients studied in drug development programmes are of European or Asian ancestry. The African continent is the cradle of human origin and African populations are characterized by high genetic diversity and complex population structure. Despite this genetic variation, drug efficacy and safety have not been comprehensively studied in the populations of sub-Saharan Africa (SSA) (Radouani et al., 2020). This is of specific relevance to SSA, where high burdens of disease are amplified by non-optimal treatment outcomes.

The particular diversity of ADME genes in SSA has been reported in some studies. Hovelson et al. (Hovelson et al., 2017) and Lakiotaki et al. (Lakiotaki et al., 2017) found that the greatest levels of coding ADME variation per personal haplotype were shown in some African populations sampled in the 1,000 Genomes Project (KGP) data. Examples of the impact of this variation can be seen in *CYP2B6* and *CYP2D6* variation affecting efavirenz and primaquine respectively. An efavirenz dosage reduction has been recommended for HIV patients in SSA due to the high frequency of functional variants in the *CYP2B6* gene that result in a higher risk of adverse drug reactions (Mukonzo, 2014). Potential polymorphisms in the human cytochrome *CYP2D6* gene may negatively influence efficacy of primaquine, and significantly affect malaria elimination strategies (Dandara et al., 2014; Awandu et al., 2018). African specific variation in several genes may impact the pharmacokinetics of rosuvastatin, a drug used to treat hypercholesterolemia (Soko et al., 2018). While these studies represent only a fraction of the continent, they serve to highlight the importance of future studies which are aimed at providing a more comprehensive overview of the landscape of ADME variation across Africa.

Therefore it is important to gain a better understanding of the variation that exists in ADME genes, both within and between different SSA populations. This information could be used to inform recommended drug dosage regimens for patients in SSA based on potential pharmacokinetic effects and consequently efficacy and safety. To date, no studies have systematically investigated ADME variation within a diverse set of African populations. We therefore aim to provide valuable information regarding the variation that exists in ADME genes, both within and between different SSA populations. This information could provide insight into drug efficacy and safety for patients in SSA and play a role in ensuring safe and efficacious treatments for the high burden of diseases in populations in SSA.

## 2 Results

### 2.1 Description of Samples

**FIGURE 1:**
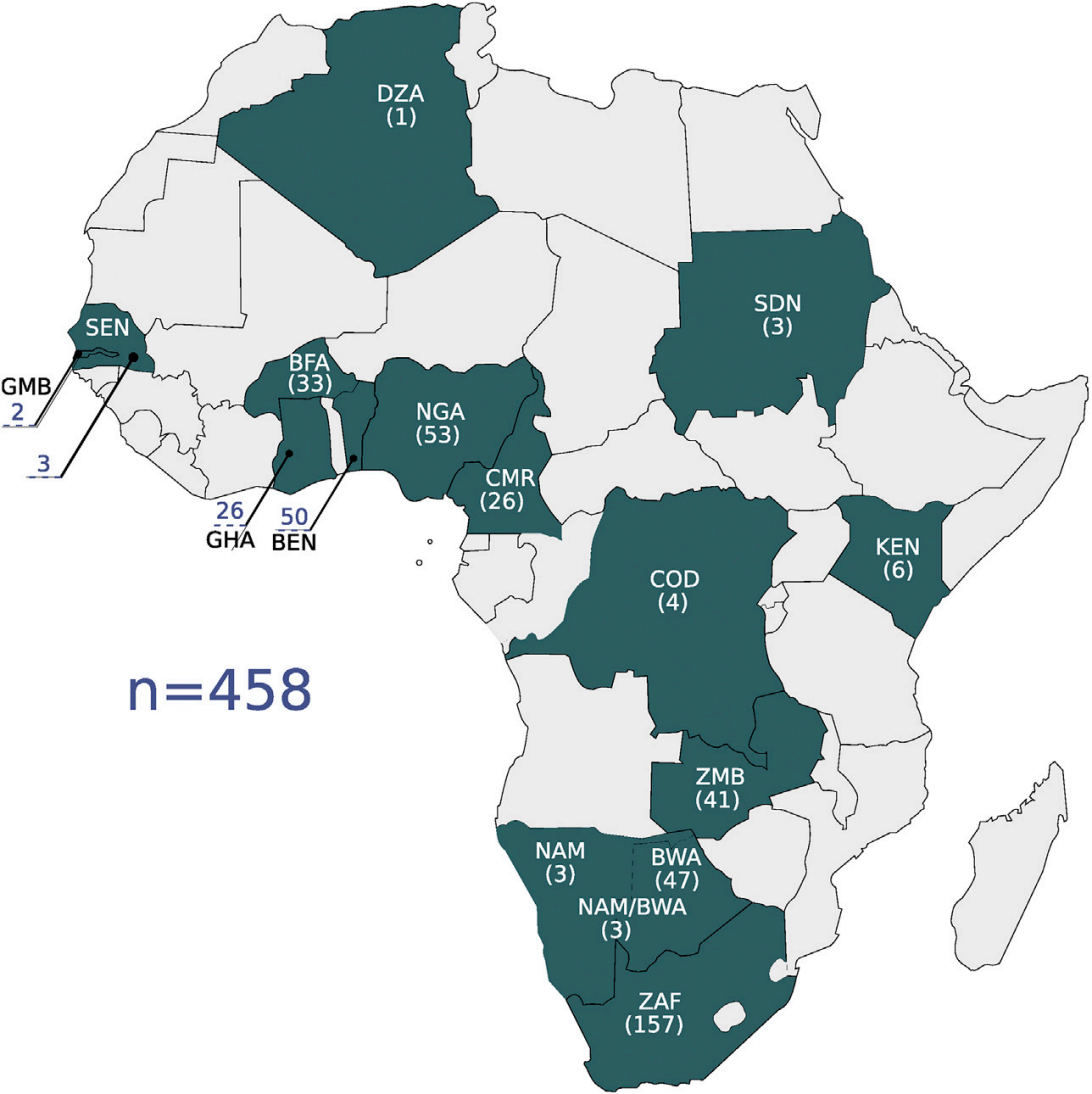
The geographic locations of the high coverage WGS data are shown on the map. Countries are referenced by their ISO 3166-1 alpha-3 code: BEN, Benin; BFA, Burkina Faso; BWA, Botswana; CMR, Cameroon; COD, Democratic Republic of the Congo; DZA, Algeria; GHA, Ghana; GMB, Gambia; KEN, Kenya; NAM, Namibia; NGA, Nigeria; SDN, Sudan; SEN, Senegal; ZAF, South Africa; ZMB, Zambia. The number of samples per country is shown in parentheses.

Four hundred and fifty eight high coverage whole genome sequences were used in the study as the primary data set (we call this the high coverage African ADME Dataset—HAAD). Most sequences were generated by the Human Health and Heredity in Africa (H3A) consortium (The H3Africa Consortium, 2014; Choudhury et al., 2020) and we also used public data and additional novel data from collaborators—see Table 1 and Figure 1. The population structure of participants in this study is broadly representative of speakers of Niger-Congo languages from West through South Africa. Representation from Nilo-Saharan and Afro-Asiatic populations is sparse. There also are few individuals of Khoe and San heritage, although significant admixture from Khoe and San speakers is found in Bantu-speakers in Southern Africa (Choudhury et al., 2017).

**TABLE 1:** Sources of high-coverage data sets used to form HAAD: 272 genomes were generated by a supplementary grant from the NIH to the H3A Consortium (Choudhury et al., 2020) for the primary purpose of designing a custom genotyping array; 100 were produced by AWI-Gen; 40 were shared by African collaborators; 15 genomes came from the Southern African Human Genome Program (SAHGP), and 31 genomes were from the Simons Foundation Genome Diversity Project.

Country	H3A consortium data: High coverage research center	*n*
Benin	University of Montréal	50
Burkina Faso	AWI-Gen	33
Botswana	BHP	47
Cameroon	University of Dschang	26
Ghana	AWI-Gen	26
Nigeria	Institute of Human virology	49
South Africa	AWI-Gen	100
Zambia	University of Zambia	41
African collaborators: High coverage		
South Africa	SA Human genome program	15
South Africa	Cell biology research lab, NICD/Wits	40
Public data sets		
Various	Simons foundation	31

We supplement some analyses with African datasets from the KGP (we use KGA specifically to refer to the African genomes in KGP). As the KGP datasets are low coverage, not all analyses were performed with the KGA dataset in addition to HAAD.

### 2.2 Population Structure

**FIGURE 2:**
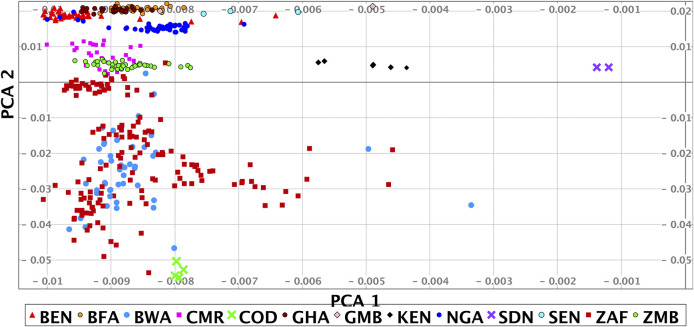
Principal component analysis of the HAAD (some outliers are omitted). Abbreviations for sources used are H3A (Human Health and Heredity in Africa Consortium), and SF (Simons Foundation Genome Diversity Project). The countries of origin and source of the samples shown in the PCA are: BEN/H3A, BFA/H3A, BWA/H3A + SF, CMR/H3A, COD/SF, GHA/H3A, GMB/SF, KEN/SF, NGA/H3A + SF, SDN/SF, SEN/SF, ZAF/H3A + Tiemessen Lab + SF + Southern African Human Genome Program, ZMB/H3A. Country codes given in Figure 1.

A principal component (PC) and structure analysis of our data shows high genome-scale variation and that we have good coverage of African genomic diversity across West, Central and Southern Africa, with less coverage in East Africa. The PC analysis of our data shows a strong correlation to geographical location (Figure 2 and Supplementary Section S1).

To explore diversity between different African regions we clustered the studied population together with reference data sets using PC data (see Methods, Table 2). The PC analysis shows that the HAAD samples fall broadly into three groups: West (Ghana, Burkina Faso, Nigeria), South/Central (Cameroon, Zambia, Botswana, South Africa), South (Botswana, South Africa) African populations. The variability in the Southern group primarily arises through differential admixture between Bantu, Khoe and San speakers. There is a Far West group comprising individuals in HAAD and KGA from Gambia, Senegal and Sierra Leone. There are also a few individuals from other African regions. Note that there is significant diversity within countries; and in some cases overlap between countries—e.g. some participants that we label as “South/Central” live to the south of some participants in the “Southern” group.

**TABLE 2:** Clusters within Africa, including the number of individuals in each cluster. Clusters include both HAAD and 1,000 Genomes African population data.

Identifier	Number	Region
SA	166	Southern Africa
SC	172	South/Central Africa
KS	5	Khoe and San
FW	185	Far West Africa
WE	309	West Africa
O	5	Outliers

### 2.3 Overall Characterization of ADME Variation

**FIGURE 3:**
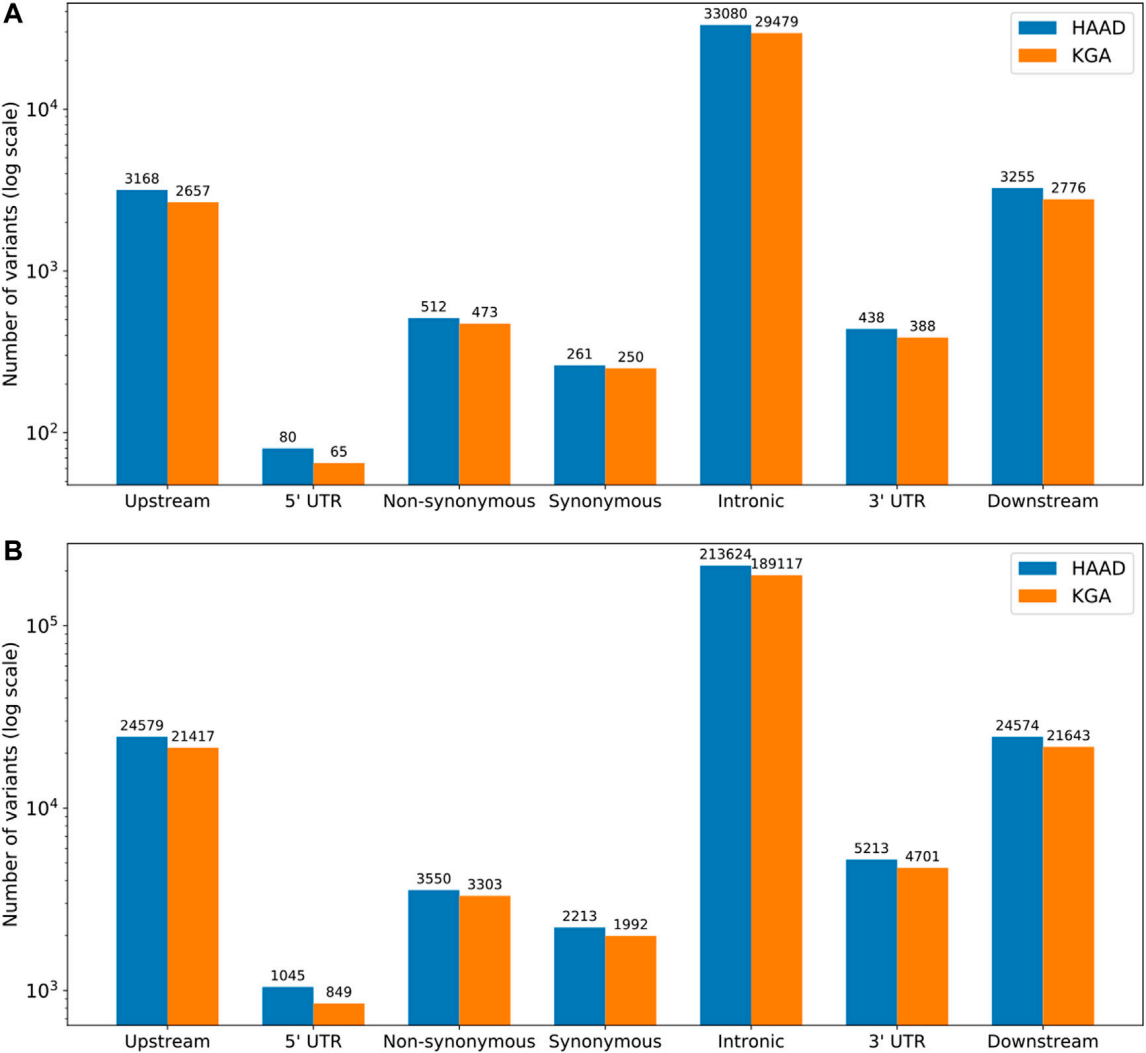
Distribution of variant types (as defined by SNPeff annotation) across core **(A)** and extended **(B)** ADME gene regions. HAAD (*N* = 458) represents those samples in the jointly called set from the H3A Consortium data, Simons Foundation, SAHGP and Tiemessen Labs, and KGA (*n* = 506) represents the African KGP populations from the jointly called set. Upstream and downstream regions are represented by 10 kb flanks from gene start and end respectively.

Gene-based genetic variation for the core and extended ADME gene categories was assessed for composition and type, including introns, upstream and downstream flanking regions (Figure 3). Comparisons were made between the HAAD dataset and the KGA dataset, which represent samples in the joint called HAAD and African KGP populations respectively (Methods 5.3.1). In ADME core genes, we counted a total of 40,714 and 36,088 variants for HAAD and KGA data respectively while for the extended ADME genes there were 274,798 and 243,022 variants respectively. Intronic variants are most common overall with about the same proportions in both HAAD and KGA datasets of 80 and 77% (for both core and extended genes) respectively. A significant number of variations appear in 3′ untranslated (3′ UTR) and 5′UTR regions. Coding region variants (non-synonymous and synonymous as annotated by VEP v92.0) do not overlap completely between HAAD and KGA groups. For core genes there were 423 coding variants common to both HAAD and KGA datasets, 288 coding variants unique to HAAD, and 252 unique to KGA. For extended genes, there were 17,148 coding variants common to HAAD and KGA, 2,850 unique to HAAD, and 2,318 unique to KGA respectively. Care should be taken in comparing HAAD and KGA data because of the different depth of sequencing.

**FIGURE 4:**
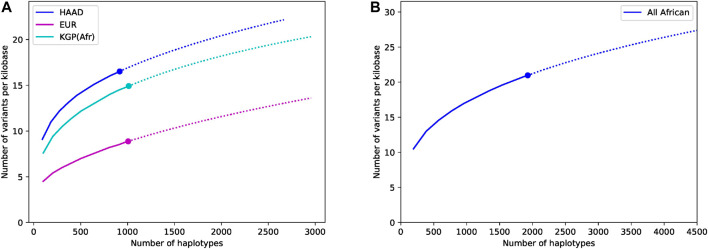
Comparative discovery curves of variants in the core ADME genes (including flanks) for the **(A)** HAAD, the 1,000 Genomes African and European data, and **(B)** in the combined HAAD and KGA datasets. The results show for a given number of haplotypes the number of variants seen per kilobase. The actual results are shown as a large dot, sub-samples by a solid line, and projections by a dotted line. Sub-samples values are computed averaging over 50 different randomly sampled subsets for intermediate values. Projection is computed using a third order jackknife projection (Burnham and Overton, 1979).

The importance of using and generating African datasets like ours can be seen in our discovery curves which show the increase in the number of variants found in the core ADME genes as more genomes are included in the study (the results for the extended genes are not shown but are similar). Figure 4A compares our data set to 1,000 Genomes African and European populations. The diversity of African populations compared to European populations is clear and consistent with previous literature (Hovelson et al., 2017). We believe that the increased richness of our data compared to 1,000 Genomes African data is partially due to the fact that our data is high-coverage. This richness is also likely to be driven by the significant numbers of Southern African genomes that have significant Khoe and San ancestry (see (Choudhury et al., 2017) for some discussion) as well some diverse samples from the Simons Foundation. Figure 4B shows the discovery curve for the combined African (HAAD and KGA) dataset. Although the curve has started to plateau, the results show that combining the data sets has value, and that sampling more Africans and more diverse African groups not yet properly captured will reveal considerably more variants.

### 2.4 Annotation of High Impact Coding Variants

To annotate ADME genes we used the output of an ADME gene optimized annotation schema. This schema uses five prediction tools, and variants meeting score cutoffs for all five are of the highest confidence for functional impact. We identified 930 high impact variants (HI-vars) (Supplementary Table S8) for 247 ADME genes (from a total of 299 ADME genes) of which 29 are core genes and 218 are extended genes. Of the core genes, seven members of the cytochrome P450 (CYP450) family (*CYP1A1*, *CYP1A2*, *CYP2B6*, *CYP2C8*, *CYP2C19*, *CYP2A6*, *CYP2D6*) were among those with the highest count of high impact variants. Highest counts of the CYP450 genes were seen in *CYP1A1* and *CYP2D6* with 12 and 10 HI-vars of respectively. The ATP-Binding Cassette (ABC) transporter gene, *ABCB5* showed the highest number of HI-vars overall numbering 20. We also counted three members of ABCC transporter family and three other members of the CYP450 family in the 10 most variable genes.

**FIGURE 5:**
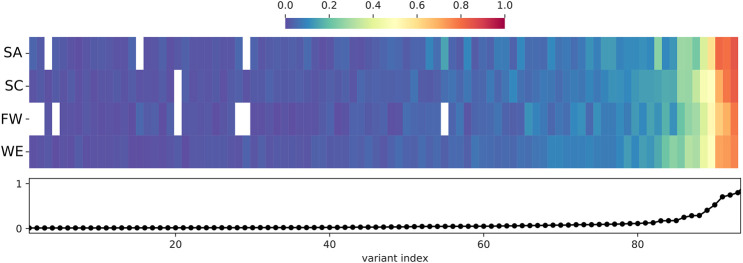
Distribution and frequency of HI-vars across sub-populations. Only common HI-vars (Frequency > 1% in the total joint called population dataset) are represented in the figure. Frequency is that of the non-reference (ALT) allele. The frequency per variant is reported in the lower panel of the figure as a line plot (indexed by frequency). The frequency of each of the variants in each sub-population is given by each heat map column, with white indicating 0% frequency. See Table 2 for abbreviations.

The 930 HI-vars are mostly rare alleles, with most being singletons or doubletons. There were only 93 variants with a frequency above 1% in the total joint called samples (Figure 5). Overall, the frequency distributions for sub-populations (SA, SC, FW, and WE) are not uniform (we omit discussion of the Khoe and San cluster because of low sample number). Some of the high impact variants tend to show a large disparity in frequency values between some clusters. For example, the *CYP27A1* rs114768494 variant (chr2:g.219677301C > T) (28th index in Figure 5) is only present in SC and WE with respective frequencies of 1.1 and 3.7%. Also, variants can exist in all the sub-populations but with significantly different proportions. For instance, the *CYP4B1* rs45446505 variant, (chr1:g.47279898C > T) (52nd index) is present at frequencies of 9.5, 2.3, 4.5 and 3.5% for SA, SC, FW, and WE respectively. Another variant: *CYP4B1* rs3215983 (ch1:g.47280747_47280747del) (47th index) is common in the SC population with a 10% frequency. This value is at least twice that of other clusters. Frequency differences of ≈10% are observed in common high impact variants.

**FIGURE 6:**
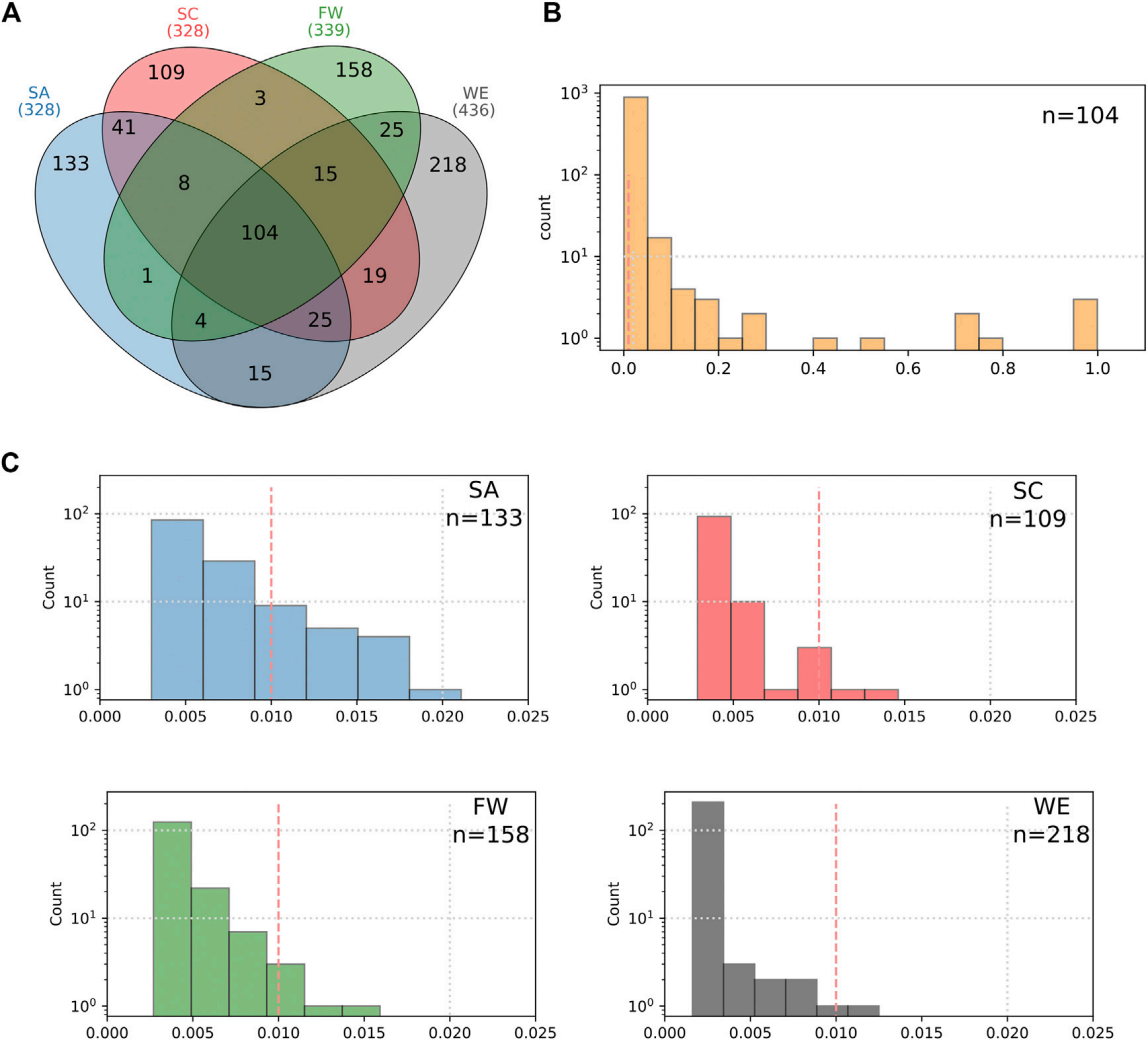
Characterization of the HI-vars in clusters SA, SC, FW, and WE. **(A)** The Venn diagram shows the overlap between the clusters for these variants. Distribution plots of the frequencies of the common variants between the four clusters **(B)** and for the unique variants found in each sub-population **(C)**.

The regional overlap of the total HI-vars identified shows the majority of these variants are appear in one population cluster only (Figure 6). There are only ∼100 variants that overlap all African population clusters. These variants appearing in all regions have widely ranging frequencies, with most falling between 1 and 20% for the total African samples assessed. Each population cluster had >110 variants specific to it. Variants that occur only in one cluster are mostly rare, with an average frequency of less than 1% in their own respective cluster. Southern Africans have 20 cluster-specific variants with frequencies above 1% (20 variant)—more than any other cluster. Relatively fewer variants overlap between two clusters alone, with a trend of geographically close clusters sharing more variants than those which are distant.

**FIGURE 7:**
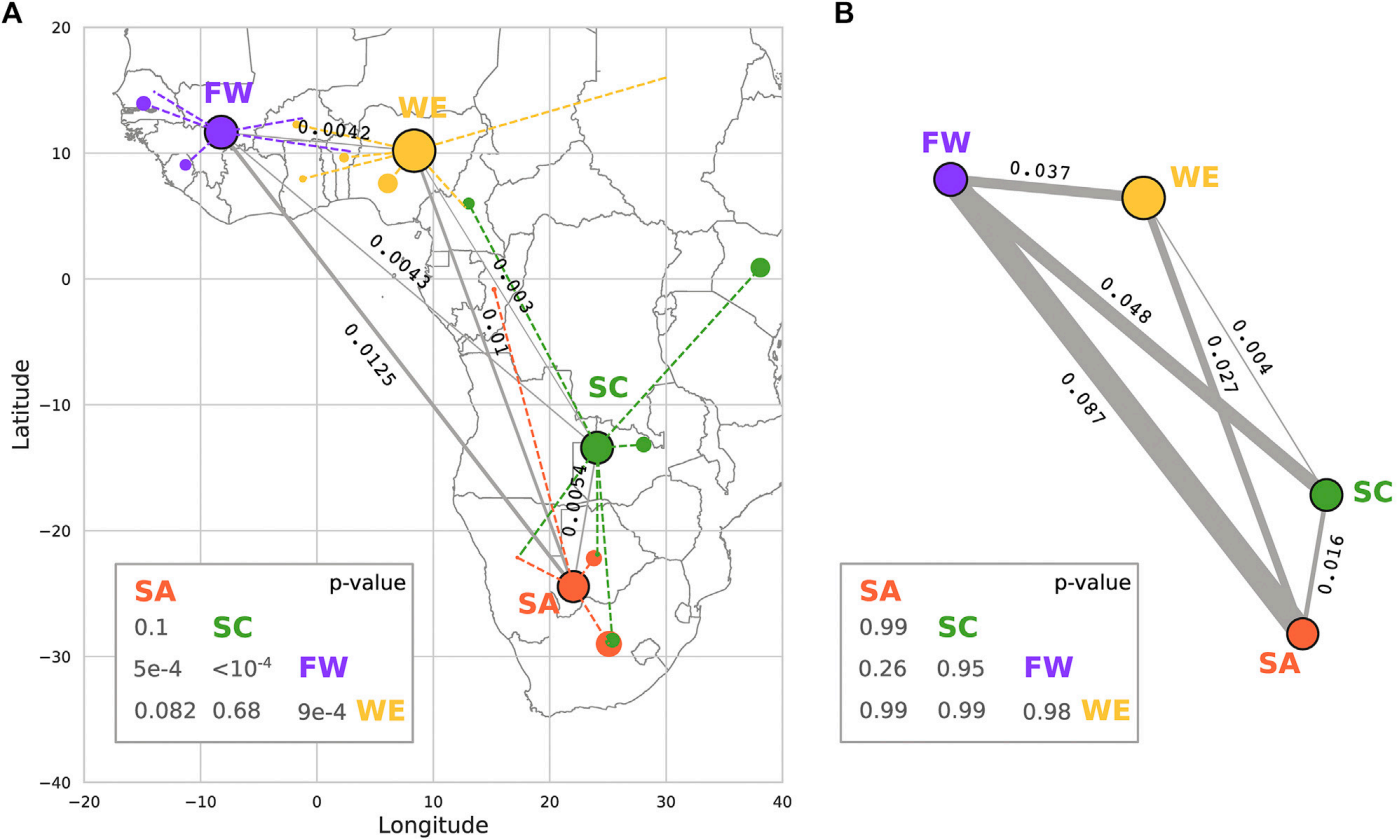
Calculation of Fixation index ( $F_{ST}$ ) between the population clusters. We used HI-vars **(A)** and all ADME variants **(B)** to compute the weighted $F_{ST}$ value between each pair of populations using PLINK (version v1.90b6.3). Population clusters are represented by the geographical centroid of the ensemble of country centroids constituting each cluster. Dashed lines link the centroids of the countries to the cluster centroid. Node radii are proportional to the size of each sample. *p*-values were calculated from a random $F_{ST}$ distribution by sampling 917 and 32,0983 variants of a random set of genes (*n* = 2,000) for high impact variants and all ADME variants respectively.

Fixation index ( $F_{ST}$ ) assessments revealed that there are inter-cluster differences calculated for HI-vars (Figure 7A), and for all ADME gene variants (Figure 7B). The greatest $F_{ST}$ of all ADME variants is observed between SA and FW populations (0.0125) and the lowest $F_{ST}$ is observed between SC and WE (0.003). For $F_{ST}$ calculated across HI-vars, these are specific to ADME HI-vars as compared to HI-vars identified in random genes across the genome (*n* = 2,000). This effect was significant between Far West Africans and all other clusters (*p* < 0.001). Despite being geographically close and having HI-vars in common, FW and WE clusters show an $F_{ST}$ value of 0.0042, similar to the $F_{ST}$ between the Far West FW and SC cluster, which are geographically distant and have no common variants—something meriting further study. Both of these differences show significant *p*-values of 9 × 10^−4^ and <10^−4^ between FW/WE and FW/SC respectively. $F_{ST}$ values for all ADME gene variants overall show higher levels of differences, none of which, however, seem to be a property of these variants compared to genetic variants from a random set of genes (all *p*-values are non-significant).

In summary, HI-vars are not uniform across African clusters, and that geographical proximity is not a proxy for genetic similarity in ADME genes.

### 2.5 CNVs

A copy number variant region (CNVR) is determined by aggregating overlapping copy number variants (CNVs) identified in different individuals. A total of 259 CNVRs were identified, consisting of 106 duplications, 106 deletions and 47 mixed CNVRs (i.e. a region that is deleted in some individuals and duplicated in others) (Table 3). Duplications were further separated into biallelic duplications (3 or 4 copies) and multi-allelic duplications (>4 copies). About 54% of CNVRs were unique, while the remaining CNVRs overlapped with one or more of the other CNVRs identified. Of the 299 ADME genes that were analyzed, a total of 116 genes (38.8%) contained at least one CNVR. These include some important core pharmacogenes such as the *CYPs*, *UGTs*, and *GSTs*. Furthermore, the number of CNVs in ADME genes per individual ranged from four to 71, with the majority of individuals (89.9%) harboring between 11 and 30 CNVs.

**TABLE 3:** CNVRs identified in core and extended ADME genes (percentages rounded to closest integer).

CNVR category	Total	ADME genes	
		Core	Extended
Deletions	106 (41%)	30	76
Biallelic duplications	71 (27%)	7	64
Multi-allelic duplications	35 (14%)	2	33
Mixed CNVRs	47 (18%)	16	31
Total	259	55 (21%)	204 (79%)

### 2.6 Novel and Highly Differentiated Variants

A novel variant in the context of this study is an SNV that is identified in the high coverage African population datasets, and not present in dbSNP (version 151) (Sherry, 2001) which aggregates variants from various data sources that include the 1,000 Genomes consortium (Altshuler et al., 2010; Auton et al., 2015), GO-ESP (Auer et al., 2016), ExAC consortium (Lek et al., 2016), GnomAD (Karczewski et al., 2019) and TOPMED (Brody et al., 2017).

A total of 343,606 SNVs were called for the ADME genes from the HAAD set of 458 samples, with 12% classified as novel SNPs (Supplementary Table S2). For the 32 core ADME genes, 5,818 novel variants were identified and a further 34,874 novel variants were identified in the 267 extended ADME genes within the HAAD. The majority of these variant types are intronic or intergenic variants (Supplementary Figure S4). Of the novel coding variants, eight were identified as HI-vars in core genes and 88 in extended genes.

The largest number of novel SNVs identified were from populations sampled from the Southern African region (not unexpected as there are no Southern African populations in the KGP). Novel variants in each regional population cluster were characterized according to their effect as summarized in Supplementary Table S3.

We compared the frequencies of ADME variants seen in the HAAD set as well as in at least one of the other large databases including 1,000 Genomes Consortium, ExAC, gnomAD and TOPMED. Any variant with a frequency two-fold more or two-fold less in the HAAD set than in the other datasets was considered as highly differentiated. Approximately 1,957 ADME variants were highly differentiated in the HAAD data compared to 1,000 Genomes consortium, ExAC, gnomAD and TOPMED datasets. Sixteen common variants with Minor Allele Frequency (MAF) ≥1% in eight core genes were more frequent in HAAD than in the KGP including African populations in those datasets. One variant in one of the core genes (rs3017670, *SLC22A6*) was seen more commonly in the other datasets than in the HAAD data (Supplementary Table S4). In total, 251 core and extended ADME genes harbored highly differentiated variants, with about 80% of them having at least two highly differentiated variants.

**FIGURE 8:**
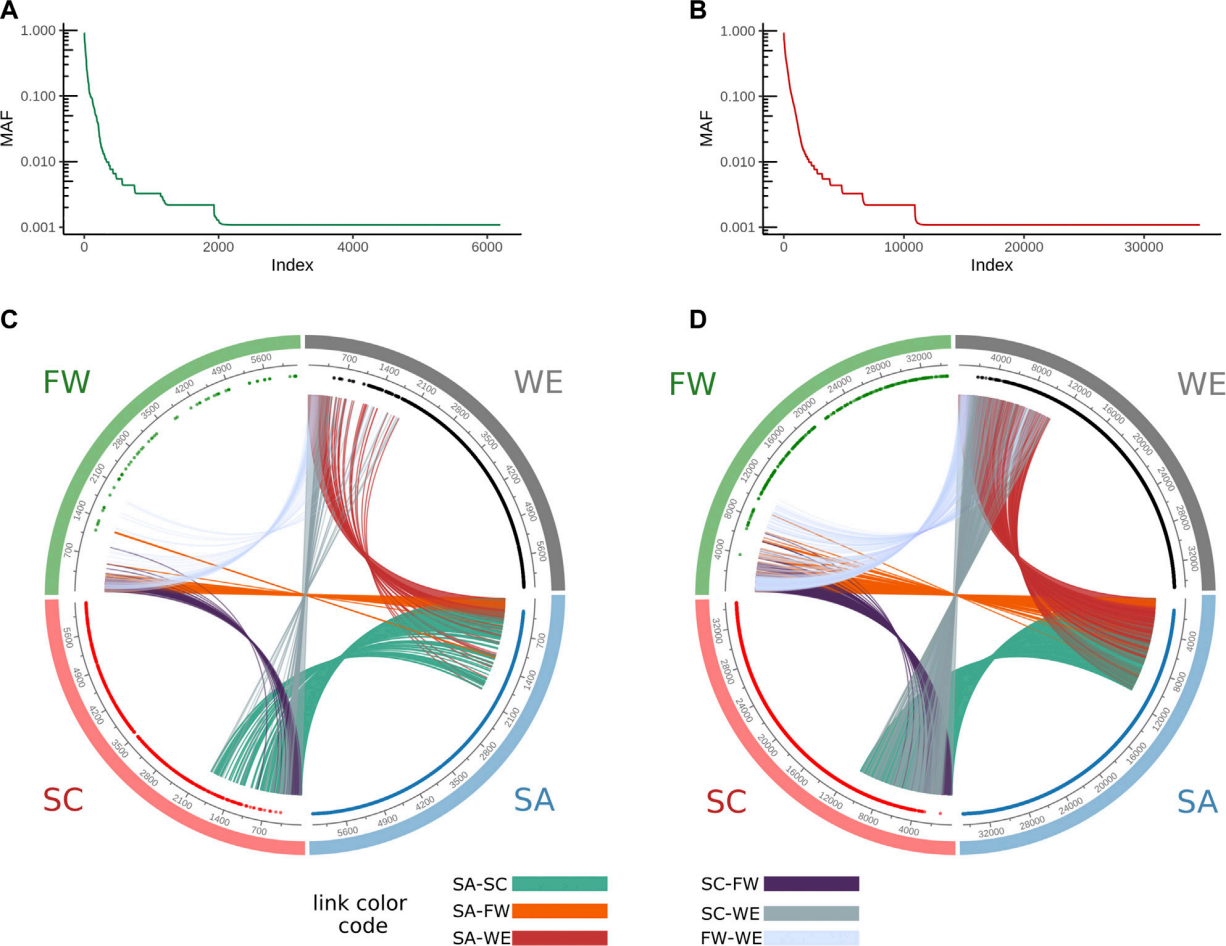
The number of novel high impact variants in ADME protein families. Abbreviation for Protein families: AD, aldehyde dehydrogenase; ANA, arylamine N-acetyltransferase; ATPDABZ, ATP-dependent AMP-binding enzyme; CATA, cation transport ATPase; CYP450, cytochrome P450; DADA, DAMOX/DASOX; DASS, SLC13A/DASS transporter; EPH, epoxide hydrolase; GPER, glutathione peroxidase; GSTA, GST alpha; GSTM, GST Mu; ICAD, iron-containing alcohol dehydrogenase; LAAT, L-type amino acid transporter; NCYP450, NADPH–cytochrome P450 reductase; NHRF, nuclear hormone receptor family; OCTF, organic cation transporter family; PEROX, peroxidase; SDR, short-chain dehydrogenases/reductases; ST2, sulfotransferase 2; SUGT, Sugar transporter; SULF1, sulfotransferase 1; UDPGT, UDP-glycosyltransferase. ABCC, ABCB, ABCA, FMO, ABCG are not abbreviated. On the structures, we showed examples of variants for four proteins belonging to different protein families described in Section 2.6.

We performed a structural analysis of four rare novel HI-vars in the *CYP2A13*, *CFTR*, *ABCB1*, and *NAT1* genes, all having a protein structure in the Protein Data Bank (Figure 8). A variant chr19:g.41595975C > G causes a substitution p. Arg123Gly on *CYP2A13* (PDB code 2PG5) (Sansen et al., 2007). Mapping this variant on the structure shows a position close to the interaction site belonging to a rigid alpha helix which might affect the binding properties and the local folding integrity.

For the *CFTR* gene, a chr7:g.11725069A > G causes a substitution p. Thr1036A which is involved in the interaction of the Lasso domain of the protein serving as a critical interaction segment of CFTR with other proteins (PDB code 6MSM) (Ford, 2017; Zhang et al., 2018). This threonine also appears to form a pseudoproline-like structure in which the side chain OH is hydrogen bonded to its own backbone NH. This may contribute to the bending of the helix in which this residue is found. Mutation to Ala removes this hydrogen bond and may therefore influence the degree of bending of this helix.

A p. H1232Q protein variant in ABCB1 could affect the interactions of this residue with the ATP molecule required for the active transport process (PDB code 6C0V) (Kim and Chen, 2018). In the structure His 1,232 lies in a site to which an ATP is bound approximately 5 Å from the ATP gamma phosphate. Although not in direct contact with the ATP, it could interact with it via a network of hydrogen bonds involving water molecules or, if the histidine is protonated, via an electrostatic interaction with the ATP phosphates. A mutation to Gln could affect both types of interaction with the ATP.

The chr8:g.18079983C > T variant creates a premature stop codon in NAT1 gene (PDB code 2IJA). The variant corresponds to the position p. Q143 which is close to the catalytic site of the protein.

**FIGURE 9:**
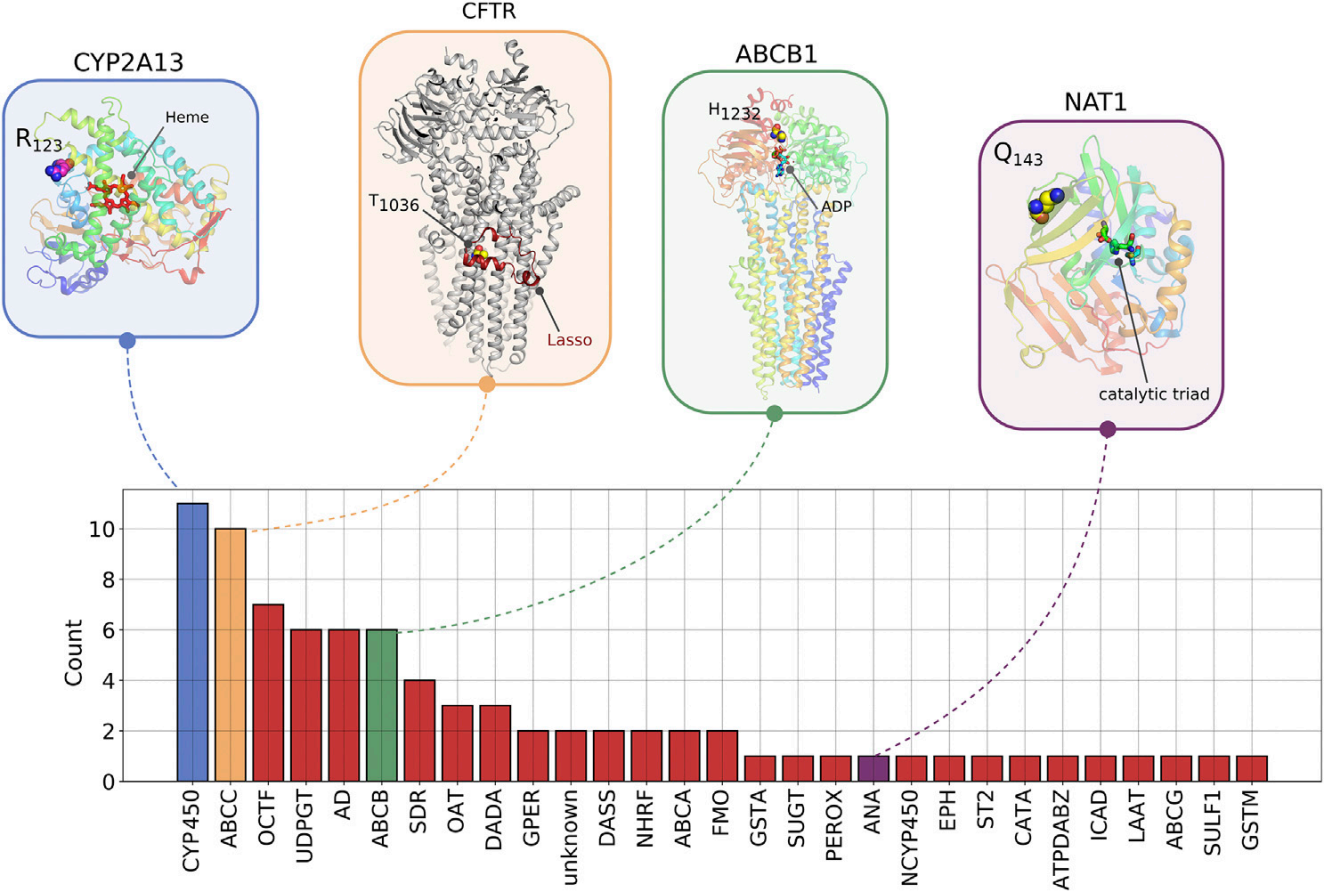
Characterization of novel variant distribution across the ADME genes in HAAD. Variants are indexed by descending MAF for core genes **(A)** and extended genes **(B)** for the total population of HAAD samples. Circular plots for core **(C)** and extended genes **(D)** show the position of the unique variants per each cluster across the index axes represented by points. The index represents the MAF of the variant in the total HAAD dataset. A link is established if two clusters share the same variant. The FW cluster has fewer variants in common with other clusters in this figure due to low sample number used to generate this figure.

We analyzed the distribution of the novel variants for the HAAD population cluster (Figure 9). The shared variants are generally exclusive for higher index values, which correspond to higher allele frequencies (Figures 9A,B) in their respective cluster for both core (Figure 9C) and extended genes (Figure 9D). Moreover, we noted that the cluster specific variants cover a big portion of the frequency spectrum: most of them are rare (lower limit of the frequency spectrum).

### 2.7 Potential Translational Impact of ADME Pharmacogenomic Variants With Known Clinical Effects

To assess the transferability of variants with known pharmacogenomic effect, we focused on variants with PharmGKB level 1A and 1B clinical annotations. A level 1A annotation denotes a variant-drug combination published as a CPIC guideline or known clinical implementation in a major health system, while a level 1B annotation denotes a variant-drug combination for which a large body of evidence shows an association in the context of altering drug response (Whirl-Carrillo et al., 2012) (Note that the absence of level 1 annotation may be evidence of lack of study of a variant, especially for African-specific variants, rather than evidence against clinical relevance.) There are 21 clinical variants (PharmGKB 1A/B) in total in 11 ADME genes in the entire HAAD set: nine of these variants had AF ≥ 0.05 in HAAD, while 12 are rarer (AF < 0.05); and 7 of the 21 are HI-vars (rs3918290, rs1142345, rs28399504, rs4986893, rs1799853, rs3892097, rs1065852). We compared the frequency of the clinically actionable ADME gene variants in the combined HAAD population with that in the 1,000 Genomes super populations and gnomAD (Table 4). CYP2D6*17 (rs28371706, AF = 0.23) had much higher frequency in HAAD compared to the non-African KGP super populations and the combined gnomAD population. CYP2D6*17 has been associated with decreased *CYP2D6* enzymatic activity in African Bantu populations (Masimirembwa et al., 1996). The CYP3A5*3 defining variant (rs776746, splice acceptor) had a much lower frequency in HAAD (AF = 0.17), suggesting differences in the functionality of CYP3A5 in African populations compared to non-African populations where the frequency of this function-obliterating variant is higher.

**TABLE 4:** Allele frequency of the clinically actionable variants (PharmGKB 1A/B) in the combined HAAD dataset compared to the KGP super populations^*k*^ as well as gnomAD.

dbSNP id	Gene/Star allele	Variant type	Allele frequency						
			HAAD	^*k*^AFR	^*k*^AMR	^*k*^EUR	^*k*^EAS	^*k*^SAS	gnomAD
rs35742686	CYP2D6 (*3)	Frameshift	0	0.0023	0.0058	0.0189	0	0.002	0.0124
rs3892097	CYP2D6 (*4)	Splice acceptor	0.0376	0.0605	0.1297	0.1859	0.002	0.1094	0.1384
rs5030655	CYP2D6 (*6)	Frameshift	0	0.0008	0.0029	0.0199	0	0.001	0.0079
rs1065852	CYP2D6 (*10)	Missense	0.0843	0.113	0.148	0.202	0.571	0.165	0.209
rs28371706	CYP2D6 (*17)	Missense	0.2306	0.218	0.009	0.002	0	0	0.014
rs28371725	CYP2D6 (*41)	Intron variant	0.01	0.0182	0.062	0.0934	0.0377	0.1217	0.0805
rs1799853	CYP2C9 (*2)	Missense	0.0022	0.0083	0.0994	0.1243	0.001	0.0348	0.0926
rs1057910	CYP2C9 (*3)	Missense	0.0055	0.0023	0.0375	0.0726	0.0337	0.1094	0.0636
rs12777823	Intergenic	Intergenic	0.2544	0.251	0.107	0.151	0.314	0.362	0.189
rs12769205	CYP2C19 (*2)	Intron variant	0.1845	0.1967	0.1052	0.1451	0.3125	0.3579	0.1804
rs4244285	CYP2C19 (*2)	Synonymous	0.1463	0.1702	0.1052	0.1451	0.3125	0.3579	0.1759
rs4986893	CYP2C19 (*3)	Stop gained	0.0011	0.0023	0	0	0.0556	0.0123	0.0052
rs28399504	CYP2C19 (*4)	Start lost	0.0011	0	0.0029	0.001	0.001	0	0.0023
rs56337013	CYP2C19 (*5)	Missense	0	—	—	—	—	—	<¡0,001
rs72552267	CYP2C19 (*6)	Missense	0	—	—	—	—	—	<¡0,001
rs41291556	CYP2C19 (*8)	Missense	0	0.0008	0	0.003	0	0.001	0.0015
rs12248560	CYP2C19 (*17)	Upstream gene variant	0.1954	0.2352	0.1196	0.2237	0.0149	0.136	0.205
rs776746	CYP3A5 (*3)	Splice acceptor	0.1681	0.18	0.7968	0.9433	0.7133	0.6677	0.736
rs3745274	CYP2B6 (*6)	Missense	0.3734	0.374	0.373	0.236	0.215	0.381	0.272
rs2108622	*CYP4F2*	Missense	0.0415	0.0825	0.2378	0.2903	0.2143	0.4131	0.2735
rs3918290	*DPYD*	Splice donor	0.0011	0.001	0.001	0.005	—	0.008	0.006
rs115232898	*DPYD*	Missense	0.0153	0.0227	0.0029	0	0	0	0.0016
rs116855232	*NUDT15*	Missense	0	0.0008	0.0447	0.002	0.0952	0.0695	0.0281
rs1800462	TPMT (*2)	Missense	0	0.0008	0.0058	0.006	0	0	0.0017
rs1142345	TPMT (*3A and C)	Missense	0.0448	0.0666	0.0576	0.0288	0.0218	0.0174	0.0366
rs1800460	TPMT (*3A and B)	Missense	0	0.003	0.0403	0.0278	0	0.0041	0.0281
rs1800584	TPMT (*4)	Splice acceptor	0	—	—	—	—	—	<0.001
rs887829	*UGT1A1*	Upstream gene variant	0.4858	0.4932	0.379	0.2982	0.13	0.4366	0.364
rs4149056	*SLC O 1B1*	Missense	0	0.0136	0.134	0.161	0.123	0.0429	0.1326
rs115545701	*CFTR*	Missense	0.0066	0.0189	0.0014	0.001	0	0	0.0014
rs11971167	*CFTR*	Missense	0.0055	0.0182	0.0014	0.001	0	0	0.0012
rs202179988	*CFTR*	Missense	0	0.0008	0	0	0	0	<0.001

AFR: African, European: EUR, AMR: Ad Mixed American, EAS: East Asian, SAS: South Asian.

Some clinically actionable ADME gene variants common in the non-African KGP super-populations are rare in the HAAD set. These include the variants *SLCO1B1* rs4149056 (SLCO1B1*6), *CYP4F2* rs2108622, *CYP2D6* rs3892097, *CYP2C9* rs1799853, and *CYP2C9* rs1057910 (Table 4).

**FIGURE 10:**
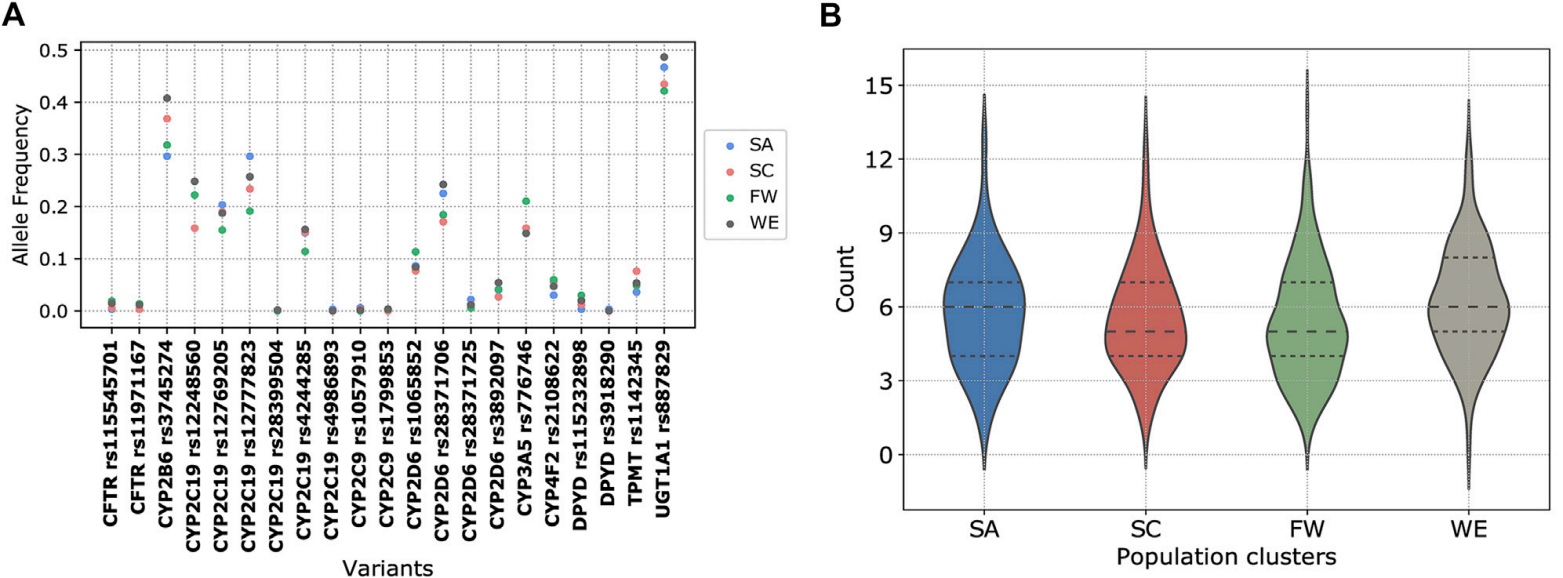
Distribution of pharmacogenomic variants with a high level of clinical annotation (PharmGKB level 1A/B). **(A)** Scatter plot of allele frequency of clinically relevant variants in the PCA clusters. **(B)** Violin plot of the number of clinically relevant variants carried per individual grouped by the population clusters.

Furthermore, we evaluated the distribution of level 1A/B PharmGKB variants within the African populations (HAAD and KGP) grouped according to the PCA clusters. Variants which show considerable frequency differences among clusters (SA, SC, FW, and WE) include CYP2B6*6 (rs3745274), and CYP2D6*17 (rs28371706) (Figure 10A). The number of level 1A/B PharmGKB variants per individual ranged from 0 to 15 (median of 6, 5, 5, and 6 in SA, SC, FW, and WE respectively) (Figure 10B), with 99.8% of individuals carrying at least one such variants.

### 2.8 Coverage of ADME Variants on SNV Genotyping Arrays

To evaluate whether genotyping array chips are suitable for detection of relevant ADME variants in African populations, we compared our whole genome sequencing variants with those captured by current arrays. Table 5 on page five shows the coverage of the variants that we detected in the core ADME genes in the WGS data compared to the Illumina Human Omni 2.5.8 (Omni) and the Illumina Infinium Multi-Ethnic AMR/AFR-8 Kit (MEGA). The Omni is a 2.39 million SNP array commonly used in human GWAS work—previous unpublished work shows that this is one of the best performing arrays on African populations. The MEGA array is 1.43 million SNP array optimized for African and Hispanic American populations (and can be augmented with approx. 200 k user selected SNPs). For different minor allele frequencies of variants we detected (MAF) we show the number of variants that are at least at that threshold, the number of those variants captured by probes by the two arrays, and the percentage of the variants that are captured. As can be seen, even at relatively high frequencies, less than 5% of the variants are captured by the array for core genes, and less than 8% for extended genes. As expected the larger Omni does a better job. However, of the 93 common HI-vars, only 19 (20%) are on the Omni chip whereas 50 (54%) are on the MEGA.

**TABLE 5:** Variant coverage and overlap for core and extended gene variants detected in HAAD whole genome sequencing datasets vs those captured by the Omni 2.5.8 and the MEGA arrays.

	MAF	WGS	Omni		MEGA	
			Chip	% Cov		% Cov
Core genes	1	18,660	640	3.4	349	1.9
	2	13,835	574	4.1	298	2.2
	3	11,335	522	4.4	242	2.1
	4	9,714	457	4.7	204	2.1
	5	8,886	409	4.6	179	2.0
	10	6,271	262	4.2	135	2.2
Extended genes	1	120,660	11,457	9.5	5,743	4.8
	2	94,651	11,031	11.7	5,256	5.6
	3	80,585	10,588	13.1	4,937	6.1
	4	71,480	10,220	14.3	4,638	6.5
	5	64,475	9,859	15.3	4,414	6.8
	10	43,228	8,069	18.6	3,555	8.2

Chip, number of variants in the chip; % cov = the percentage of SNPs at that MAF in WGS data that are covered by the array; WGS, number of variants in the whole-genome data.

### 2.9 Other Analysis

We found novel variation in the regulatory regions of the ADME genes and large regions of homozygosity. Supplementary Sections S5, S6 present these analyses.

## 3 Discussion

Next generation technologies have provided PGx and precision medicine a major increase in their application for disease treatment and drug safety (Claussnitzer et al., 2020). ADME genes have been a focus due to their critical role in pharmacodynamics and pharmacokinetics. Our work presents the first study characterizing the PGx landscape of ADME genes in SSA using high coverage whole-genome sequencing data which has been collected from different sources. The study’s main aim was to assess the variability of ADME genes across Africa and if this could have a significant impact on protein function and other pharmacologic properties and thus the potential impact on drug response.

We focus mainly on four African clusters distinguished geographically and genetically as shown by the PC whole-genome analysis. Overall assessments of structural and regulatory variation were evaluated across the complete dataset, while coding variants were assessed for functional impact. The applicability of known clinical variants and current genotyping technologies was also assessed.

In both novel variant and HI-vars analysis, our study demonstrates a significant level of variability. Most of the variants are rare and are population-specific in accordance with previous studies due mainly to increased population size and to a weak negative selection (Tennessen et al., 2012; Whirl-Carrillo et al., 2012; Nagasaki et al., 2015; Wright et al., 2018). Our high coverage data are adequate to genetically characterize these types of variants at high confidence levels. Evaluations of the false discovery rate of rare variants were previously estimated between 3.6 and 6.3% depending on the platform (Wall et al., 2014). Therefore, a broad extrapolation from our results is that there are between 30 and 60 false positive variants in our HI-vars. In the context of ADME pharmacogenes, although not all variants identified may prove to have functional impact, those that do may have significant consequences in dictating the drug-host response for individuals.

Our $F_{ST}$ calculation highlights the differences between clusters. Calculation using all ADME variants led to values similar to results obtained for multiple sub-Saharan African ethnic groups that used 328,000 independent SNPs (Busby et al., 2016). Genetic distance did not always correlate with geographical distance and in some pairs of clusters, the distance seems to be more significant in ADME genes. In the absence of clear evidence, it is not trivial to explain why two geographically close clusters like FW and WE, share a comparable degree of divergence like the pair FW-SC. Therefore, using ethno-geographical properties as a proxy to discriminate the PGx landscape might be inaccurate.

In addition, the important number of cluster-specific novel and high impact rare variants suggest that strategies limited to studies of high-frequency alleles might be considered as an over-generalization to a more complex pharmacogenomic landscape in SSA. In fact, our work highlights a “genetic diversity bottleneck” for precision medicine applications, requiring a balance between variants useful for population-based applications (for a particular cluster of Africans) and between the potential impact posed by variants unique to the individual. Therefore, the complexities of variant interpretation and reporting in PGx testing (Mills et al., 2013) may be exacerbated by the complex African ADME landscape.

While some clinically actionable variants have similar frequencies in European and African populations, our assessment of the top-level clinically validated variants shows a PGx knowledge bias toward European populations, with most variation in African and other global populations still largely uncharacterized in terms of PGx effect. Most are more common in Europeans, though some variants show an opposite trend, such as the CYP3A5*3 rs776746 (less common in Africans compared to Europeans) and CYP2D6*17 rs28371706 (largely African-specific). These enzymes are known to be key metabolisers of a large number of drugs, and these two variants (as they are common) will impact the reliability of using a European based PGx strategy in African populations. Key drugs that may be affected by those variants are codeine (Brousseau et al., 2007), primaquine (Awandu et al., 2018) (*CYP2D6*), and tacrolimus (Chen and Prasad, 2018) (*CYP3A5*). We also see an interesting example of *SLC O 1B1* rs4149056, which was seen in the KGA populations (albeit rarely), which is not seen in the HAAD samples. This further reiterates the need for additional African sequences, as publicly accessible African genomic data cannot remain represented by the KGP alone.

The greatest genomic coverage of African populations to date is available in genotyping array format (Gurdasani et al., 2015). These methods are unable to adequately characterize rare ADME variants at high confidence levels compared to high coverage WGS datasets. Moreover, we have also detected a large number of CNVs, and were able to do so robustly with our high coverage sequencing data as compared to other methods (Zhao et al., 2013). The distribution of CNVs and their impact on the ADME landscape in Africans is currently ongoing and will be available in a separate publication. As the state of data availability and type remains in flux, precision medicine approaches in Africa will be limited. In an ideal scenario, high coverage long read WGS will be used for more African samples undergoing clinical trials, as this allows for accurate resolution of haplotypes (including novel haplotypes), and thus, clearer interpretations of their potential impact on drug response.

## 4 Conclusion

Our work highlights that the ADME landscape in African populations is diverse, and shows the importance of rare variation held within individual population clusters. Therefore, current array-based genotyping technologies have severe limitations to be applied as the high throughput method in precision medicine applications. As sequencing technology becomes more accessible and cheaper, characterization of rare variants would benefit from the ongoing progress. Targeted sequencing and whole-exome sequencing would be better suited for characterizing ADME genes. Moreover, a previous suggestion to consider intra-ethnic genetic characterization in drug-development (Soko et al., 2018) might not be appropriate for SSA due to the important presence of singletons and the subjective assigning of ethnicity for individuals. The “genetic diversity bottleneck” in precision medicine might increase the burden of developing targeted therapies at sub-population levels because of the weak presence of common genetic patterns. However, these patterns might exist at the functional and phenotypic levels which might help to stratify the populations to clusters sharing common pharmacokinetic properties for a given drug. In this context, a proposed plan would integrate genotypic and phenotypic data into predictive models to unveil these patterns (Dandara and Matimba, 2019).

Capacity building efforts for PGx research in Africa is important. Strategies and policies for development of science and technology must ensure a future where Africa can take an active role in harnessing the power of genomic research in addressing its healthcare challenges. Promising positive steps are being taken with the establishment of initiatives such as the Human Heredity and Health in Africa project (http://h3africa.org/) that aims at strengthening research capacity for genomics in Africa.

### 4.1 Limitations

There are many limitations of our work. The most obvious is the need for significantly more genomic data from Africa. Although, more samples are necessary generally, there is a particular need for more diverse sampling. We focus on SSA, omitting northern Africa completely. We only had limited numbers of samples from Nilo-Saharan and Afroasiatic language speakers as well as speakers of non-Bantu languages in central, southern and eastern Africa (such as San and Khoe speakers). However, with more samples, we expect our conclusions to hold and the additional benefit would be a clearer resolution of the PGx landscape in diverse sub-clusters. Ideally, we would have merged the 1,000 Genomes African data and the HAAD data set and done a combined analysis. However, the bulk of the 1,000 Genomes WGS is low-coverage while the HAAD set is high-coverage which complicates comparative work significantly. As more data becomes available, this challenge will become easier. The discovery curve shown in Figure 4B shows we can expect to find many more variants when they are sequenced. Besides the lack of genomic data, there is very little clinical and drug response data for African populations (despite the effects of groups of excellence across Africa we have cited). Without this it will be difficult to associate the functional effect of variants to the clinical phenotypes. All of this costs money and requires scarce skills. Collaborations like ours, which has brought a diverse group of African scientists together show the potential of what can be done.

### 4.2 Strengths

Our work investigates novel African datasets and combines these with established African sequences to assess as broad an overview of African ADME variation as possible. This work could lay the foundations for motivation of more PGx related studies in Africans. We applied diverse computational assessment methods to mine the data and retrieve valuable genomic information. This can assist in guiding future research in resource scarce environments.

### 4.3 Future Work

In our subsequent work, we plan to do linkage disequilibrium analysis and haplotype frequency determination of key ADME genes—an example being *CYP2D6*, which requires specialized algorithms for accurate haplotyping (Twesigomwe et al., 2020). We are currently in the stage of resequencing *CYP2D6* and some other ADME genes with long-read sequencing technology to explore such features, as well as for in-depth analysis of CNVs.

## 5 Methods

### 5.1 Data

*H3A Consortium set* contains 272 samples selected and sequenced for the Human Heredity and Health in Africa (H3Africa) project. Samples cover populations from Benin, Burkina Faso, Botswana, Cameroon, Ghana, Nigeria and Zambia. Samples were shipped to the Human Genome Sequencing Center (HGSC) at Baylor College of Medicine (BCM), Houston, United States, under signed material transfer agreements from each project. Samples were prepared using the TruSeq Nano DNA Library Prep Kits and underwent whole genome sequencing on an Illumina TenX (150 bp) to a minimum depth of coverage of 30×.

*AWI-Gen set* consists of 100 South Eastern Bantu-Speakers (40× coverage).

*Cell Biology Research Unit, Wits set* consists of 40 samples from Soweto/Johannesburg South Africa (39 black and one mixed ancestry). Library preparation and sequencing was done at Edinburgh Genomics, Edinburgh, Scotland. Library preparation was done using the TruSeq Nano protocol and high coverage sequencing (∼30×) was done utilizing the Illumina SeqLab workflow system and the Illumina HiSeqX platform.

The *SAHGP set* is a collection of 15 samples from the Southern African Human Genome Program (Choudhury et al., 2017). Two main Bantu-speaking ethno-linguistic groups were included: The Sotho (Sotho-Tswana speakers; *n* = 8) and the Xhosa speakers (Nguni language; *n* = 7 recruited from the Eastern Cape Province). The DNA samples were normalized to ∼60 ng/μl and ∼5 µg DNA was submitted to the Illumina Service Center in San Diego, California, for sequencing on the Illumina HiSeq 2000 instrument (101 bp paired-end reads, ∼314 bp insert size) with a minimum read depth of coverage of 30× (Choudhury et al., 2017).

*SF set* contains 34 African samples selected from 300 individuals from the Simons Foundation Genome Diversity Project. Samples include populations from Congo, Namibia, Kenya, Senegal, Algeria, Nigeria, Gambia, Sudan and South Africa. Samples were sequenced at an average depth of 43× at Illumina Ltd.; almost all samples were prepared using the same PCR-free library preparation (Mallick et al., 2016).

*KGA set* consists of 507 African samples from the KGP. These samples include Gambian Mandinka, Mende from Sierra Leone, Yoruba from Ibadan, Nigeria, Esan from Nigeria and Luhya from Webuye, Kenya. Libraries were constructed on either Illumina HiSeq2000 or GAIIX with the use of 101 base pair end reads. Sequencing was done at an average depth of 4× (Auton et al., 2015).

The only phenotype made available to us was sex. In particular, self-identified ethnicity, location in the country, and disease status were not revealed.

### 5.2 Data Processing

From the BAMs we called gVCFs using HaplotypeCaller in gVCF mode using GATK v4.0.8.1. We combined all the gVCFs into one combined gVCF using GATK’s CombineGVCF (v4.0.8.1). From the combined gVCF we did joint calling using GenotypeGVCFs (v4.1.3.0) and followed GATK’s best practice for variant quality score recalibration for SNPs and INDELs. After applying VQSR we filtered for all the high quality (PASS) sites and used the VCF. The final VCF was used for downstream analysis. All code can be accessed at https://github.com/h3abionet/recalling.

### 5.3 Population Structure

Population structure was computed using the autosomal data in our samples together with reference data sets in order to ensure a relatively unbiased structure. We included all KGP African data, and two non-African KGP sets (Utah residents (CEPH) with Northern and Western European ancestry—CEU—and Bengali in Bangladesh—BEB) and some chip data from various projects including Khoi-San data (Schlebusch et al., 2012). The CEU and BEB populations were included to identify overall outliers in the African population groups. Prior work of our group has shown that the primary admixture from non-African populations, particularly in Southern Africa, comes from Europe and the Indian subcontinent. The BEB and CEU are good representatives for this study as other European or Indian populations. Only unambiguous, biallelic SNPs (A/C, A/G, C/T, G/T) common in all data sets were used. The data was merged and pruned using PLINK (Chang et al., 2015), leaving 401 k SNPs for analysis. Principal components were computed using PLINK and structure charts were produced using ADMIXTURE (Alexander et al., 2009) (30 independent runs for each value of *k*) and averaged using CLUMPP (Jakobsson and Rosenberg, 2007). All charts were produced with Genesis (Buchmann and Hazelhurst, 2014).

Population clusters were determined from the PCA values rather than from the project and self-identification labels due to overlapping data. The optimal number of clusters was determined using the method of Solovieff et al. (Solovieff et al., 2010), and clusters determined using *k*-means clustering with the R MASS package (Venables and Ripley, 2002). In analyses in which population clusters were compared, we only used the samples that appeared in the clusters (e.g., excluding Algerian, San samples). In all other analyses all the data was used. Choudhury et al. (Choudhury et al., 2020) discusses the population structure of the H3A data in more detail.

### 5.4 ADME Gene Selection

ADME genes as defined by PharmADME (http://pharmaadme.org) (both core and extended definitions) were extracted using current genomic co-ordinates for GRCh37. p13, as obtained through BioMart (Smedley et al., 2009). Gene flanking regions were included in the extraction (10,000 bp upstream from gene start and downstream from gene end).

### 5.5 Annotation and Functional Prediction

Variants were classified and typed using SnpEff v4.3t (Cingolani et al., 2012) with the GR37Ch base reference for canonical gene transcripts. Variant Effect Predictor (VEP) v92.0 (McLaren et al., 2016) was used for functional prediction based annotation. VEP was configured with dbNSFP v3.0 (Liu et al., 2016), a large database used to retrieve functional prediction scores for coding variants. The annotation analysis is implemented in g_miner workflow (https://github.com/hothman/PGx-Tools/tree/master/workflows/g_miner). An optimized model for functional prediction of pharmacogene variants produced by Zhou et al. (Zhou et al., 2019) was used as the basis for high impact classification of missense variants. The model uses five toolsets (LRT, MutationAssessor, PROVEAN, VEST3, and CADD). Loss of Function variants were classified as high impact if they were present in the canonical transcript of the gene. Singleton or doubleton high impact variants were filtered based on their VCF QUAL scores, using a cutoff of >50. Any variant that did not match such criteria was removed prior to subsequent analyses with bcftools v1.9 (Li, 2011). Three HI-vars were not displayed in Figure 5 due to incorrect reference alleles inducing an erroneous frequency: *ALDH3B1* rs11433668, and rs58160034; and *ADH1C*—rs283413. We have checked these variants in KGP and gnomAD datasets to validate the error.

### 5.6 Fixation Index ( $F_{ST}$ ) Analysis Between Population Clusters

Differences between African subgroups were calculated by PLINK v1.9 (Chang et al., 2015), using mean, weighted $F_{ST}$ between each pair of the population clusters. Prior to the calculation we applied linkage disequilibrium (LD) based pruning using PLINK v1.9 for different sets of variants: High Impact ADME, High Impact non-ADME, all ADME gene regions, and a set of 2000 random non ADME genes. The parameters used for this step are as follows: window size = 1,000; step size = 5 and variance inflation factor = 2.

### 5.7 CNVs

Discovery and genotyping of CNVs was performed using GenomeSTRiP’s SVPreprocessing and CNVDiscovery (svtoolkit 2.00.1918) pipelines using the default parameters for genomes sequenced at 30–40× coverage (Handsaker et al., 2015).

## Members of the Human Heredity and Health in Africa Consortium

Jorge da Rocha, Sydney Brenner Institute for Molecular Bioscience, Faculty of Health Sciences, University of the Witwatersrand, Johannesburg, South Africa, and Division of Human Genetics, National Health Laboratory Service, and School of Pathology, Faculty of Health Sciences, University of the Witwatersrand, Johannesburg, South Africa; Houcemeddine Othman, Sydney Brenner Institute for Molecular Bioscience, Faculty of Health Sciences, University of the Witwatersrand, Johannesburg, South Africa; Gerrit Botha, Computational Biology Division and H3ABioNet, Department of Integrative Biomedical Sciences, University of Cape Town, South Africa; Laura Cottino, Sydney Brenner Institute for Molecular Bioscience, Faculty of Health Sciences, University of the Witwatersrand, Johannesburg, South Africa, and Division of Human Genetics, National Health Laboratory Service, and School of Pathology, Faculty of Health Sciences, University of the Witwatersrand, Johannesburg, South Africa; David Twesigomwe, Sydney Brenner Institute for Molecular Bioscience, Faculty of Health Sciences, University of the Witwatersrand, Johannesburg, South Africa, and Division of Human Genetics, National Health Laboratory Service, and School of Pathology, Faculty of Health Sciences, University of the Witwatersrand, Johannesburg, South Africa; Samah Ahmed, Centre for Bioinformatics and Systems Biology, Faculty of Science, University of Khartoum, Sudan; Faisal M. Fadlelmola, Centre for Bioinformatics and Systems Biology, Faculty of Science, University of Khartoum, Sudan; Philip Machanick, Department of Computer Science, Rhodes University, Makhanda, South Africa; Mamana Mbiyavanga, Computational Biology Division and H3ABioNet, Department of Integrative Biomedical Sciences, University of Cape Town, South Africa; Sumir Panji, Computational Biology Division and H3ABioNet, Department of Integrative Biomedical Sciences, University of Cape Town, South Africa; Clement Adebamowo, Institute for Human Virology, Abuja, Nigeria, and Institute of Human Virology and Greenebaum Comprehensive Cancer Center, University of Maryland School of Medicine, Baltimore, MD, United States; Mogomotsi Matshaba, Botswana-Baylor Children’s Clinical Center of Excellence, Gaborone, Botswanam, and Baylor College of Medicine, Houston, TX, United States; Michéle Ramsay, Sydney Brenner Institute for Molecular Bioscience, Faculty of Health Sciences, University of the Witwatersrand, Johannesburg, South Africa, and Division of Human Genetics, National Health Laboratory Service, and School of Pathology, Faculty of Health Sciences, University of the Witwatersrand, Johannesburg, South Africa; Gustave Simo, Molecular Parasitology and Entomology Unit, Department of Biochemistry, Faculty of Science, University of Dschang, Dschang, Cameroon; Martin C. Simuunza, Department of Disease Control, School of Veterinary Medicine, University of Zambia, Lusaka, Zambia; Scott Hazelhurst, School of Electrical and Information Engineering, University of the Witwatersrand, Johannesburg, South Africa.

## Members of the H3A/GSK ADME Collaboration

Britt I. Drögemöller, Department of Biochemistry and Medical Genetics, University of Manitoba, Winnipeg, MB, Canada; Galen E. B. Wright, Neuroscience Research Program, Kleysen Institute for Advanced Medicine, Winnipeg Health Sciences Centre and Max Rady College of Medicine, University of Manitoba, and Department of Pharmacology and Therapeutics, Rady Faculty of Health Sciences, University of Manitoba, Winnipeg, MB, Canada; Caroline T. Tiemessen, Centre for HIV and STIs, National Institute for Communicable Diseases, National Health Laboratory Services and Faculty of Health Sciences, University of the Witwatersrand, Johannesburg South Africa; Sandra Baldwin, Drug Metabolism and Pharmacokinetics, GlaxoSmithKline R&D, Ware, UK; Mathias Chiano, Human Genetics, GlaxoSmithKline R&D, Stevenage, UK; Charles Cox, Human Genetics, GlaxoSmithKline R&D, Stevenage, UK; Annette S. Gross, Clinical Pharmacology Modelling and Simulation, GlaxoSmithKline R&D, Sydney, NSW, Australia; Pamela Thomas, Data and Computational Sciences, GlaxoSmithKline R&D, Stevenage, UK; Francisco-Javier Gamo, Global Health, GlaxoSmithKline R&D, Madrid, Spain.

## Data Availability

The Simons Foundation and 1000 Genomes data are publicly available. The HAAD data set is controlled access data due to the need to protect participants, and is available from European Genome-Phenome Archive (https://ega-archive.org/) on application to the relevant Data Access Committee (EGADs EGAD00001003791, EGAD00001006418, EGAD00001004220, EGAD00001004448, EGAD00001004505, EGAD00001004533, EGAD00001004557, EGAD00001004393. The EGAD for the CBRL data is pending).
