# Supplementary figures and images for: The Extent and Impact of Variation in ADME Genes in Sub-Saharan African Populations
Source: Front Pharmacol. 2021 Apr 28;12:634016. doi: 10.3389/fphar.2021.634016 (PMC8549571; doi:10.3389/fphar.2021.634016)

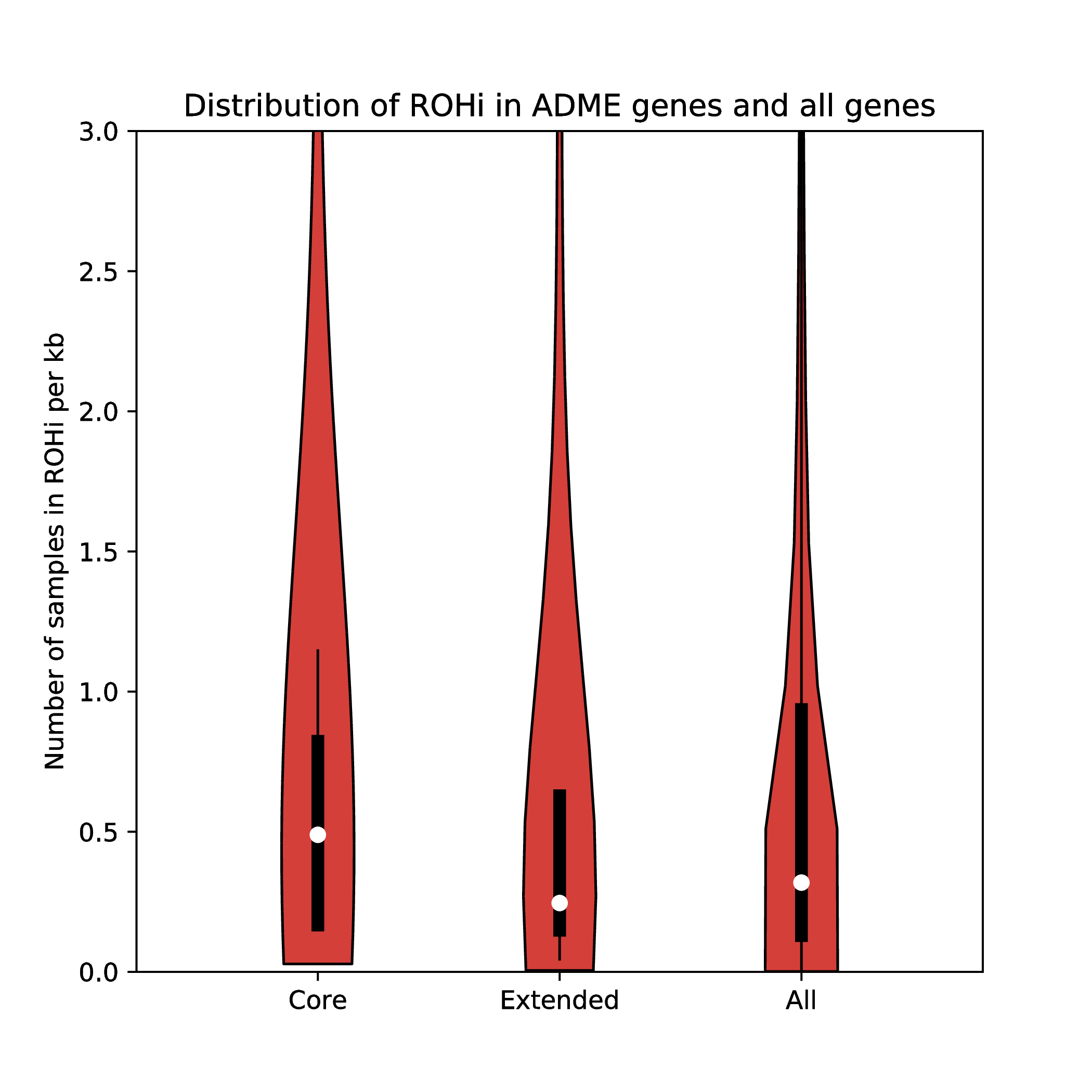

Supplement: Supplementary file 1 [file image6.tif]

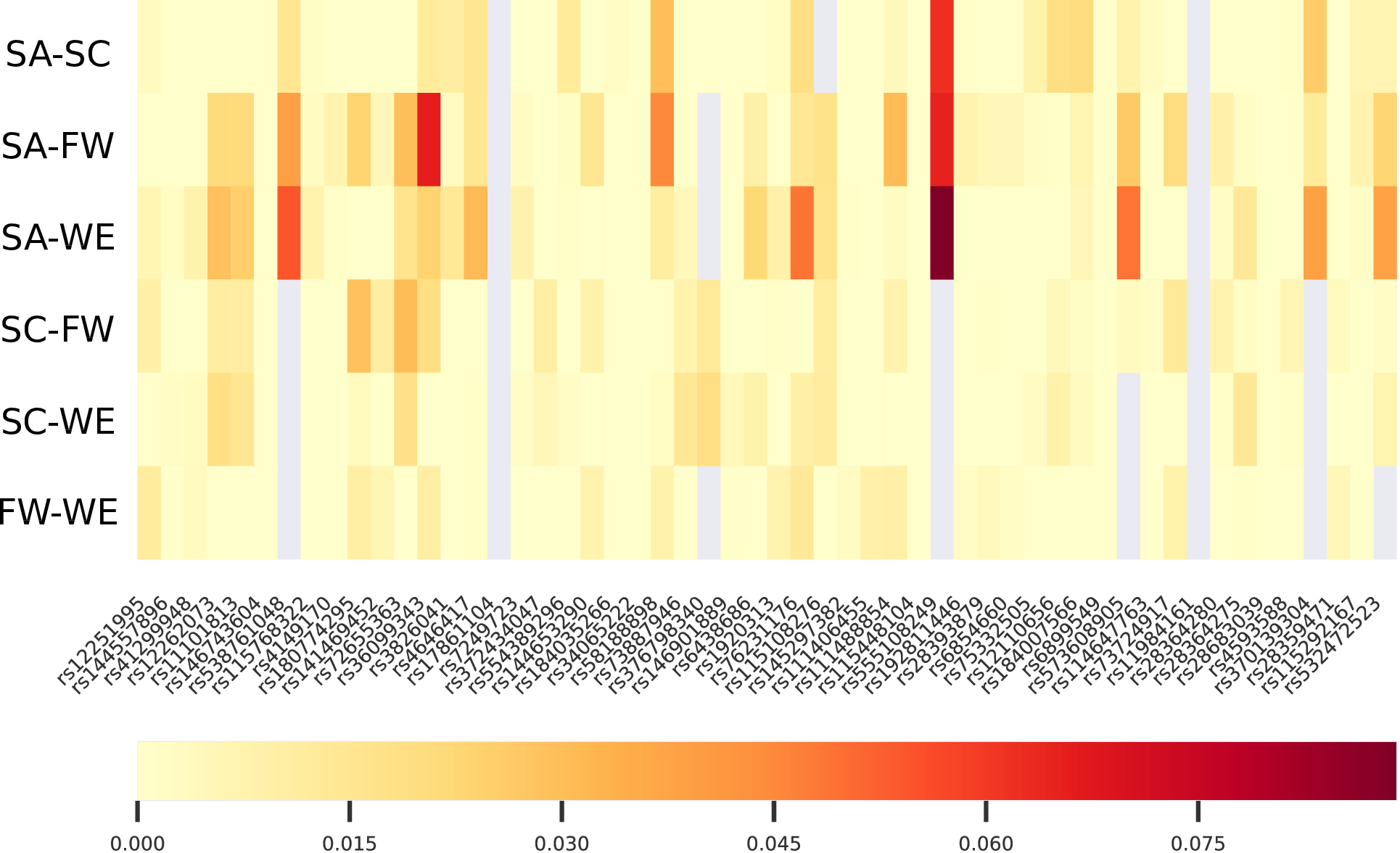

Supplement: Supplementary file 3 [file image4.tif]

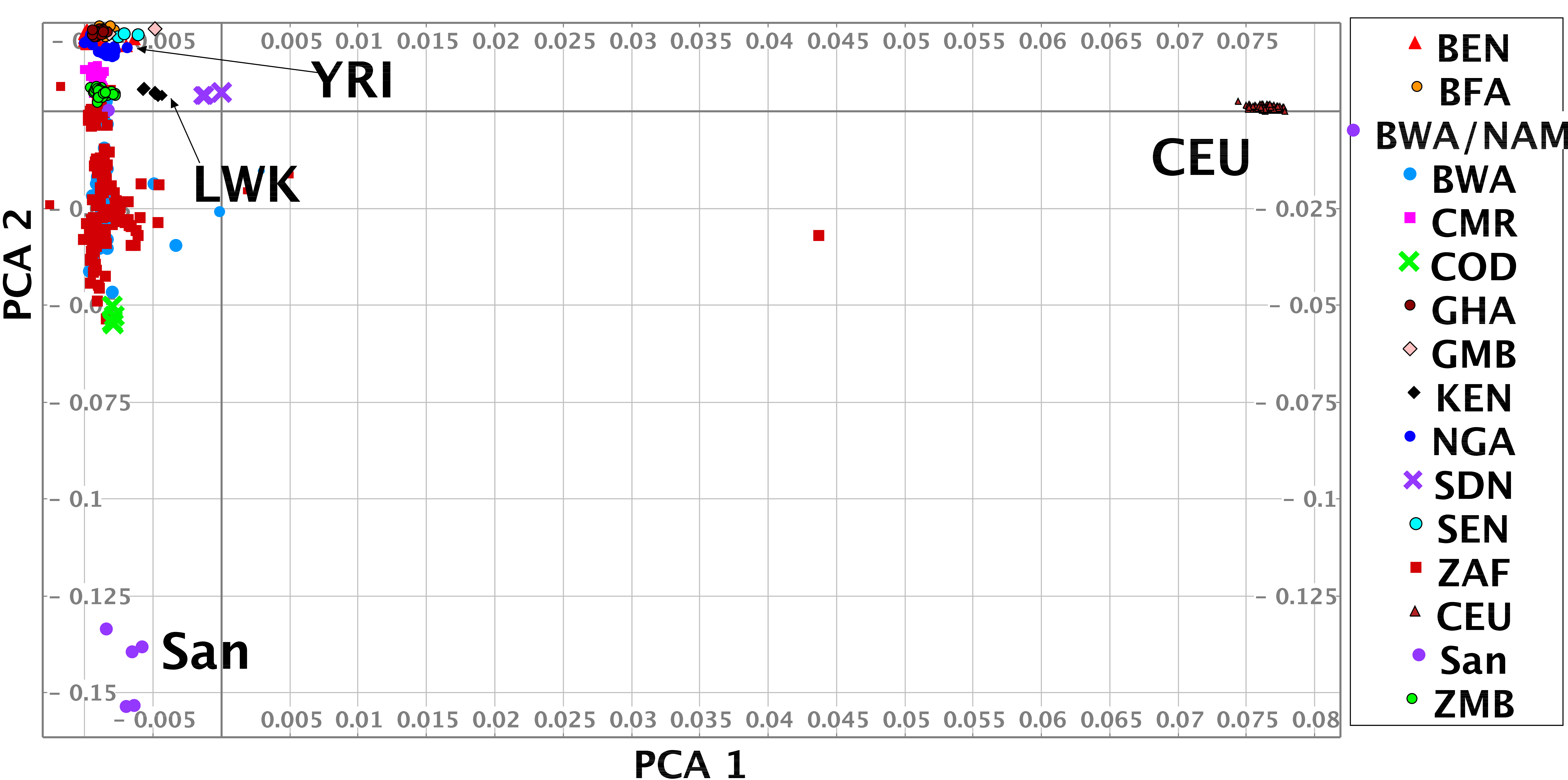

Supplement: Supplementary file 4 [file image2.tif]

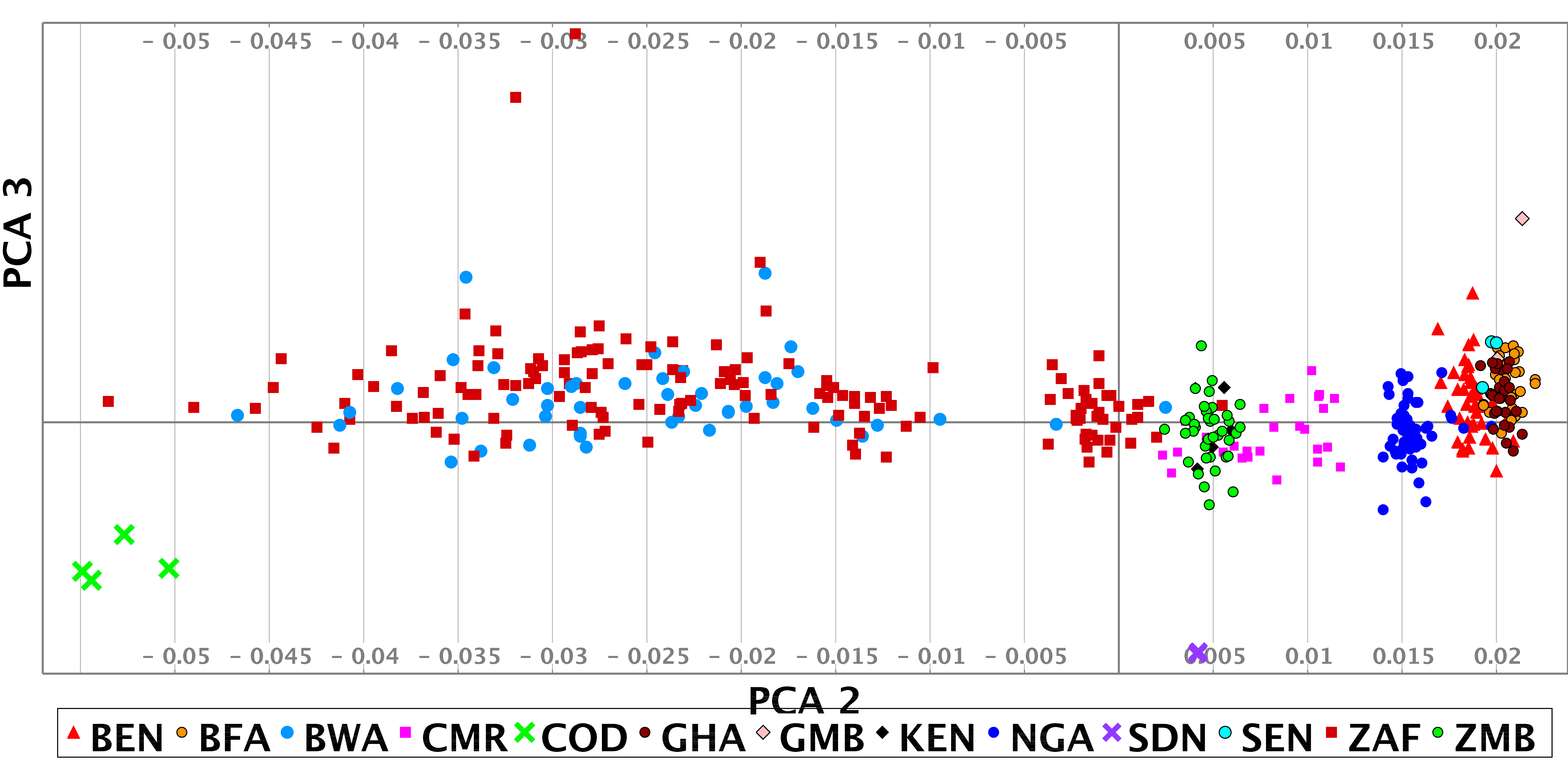

Supplement: Supplementary file 5 [file image1.tif]

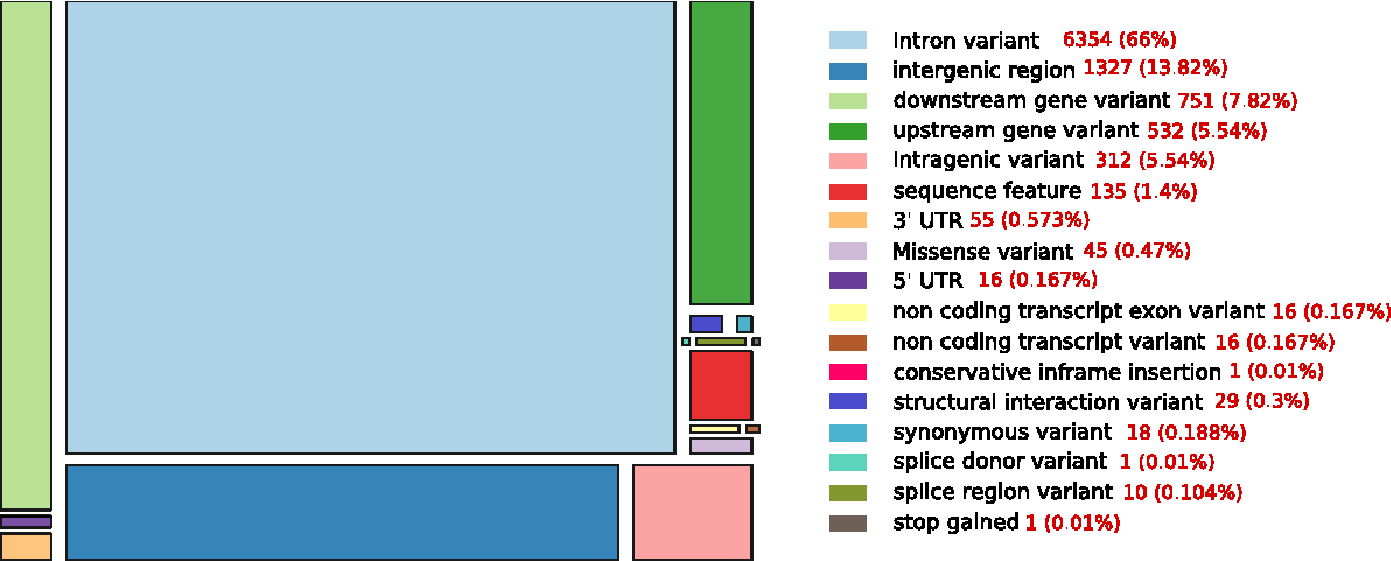

Supplement: Supplementary file 7 [file image5.tif]
