# Supplementary material for: The Extent and Impact of Variation in ADME Genes in Sub-Saharan African Populations
Source: Front Pharmacol. 2021 Apr 28;12:634016. doi: 10.3389/fphar.2021.634016 (PMC8549571; doi:10.3389/fphar.2021.634016)
Supplement: Supplementary file 6 [file datasheet1.pdf]

Supplementary Material: The extent and impact of variation in ADME genes in sub-Saharan African populations

Jorge da Rocha[1,2,‡], Houcemeddine Othman[1,‡], Gerrit Botha[3,‡], Laura Cottino[1,2,‡], David Twesigomwe[1,2,‡], Samah Ahmed[4,‡], Britt I. Drögemöller[5], Faisal M. Fadlelmola[4,‡], Philip Machanick[6,‡], Mamana Mbiyavanga[3,‡], Sumir Panji[3,‡], Galen E.B. Wright[7,8], Clement Adebamowo[9,10,‡], Mogomotsi Matshaba[11,12,‡], Gustave Simo[13,‡], Michèle Ramsay[1,2,‡], Martin C. Simuunza[14,‡], Caroline T. Tiemessen[15], Sandra Baldwin[16], Mathias Chiano[17], Charles Cox[17], Annette S. Gross[18], Pamela Thomas[19], Francisco-Javier Gamo[20] and Scott Hazelhurst[21,1,‡,*] as members of the H3Africa Consortium[‡].

1 Sydney Brenner Institute for Molecular Bioscience, Faculty of Health Sciences, University of the Witwatersrand, Johannesburg, South Africa. **2** Division of Human Genetics, National Health Laboratory Service, and School of Pathology, Faculty of Health Sciences, University of the Witwatersrand, Johannesburg, South Africa. **3** Computational Biology Division and H3ABioNet, Department of Integrative Biomedical Sciences, University of Cape Town, South Africa. **4** Centre for Bioinformatics and Systems Biology, Faculty of Science, University of Khartoum, Sudan. **5** Department of Biochemistry & Medical Genetics, University of Manitoba **6** Department of Computer Science, Rhodes University, Makhanda, South Africa. **7** Neuroscience Research Program, Kleysen Institute for Advanced Medicine, Winnipeg Health Sciences Centre and Max Rady College of Medicine, University of Manitoba. **8** Department of Pharmacology and Therapeutics, Rady Faculty of Health Sciences, University of Manitoba, Winnipeg, Manitoba, Canada. **9** Institute for Human Virology, Abuja, Nigeria. **10** Institute of Human Virology and Greenebaum Comprehensive Cancer Center, University of Maryland School of Medicine, Baltimore, MD **11** Botswana-Baylor Children's Clinical Center of Excellence, Gaborone, Botswana. **12** Baylor College of Medicine, Houston, United States. **13** Molecular Parasitology and Entomology Unit, Department of Biochemistry, Faculty of Science, University of Dschang, Dschang, Cameroon. **14** Department of Disease Control, School of Veterinary Medicine, University of Zambia, Lusaka, Zambia. **15** Centre for HIV and STIs, National Institute for Communicable Diseases, National Health Laboratory Services and Faculty of Health Sciences, University of the Witwatersrand, Johannesburg South Africa. **16** Drug Metabolism & Pharmacokinetics, GlaxoSmithKline R&D, Ware, UK. **19** Data and Computational Sciences, GlaxoSmithKline R&D, Stevenage, UK. **17** Human Genetics, GlaxoSmithKline R&D, Stevenage, UK. **18** Clinical Pharmacology Modelling & Simulation, GlaxoSmithKline R&D, Sydney, Australia. **20** Global Health, GlaxoSmithKline R&D, Madrid, Spain. **21** School of Electrical & Information Engineering, University of the Witwatersrand, Johannesburg, South Africa. ‡Members of the Human Heredity and Health in Africa Consortium. Authors not marked with ‡ are members of the H3A/GSK ADME Collaboration but not of the H3Africa Consortium.

S1 Supplementary Methods

Table S1 shows the individual steps involved in creating the final joint called VCF of 966 samples.

Table S1: Processing done on individual samples and jointly. Tool versions are included.

	BAM creation	GVCF creation	CombineGVCF	Genome calling
Tiemessen Lab	bwa-0.7.17, samtools-1.9, GATK v4.0.8.1			
AWI-Gen	bwa-0.7.17, samtools-1.9, GATK v4.0.8.1			
H3Africa Consortium	bwa-0.7.10, samtools/0.1.19/ picard-tools-1.119, GATK v3.3-0	GATK v4.0.8.1	GATK v4.0.8.1	GATK v4.1.3.0
1000 genomes African	bwa-0.5.9, picard-tools-1.53, samtools-0.1.17, GATK v1.2-29			
SAHGP	bwa-0.7.10, samtools/0.1.19/ picard-tools-1.119, GATK v3.3-0			
SGDP	bwa-0.7.10			

S2 Population Structure

A proper analysis of population structure is beyond the scope of this paper (See [1]). However it is useful to understand the extent of the diversity of the samples. Figure 2 in the main text showed a PCA of our samples (PC1 versus PC2). In Figure S1 we show PC2 versus PC3 of the same data, and we also show our data in the context of other populations. As explained in the methods section, the PC analysis included a number of reference populations including some 1000 Genomes European, Asian and African to ensure the analysis was unbiased. However, for clarity we only display some of the populations.

Figure S2 shows the structure chart of the same data as that of Figure 2 in the main paper, for $k = 3,\ldots,8$. Admixture proportions were computed with ADMIXTURE [2] – 30 independent estimates were run for each value of k and the final result computed using CLUMPP [3]. For clarity we have omitted some of the smaller groups. BWA=samples from Botswana, ZAF=samples from South Africa, NGA=Berom from Nigeria, BEN=Benin, CMR=Cameroon, BFA=Burkina Faso, GHA=Ghana, CEU=Utah residents (CEPH) with Northern and Western European ancestry (KGP), San=(Khoe and San from HAAD)

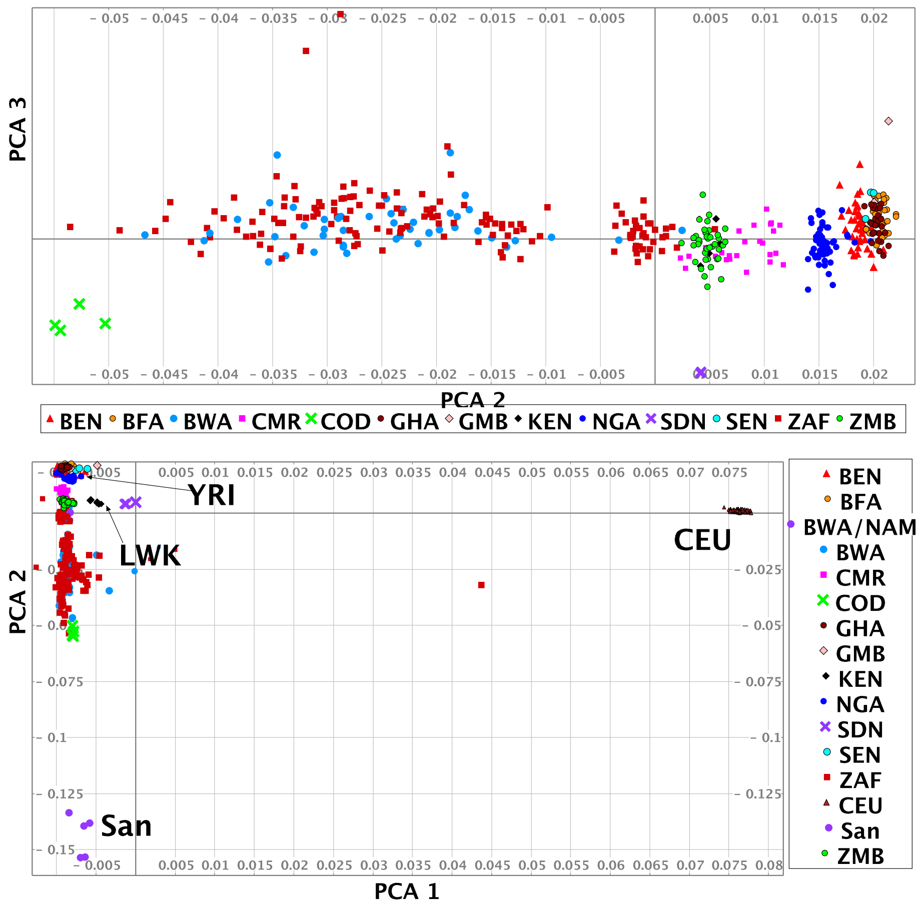

Figure S1: The figure on the top shows the PC2 versus PC3 of our data. The figure on the bottom shows PC1 versus PC2 – the same as Figure 2 – in the context of other African and world populations.

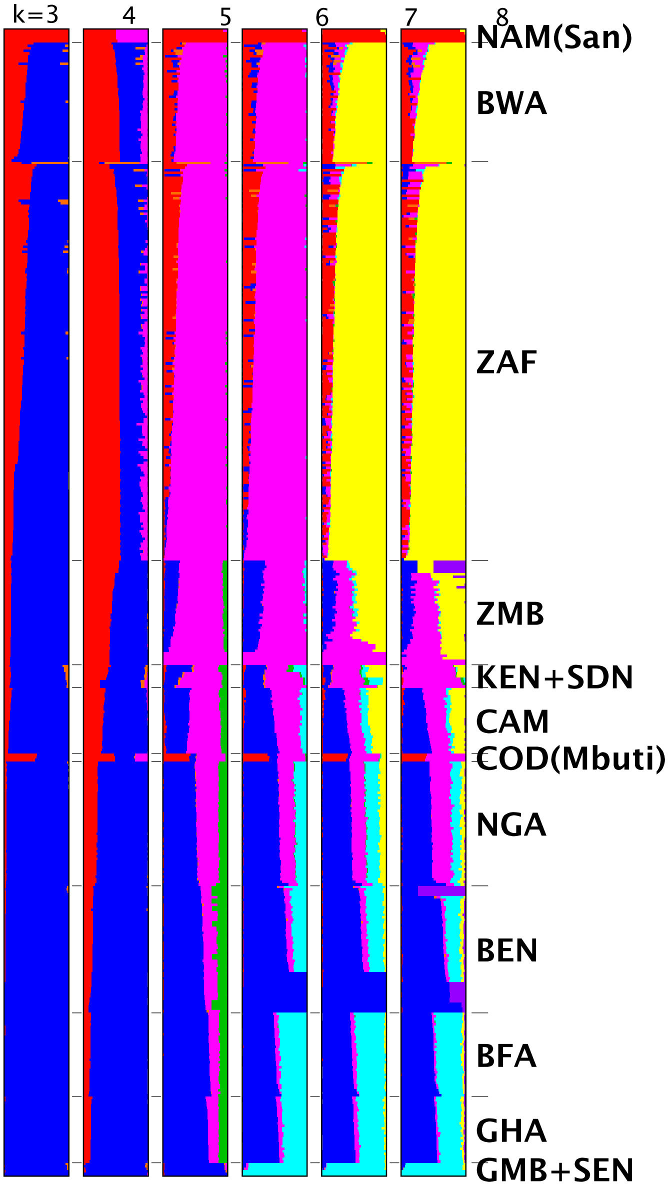

Figure S2: Structure chart – see text for description.

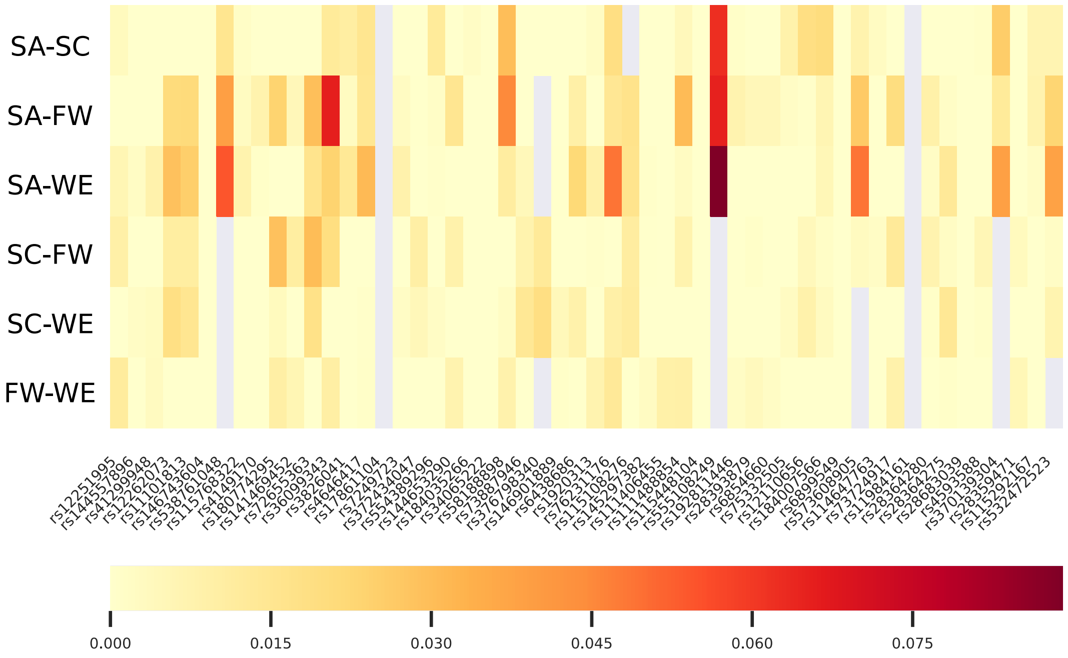

Figure S3: F_{ST} scores for regulatory SNPs. The maximum value is ≈ 0.09. Blanks are SNPs not scored between the given pairs. SA - Southern African; SC- South/Central African; KS - Khoe and San; FW - Far West African; WE - West African

S3 Regulatory Variation

Table S5 lists SNPs after filtering for:

- in any non-coding region
- MAF > 0.01
- CADD-PHRED score ≥ 10 [4]
- the canonical transcript for the SNP is not in a coding region (a check on the initial selection)
- binomial p-value compared with our entire 1000 Genomes data set < 0.05

The binomial p-value is calculated by taking the count of instances of the SNP as $2\times$homozygous count + heterozygous count; the total is this count for both alleles and the expected probability is the MAF for the entire 1000 Genomes data set. Give that our data is generally high coverage and some of the 1000 Genomes data is not, this is a first cut at finding significant regulatory SNPs.

Some instances where the "ancestral" allele have much lower MAF than the "minor" allele pointing to a need to review calling the ancestral allele. We excluded examples with this issue (none in any case passed the p-value threshold).

This initial filtering resulted in 54 SNPs. We then compared differences between pairs of population versus the overall population by using PLINK to calculate F_{ST} scores.

S4 Novel and Highly Differentiated Variants

Summary of novel variants identified within the HAAD dataset.

Table S2: Summary of known and novel variants called from the HAAD for the ADME core and extended genes.

HAAD ADME called datasets	Number of SNVs
Known ADME variants called	304,666
Novel ADME variants called	40,692
Core variants called – known	44,039
Core variants called – novel	5,818
Extended variants called – known	260,627
Extended variants called – novel	34,874

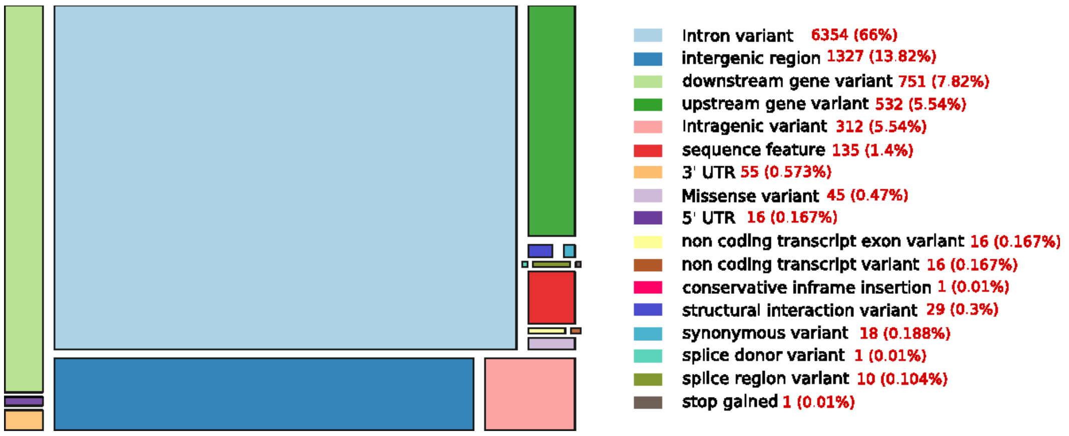

Figure S4: Distribution of ADME novel variant type by overall count.

Table S3: Summary of known and novel variants called from the HAAD set for the ADME core and extended genes. Syn=Synonymous mutations; NS=non-synonymous mutations, BW/Nam = Sample from Botswana or Namibia

	Categories of novel findings								
	**variants**	**Rare**	**Indels**	**Single-tons**	**NS**	**Syn**	**Stop gained**	**Stop lost**	**LOF**
Algeria	726	656	589	1002	1	0	0	0	0
Benin	4991	4074	2755	3260	31	13	1	0	0
Botswana	11274	8743	3492	8295	71	42	3	0	5
BW/Nam	1952	1364	1291	2090	4	2	0	0	0
Burkina Faso	5055	2343	2639	3807	26	19	1	0	1
Cameroon	4448	2358	2627	3421	31	15	2	0	2
Congo	2506	1363	1411	2336	12	5	0	0	1
Gambia	1110	1010	937	1390	0	1	0	0	0
Ghana	4527	2355	2585	3370	23	12	1	0	1
Kenya	2124	1496	1455	1896	9	2	0	0	0
Namibia	3591	1720	1864	3423	8	6	0	0	0
Nigeria	6916	6068	2927	5290	58	20	2	0	3
Senegal	1556	1268	1185	1543	5	1	0	0	0
South Africa	20492	18139	4523	13312	197	124	7	1	9
Sudan	1883	1181	1157	1883	4	6	0	0	0
Zambia	5296	2358	2768	3583	31	21	1	0	4

Table S4: Highly differentiated ADME variants in core genes in HAAD set. The frequency in different data sets is shown. Eff=Effect Effects: D=downstream; I=intron; M=missense; SR=splice region; SY=synonymous substitution; U=upstream

**ID**	**Gene**	**Eff**	**AC**	**HAAD**	**KG**	**KG Afr**	**ExAC**	**ExAC/Afr**	**gnomAD**	**gnomAD Afr**	**TOPMED**
rs113351578	GSTP1	SY	31	0.0338	0.0	0.0	8.14e-06	0.0	3.23e-05	0.0	0.0
rs112867476	GSTT1	M	21	0.031	0.0	0.0	2.04e-05	8.94e-05	0.0	0.0	0.0
rs1288697427	SULT1A1	SR	11	0.012	0.0	0.0	7.92e-06	0.0	9.86e-05	0.000347	0.0
rs187958013	GSTT1	SR	23	0.0339	0.0002	0.0008	2.67e-05	0.000268	0.0	0.0	0.0
rs1298375727	ABCC2	I	10	0.0109	0.0	0.0	0.0	0.0	3.48e-05	0.0	0.0
rs1303729583	ABCB1	U	10	0.0109	0.0	0.0	0.0	0.0	3.24e-05	0.000115	0.0
rs1468278213	CYP2A6	U	10	0.0109	0.0	0.0	0.0	0.0	3.33e-05	0.000118	0.0
rs111959737	CYP2B6	U	13	0.0142	0.0	0.0	0.0	0.0	6.47e-05	0.00023	0.0
rs111386238	CYP2B6	U	13	0.0142	0.0	0.0	0.0	0.0	6.46e-05	0.00023	0.0
rs112856484	CYP2B6	D	12	0.0131	0.0	0.0	0.0	0.0	6.46e-05	0.000229	0.0
rs368424346	GSTT1	SR	13	0.0193	0.0	0.0	5.6e-05	0.000951	0.000213	0.000806	0.0
rs376178963	GSTT1	U	13	0.0193	0.0	0.0	5.87e-05	0.000965	0.0003	0.00113	0.0
10:101597866/CT	ABCC2	I	9	0.0107	0.0	0.0	0.0	0.0	0.000123	0.0	0.0
rs990846266	ABCC2	I	10	0.0109	0.0	0.0	0.0	0.0	0.000176	0.0	0.0
rs1755884	SLC22A2	I	593	0.353	0.0138	0.0015	0.0	0.0	0.0394	0.00866	0.0696
rs3017670	SLC22A6	D	891	0.0273	0.346	0.473	0.329	0.45	0.379	0.45	0.126

Table S5: Significant regulatory SNPs. Variations are labeled as the genomic coordinate, ancestral allele (A2), minor allele (A1). MAF for "All" is from 1000 Genomes; for African from our data as are homozygous and heterozygous counts; "missing" means there was no count for the allele in the at number of experiments. The p-value is the binomial p-value for our African MAF using the "All" fraction as the expected probability. Region: 3=3'UTR; 5=5'UTR; D=Downstream; NCE=non-coding exon; U=Upstream. A1=homozygous in minor allele; A2=homozygous in ancestral allele; het=heterozygous.

			MAF		Counts				
Variation	Region	RS ID	All	African	A1	het	A2	missing	p-value
8_18066300_G_C	U	rs28359471	0.0825	0.3319	43	218	197	0	1.1507×10^{-101}
11_62752289_C_T	5	rs4149170	0.2101	0.4083	76	222	160	0	5.3011×10^{-42}
4_89082070_G_A	U	rs555108249	0.0004	0.0284	0	26	432	0	5.6599×10^{-39}
3_121659860_C_T	3	rs1920313	0.0329	0.1321	8	105	345	0	1.0383×10^{-37}
15_75018218_G_A	U	rs36099343	0.0114	0.0764	4	62	392	0	8.2029×10^{-35}
3_121609955_A_G	D	rs6438686	0.0453	0.1496	14	109	335	0	5.7812×10^{-34}
4_69398821_T_A	D	rs145297382	0.0543	0.1731	35	65	290	68	2.2176×10^{-32}
7_87132251_T_G	D	rs11984161	0.1088	0.2424	25	172	261	0	2.5778×10^{-30}
10_96617356_C_T	D	rs538761048	0.0002	0.0197	0	18	440	0	6.0177×10^{-30}
22_42528538_G_A	D	rs58188898	0.0238	0.0987	11	67	373	7	9.1907×10^{-29}
12_21392205_C_T	D	rs72655363	0.0062	0.0524	2	44	412	0	1.8785×10^{-28}
6_160541549_T_A	U	rs12110656	0.0319	0.1114	7	88	363	0	4.4545×10^{-27}
6_160539330_T_A	U	rs75332505	0.0321	0.1103	7	87	364	0	2.7043×10^{-26}
7_99280641_G_A	U	rs28683039	0.0313	0.1081	3	93	362	0	6.0056×10^{-26}
15_75018890_A_C	U	rs3826041	0.4255	0.5941	165	213	79	1	9.8293×10^{-25}
15_75018931_G_C	U	rs4646417	0.0363	0.1146	5	95	358	0	1.5339×10^{-24}
10_101611798_T_G	3	rs12251995	0.0236	0.0895	3	76	379	0	4.2030×10^{-24}
4_89083634_A_G	U	rs192811446	0.004	0.0393	0	36	422	0	8.7280×10^{-24}
4_89084896_G_T	NCE	rs6854660	0.004	0.0393	0	36	422	0	8.7280×10^{-24}
10_135355400_C_T	D	rs12262073	0.0421	0.1212	2	107	349	0	8.4664×10^{-23}
10_135355411_C_A	D	rs11101813	0.0421	0.1201	2	106	350	0	2.6654×10^{-22}
7_87133538_A_G	3	rs28364275	0.0218	0.0797	6	61	391	0	1.1276×10^{-20}
6_160542819_T_C	U	rs6899549	0.0192	0.0742	4	60	394	0	1.4139×10^{-20}
6_18126648_C_G	D	rs114647763	0.0268	0.0841	4	69	385	0	4.9648×10^{-18}
8_18247940_A_G	U	rs532472523	0.001	0.0186	0	17	441	0	2.3327×10^{-16}
3_121665481_C_T	D	rs76231176	0.0188	0.0611	3	50	405	0	4.7880×10^{-14}
4_69957763_C_T	U	rs111488854	0.007	0.0360	2	29	427	0	6.7940×10^{-14}
6_18124135_G_A	D	rs573608905	0.0012	0.0175	0	16	442	0	6.8944×10^{-14}
10_101612267_T_C	D	rs144557896	0.0066	0.0349	1	30	427	0	7.7669×10^{-14}
15_75018932_G_A	U	rs17861104	0.01	0.0426	0	39	419	0	1.3425×10^{-13}
11_67348925_A_T	U	rs180774295	0.0032	0.0229	0	21	437	0	6.5138×10^{-12}
22_42531473_A_G	D	rs376798340	0.0004	0.0110	1	8	446	3	7.7221×10^{-12}
8_18065932_G_A	U	rs370139304	0.0006	0.0120	0	11	447	0	1.9831×10^{-11}
19_41348142_T_A	D	rs372434047	0.0026	0.0197	0	18	440	0	8.8238×10^{-11}
8_18063651_T_G	U	rs4593588	0.0116	0.0371	1	32	425	0	6.8774×10^{-09}
3_121609253_G_A	D	rs146901889	0.0178	0.0459	1	40	417	0	5.4202×10^{-08}

10_96834043_C_T	U	rs115768322	0.004	0.0197	0	18	440	0	6.2949×10^{-08}
4_89007744_T_A	D	rs115448104	0.0104	0.0317	0	29	429	0	2.3959×10^{-07}
10_135354531_C_A	D	rs41299948	0.0136	0.0371	0	34	424	0	2.7682×10^{-07}
10_96616487_T_A	D	rs146743604	0.0084	0.0273	1	23	434	0	5.0394×10^{-07}
6_160541919_T_C	U	rs184007566	0.0042	0.0175	1	14	443	0	2.8005×10^{-06}
6_18156129_G_A	5	rs73724917	0.0028	0.0142	0	13	445	0	2.9653×10^{-06}
1_98386622_G_A	U	rs554389296	0.006	0.0208	0	19	438	1	4.6863×10^{-06}
22_42529156_C_T	D	rs73887946	0.0074	0.0200	1	16	434	7	1.9377×10^{-04}
22_24373140_G_T	D	rs184035266	0.0036	0.0147	3	4	333	118	2.2664×10^{-04}
19_41345991_G_A	D	rs7249723	0.0573	0.0841	7	63	388	0	6.2582×10^{-04}
7_87133243_C_T	3	rs28364280	0.0084	0.0197	1	16	441	0	9.8999×10^{-04}
4_89084520_G_T	U	rs28393879	0.0064	0.0164	0	15	443	0	1.0730×10^{-03}
2_234686908_A_G	D	rs34065222	0.01	0.0218	0	20	438	0	1.2168×10^{-03}
8_18084765_T_G	D	rs115292167	0.006	0.0131	0	12	446	0	1.0697×10^{-02}
4_69402508_A_G	D	rs111406455	0.0128	0.0230	3	12	376	67	1.3828×10^{-02}
1_98389412_T_A	U	rs144653290	0.0056	0.0120	0	11	447	0	1.5921×10^{-02}
3_121665494_T_G	D	rs115108276	0.0054	0.0109	0	10	448	0	2.9545×10^{-02}
12_21070798_G_A	D	rs141469452	0.007	0.0131	0	12	446	0	3.0569×10^{-02}

Some notes on most statistically-significant variants:

- rs28359471 (`http://grch37.ensembl.org/Homo_sapiens/Variation/Explore?db=core;r=8:18065800-18066800;v=rs28359471;vdb=variation;vf=477979426`) – NAT1 gene, includes two intronic transcripts and binding sites for two TFs: E2F1::ELK1 and ETV2::RFX5; gene expression correlations for:
  - Thyroid, Skin Sun Exposed Lower leg, Muscle Skeletal, Heart Atrial Appendage, Thyroid, Nerve Tibial, Artery Tibial
- rs4149170 (`http://grch37.ensembl.org/Homo_sapiens/Variation/Population?db=core;r=11:62751789-62752789;v=rs4149170;vdb=variation;vf=147699`) – SLC22A6 gene, includes three 5′ UTR and one NMD variant and binding sites for TFs: GCM1::CEBPB, GCM1::NHLH1, GCM1::SOX2, GCM2::PITX1, MAX, TFAP4::MAX, CLOCK::BHLHA15, SPDEF, GCM1::ETV7, GCM1::SPDEF, HOXB2::RFX5, TEAD4::CEBPD, TEAD4::RFX5, TFAP2C::MAX, E2F1::ELK1, ETV2::NHLH1, ETV2::ONECUT2, ERF::ONECUT2, FLI1::ONECUT2; gene expression correlations for:
  - Brain Hippocampus, Pituitary, Brain Cortex, Artery Aorta, Uterus, Artery Coronary, Brain Anterior cingulate cortex BA24, Brain Putamen basal ganglia
- rs555108249 (`http://grch37.ensembl.org/Homo_sapiens/Variation/Explore?db=core;r=4:89081570-89082570;v=rs555108249;vdb=variation;vf=517892906`) – ABCG2 gene, includes 2 intronic transcripts, no reported regulatory features
- rs1920313 (`http://grch37.ensembl.org/Homo_sapiens/Variation/Explore?db=core;r=3:121659360-121660360;v=rs1920313;vdb=variation;vf=323181359`) – ABCG2 gene, includes 2 3′ UTR variants; gene expression correlations for:
  - Whole Blood, Cells Transformed fibroblasts

- rs36099343 (`http://grch37.ensembl.org/Homo_sapiens/Variation/Explore?db=core;r=15:75017718-75018718;v=rs36099343;vdb=variation;vf=445364005`) – CYP1A1 gene, TF binding site ETV2::GSC2
  - Whole Blood

These examples indicate considerable regulatory variation in ADME SNPs and hence point to more complex analysis in future work.

S5 Regulatory variation

Genetic variants from ADME core genes were filtered for those meeting all the following criteria: in any non-coding region (10,000 bp up and downstream from canonical transcript); MAF >0.01; CADD-PHRED score $\geq$ 10 [4]; and binomial p-value compared with the entire 1000 Genomes Project data set < 0.05.

We compared these genetic variants (Table S5) for variability within pairs of populations as compared with the entire 1000 Genomes data set using F_{ST} scores [5]. Since the number of genetic variants is small, we do not stratify it further into specific regulatory elements.

There were 54 genetic variants across our African data sets in non-coding regions that have significantly higher prevalence than in the KGP overall data set (Table S5).

Figure S3 illustrates differences between population cluster pairs using F_{ST} scores. In most cases, the variability is not greater between pairs of population clusters (F_{ST} close to zero). We omit KS (Khoe and San) due to low sample size in this cluster.

S6 Runs of homozygosity

Runs of homozygosity (ROH) are areas in the genome where an individual has two identical copies of the genome due to shared ancestors on the maternal and paternal lines. The size of the ROH correlates with how recent the shared ancestor was. With high coverage data, we are able to detect ROHs of at least 300kb in size. High ROH is a measure of inbreeding decreased fitness and may be associated with ill health [6, 7]. However, ROH are not randomly distributed across the genome and *islands of homozygosity* (ROHi) are known to exist: regions where the ROH of several individuals within a population overlap [8]. There is some evidence that these islands are found as a result of positive selection.

There are a total of 634 ROH in the sample. The key metrics we use are the size of ROHi (that is, how many individuals are in the ROHi) and size normalised by size of gene (ROHi/kb). The genes which have largest ROHi and ROHi/kb are *CYP1A1*, *CYP1A2*. The *ABCB1* and *DPYD* genes are relatively large genes and have a large ROHi. Tables S6 and S7 show a summary of the ROH found in the core and extended genes in our data sets. The range of ROHi/kb varies significantly across all genes in the genome. Figure S5 shows a violin plot of the range of ROHi/kb in the core, extended, and all other genes in the genome. Statistical comparison is difficult because ranges are not normally distributed and a small number of extreme values skew the averages.

Figure S5 shows the distribution of runs of homozygosity across the genes, showing the density of ROHi per gene, normalised by gene length

Figure S5: Distribution of the ROHi/kb across the core, extended, and all genes. As there are extreme values, a y-cut-off of 3 was chosen to assist comparison. The median value and inter-quartile range is shown.

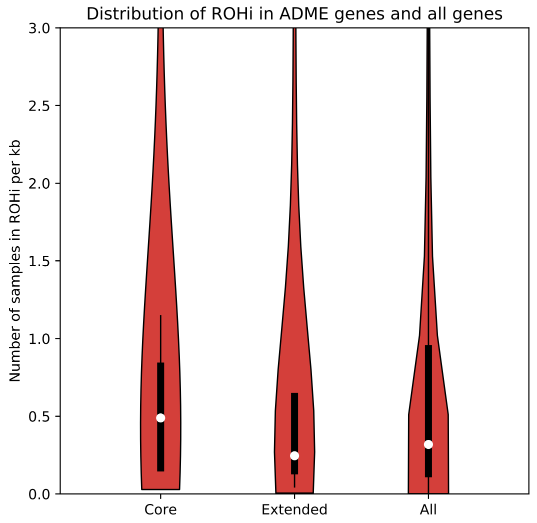

Regions of homozygosity in core and extended gene sets were identified with using PLINK [9], using settings consistent for high-coverage data [6], viz. :–homozyg-snp 30:, :homozyg-kb 300:, :–homozyg-window-snp: :30:, :–homozyg group-verbose:.

Table S6 shows the ROHi found in the core genes. For each group, the proportion of the individuals that are part of that ROHi for that gene is shown. In the two rightmost columns, the total number of individuals in the data set that are part of the ROHi is shown and then that number normalised by the length of the gene (i.e., #ROHi per thousand base pairs). In Table S7 a similar table is given for the extended data set.

Table S6: Runs of homozygosity in the core genes split by group. For each gene the number of ROH found across all samples is shown by group as a fraction of the individuals in that group who share the ROHi, followed the total number and the total normalised by gene length.

**Gene**	**SA**	**SC**	**KS**	**FW**	**WE**	**O**	# **ROHi**	**ROHi/kb**
ABCB1	0.33	0.27	0.33	0.25	0.41	0.00	98	0.47
ABCC2	0.04	0.03	0.00	0.00	0.04	0.00	10	0.14
ABCG2	0.02	0.02	0.00	0.00	0.01	0.00	4	0.03
CYP1A1	0.17	0.30	0.00	0.00	0.24	0.50	64	10.68
CYP1A2	0.12	0.30	0.00	0.00	0.12	0.50	46	5.93
CYP2A6	0.00	0.00	0.00	0.00	0.00	0.50	1	0.14
CYP2B6	0.00	0.00	0.00	0.00	0.00	0.50	1	0.04
CYP2C19	0.17	0.10	0.00	0.25	0.16	0.00	43	0.48
CYP2C8	0.08	0.07	0.00	0.00	0.08	0.00	21	0.64
CYP2C9	0.10	0.10	0.00	0.25	0.12	0.00	31	0.61
CYP2D6	0.04	0.03	0.00	0.00	0.04	0.00	11	2.51
CYP2E1	0.01	0.00	0.00	0.00	0.00	0.00	1	0.09
CYP3A4	0.09	0.10	0.33	0.00	0.06	0.00	23	0.84
CYP3A5	0.09	0.10	0.33	0.25	0.09	0.00	27	0.85
DPYD	0.15	0.28	0.00	0.50	0.38	0.00	78	0.09
DPYD-AS1	0.01	0.03	0.00	0.00	0.03	0.00	6	0.03
DPYD-AS2	0.13	0.27	0.00	0.50	0.32	0.00	68	60.12
GSTM1	0.00	0.05	0.00	0.00	0.01	0.00	4	0.67
GSTP1	0.06	0.02	0.00	0.25	0.05	0.00	14	4.58
GSTT1	0.03	0.03	0.00	0.00	0.02	0.00	7	0.86
NAT1	0.03	0.08	0.33	0.00	0.00	0.00	9	0.17
NAT2	0.03	0.05	0.00	0.00	0.00	0.00	6	0.60
SLC15A2	0.12	0.05	0.00	0.00	0.09	0.00	25	0.50
SLC22A1	0.01	0.02	0.00	0.00	0.02	0.00	4	0.11
SLC22A2	0.00	0.02	0.00	0.00	0.02	0.00	3	0.07
SLC22A6	0.07	0.00	0.00	0.00	0.03	0.00	10	1.19
SLCO1B1	0.07	0.08	0.00	0.00	0.05	0.00	18	0.17
SLCO1B3	0.06	0.10	0.00	0.00	0.05	0.00	18	0.17
SULT1A1	0.00	0.00	0.00	0.00	0.00	0.50	1	0.06
TPMT	0.02	0.03	0.00	0.00	0.02	0.00	6	0.22
UGT1A1	0.03	0.00	0.00	0.00	0.05	0.00	9	0.69
UGT2B15	0.03	0.13	0.33	0.00	0.03	0.00	15	0.62
UGT2B17	0.02	0.07	0.33	0.00	0.02	0.00	9	0.29
UGT2B7	0.06	0.12	0.33	0.00	0.04	0.00	19	1.15

Table S7: Regions of homozygosity in the extended genes split by group. For each gene the number of ROH found across all samples is shown by group as a fraction of the individuals in that group who share the ROHi, followed the total number and the total normalised by gene length.

Gene	SA	SC	KS	FW	W	O	# ROHi	ROHi/kb
ABCA1	0.02	0.00	0.00	0.00	0.01	0.00	6	0.04
ABCA4	0.03	0.04	0.00	0.00	0.05	0.33	18	0.14
ABCB11	0.02	0.04	0.00	0.00	0.01	0.00	8	0.07
ABCB4	0.06	0.03	0.20	0.00	0.05	0.00	22	0.30
ABCB5	0.02	0.03	0.00	0.00	0.02	0.00	9	0.06
ABCB6	0.02	0.01	0.20	0.00	0.01	0.00	7	0.76
ABCB7	0.00	0.00	0.00	0.00	0.00	0.33	1	0.01
ABCB8	0.00	0.01	0.00	0.00	0.01	0.00	3	0.15
ABCC1	0.01	0.00	0.00	0.00	0.00	0.00	1	0.01
ABCC10	0.01	0.04	0.00	0.00	0.02	0.33	10	0.44
ABCC11	0.04	0.07	0.20	0.00	0.06	0.33	26	0.38
ABCC12	0.07	0.12	0.20	0.00	0.10	0.67	43	0.67
ABCC13	0.01	0.02	0.00	0.00	0.00	0.00	3	0.11
ABCC3	0.02	0.04	0.00	0.00	0.00	0.33	8	0.14
ABCC4	0.03	0.01	0.00	0.00	0.01	0.00	7	0.02
ABCC5	0.02	0.05	0.00	0.00	0.02	0.00	14	0.14
ABCC5-AS1	0.02	0.05	0.00	0.00	0.02	0.00	13	2.56
ABCC6	0.00	0.00	0.00	0.00	0.00	0.33	1	0.01
ABCC8	0.05	0.01	0.00	0.00	0.06	0.00	18	0.21
ABCC9	0.06	0.05	0.00	0.00	0.04	0.00	22	0.16
ABCG1	0.01	0.01	0.00	0.00	0.00	0.00	3	0.03
ADH1A	0.01	0.04	0.20	0.00	0.06	0.00	18	1.23
ADH1B	0.02	0.05	0.20	0.00	0.06	0.00	20	1.33
ADH1C	0.01	0.05	0.20	0.00	0.06	0.00	18	1.09
ADH4	0.01	0.05	0.20	0.00	0.07	0.00	20	0.97
ADH5	0.01	0.04	0.20	0.00	0.07	0.00	19	1.07
ADH6	0.01	0.04	0.20	0.00	0.07	0.00	19	1.14
ADH7	0.01	0.04	0.00	0.00	0.05	0.00	14	0.60
ADHFE1	0.02	0.02	0.00	0.14	0.03	0.33	12	0.33
AHR	0.01	0.00	0.00	0.00	0.01	0.00	3	0.06
ALDH1A1	0.03	0.07	0.00	0.29	0.06	0.00	25	0.47
ALDH1A2	0.01	0.00	0.00	0.00	0.00	0.00	1	0.01
ALDH1A3	0.01	0.01	0.00	0.00	0.01	0.00	4	0.11
ALDH1B1	0.00	0.00	0.00	0.00	0.00	0.33	1	0.17
ALDH2	0.06	0.07	0.00	0.14	0.07	0.67	32	0.74
ALDH3A1	0.02	0.09	0.00	0.14	0.03	0.00	19	1.82
ALDH3A2	0.01	0.08	0.00	0.14	0.02	0.00	16	0.55
ALDH3B1	0.01	0.00	0.00	0.00	0.00	0.00	2	0.10
ALDH3B2	0.02	0.01	0.00	0.00	0.01	0.00	6	0.31
ALDH4A1	0.01	0.02	0.00	0.00	0.01	0.00	5	0.16
ALDH5A1	0.02	0.03	0.00	0.00	0.01	0.00	7	0.17

Gene	SA	SC	KS	FW	W	O	# ROHi	ROHi/kb
ALDH6A1	0.02	0.01	0.00	0.00	0.02	0.00	8	0.30
ALDH7A1	0.02	0.00	0.00	0.00	0.01	0.00	5	0.09
ALDH8A1	0.00	0.02	0.00	0.00	0.01	0.00	4	0.12
ALDH9A1	0.03	0.02	0.00	0.00	0.02	0.00	11	0.30
AOX1	0.06	0.01	0.00	0.00	0.01	0.00	11	0.13
ARNT	0.05	0.07	0.20	0.00	0.04	0.00	23	0.34
ARSA	0.01	0.02	0.00	0.00	0.00	0.00	4	0.74
ATP7A	0.00	0.00	0.00	0.00	0.00	0.33	1	0.01
ATP7B	0.09	0.13	0.00	0.00	0.12	0.00	48	0.61
CAT	0.01	0.01	0.00	0.00	0.01	0.00	3	0.09
CBR1	0.01	0.00	0.00	0.00	0.02	0.00	4	1.23
CBR3	0.01	0.01	0.00	0.00	0.01	0.00	3	0.26
CBR3-AS1	0.01	0.01	0.00	0.00	0.01	0.00	3	0.12
CDA	0.01	0.00	0.00	0.00	0.00	0.00	2	0.07
CES1	0.00	0.01	0.00	0.00	0.00	0.00	1	0.03
CES2	0.07	0.11	0.00	0.00	0.10	0.33	40	3.76
CFTR	0.06	0.06	0.00	0.14	0.13	0.00	39	0.21
CHST1	0.02	0.03	0.00	0.14	0.01	0.00	9	0.50
CHST10	0.01	0.04	0.20	0.00	0.05	0.00	15	0.58
CHST11	0.02	0.07	0.00	0.00	0.01	0.00	13	0.04
CHST12	0.01	0.01	0.00	0.00	0.00	0.00	3	0.10
CHST13	0.01	0.02	0.20	0.00	0.02	0.33	10	0.53
CHST2	0.01	0.01	0.00	0.00	0.00	0.00	3	0.71
CHST3	0.02	0.04	0.00	0.00	0.02	0.00	10	0.20
CHST4	0.03	0.07	0.20	0.14	0.10	0.00	31	2.49
CHST5	0.02	0.06	0.20	0.14	0.04	0.00	19	2.86
CHST6	0.02	0.08	0.20	0.14	0.03	0.00	20	0.91
CHST7	0.00	0.00	0.00	0.00	0.00	0.33	1	0.04
CHST8	0.02	0.02	0.00	0.00	0.00	0.00	5	0.03
CHST9	0.02	0.04	0.20	0.00	0.01	0.00	9	0.03
CYB5R3	0.01	0.00	0.00	0.00	0.00	0.00	2	0.06
CYP11A1	0.03	0.03	0.00	0.00	0.05	0.00	16	0.53
CYP11B1	0.01	0.00	0.00	0.00	0.01	0.00	2	0.27
CYP11B2	0.01	0.00	0.00	0.00	0.01	0.00	2	0.27
CYP17A1	0.18	0.27	0.20	0.00	0.27	0.33	105	14.99
CYP19A1	0.03	0.04	0.00	0.00	0.07	0.00	22	0.17
CYP1B1	0.02	0.00	0.20	0.00	0.02	0.00	8	0.93
CYP1B1-AS1	0.01	0.00	0.20	0.00	0.04	0.00	9	0.18
CYP20A1	0.11	0.09	0.00	0.00	0.11	0.67	47	0.70
CYP21A2	0.07	0.06	0.00	0.14	0.02	0.00	23	6.86
CYP24A1	0.01	0.00	0.00	0.00	0.01	0.00	2	0.10
CYP26A1	0.02	0.03	0.00	0.00	0.02	0.00	10	2.27
CYP26C1	0.02	0.04	0.00	0.00	0.02	0.00	11	1.48
CYP27B1	0.04	0.06	0.00	0.00	0.02	0.00	17	3.50
CYP2A13	0.00	0.00	0.00	0.00	0.00	0.33	1	0.13

Gene	SA	SC	KS	FW	W	O	# ROHi	ROHi/kb
CYP2A7	0.00	0.00	0.00	0.00	0.00	0.33	1	0.14
CYP2C18	0.11	0.08	0.00	0.14	0.12	0.00	46	0.87
CYP2F1	0.00	0.00	0.00	0.00	0.00	0.33	1	0.07
CYP2J2	0.03	0.04	0.00	0.00	0.01	0.00	11	0.33
CYP2R1	0.11	0.10	0.00	0.14	0.09	0.00	43	3.03
CYP2S1	0.01	0.00	0.00	0.00	0.00	0.00	1	0.07
CYP39A1	0.03	0.04	0.00	0.00	0.03	0.33	15	0.15
CYP3A43	0.07	0.04	0.00	0.00	0.03	0.00	21	0.55
CYP3A7	0.06	0.05	0.20	0.14	0.06	0.00	27	0.90
CYP3A7-CYP3AP1	0.06	0.05	0.20	0.14	0.06	0.00	27	0.53
CYP46A1	0.02	0.01	0.00	0.00	0.00	0.00	4	0.09
CYP4A11	0.00	0.00	0.00	0.00	0.00	0.33	1	0.08
CYP4B1	0.00	0.00	0.00	0.00	0.00	0.33	1	0.05
CYP4F11	0.01	0.00	0.00	0.14	0.01	0.00	4	0.18
CYP4F12	0.01	0.00	0.00	0.14	0.01	0.00	5	0.21
CYP4F2	0.01	0.00	0.00	0.14	0.01	0.00	4	0.20
CYP4F3	0.01	0.00	0.00	0.14	0.01	0.00	5	0.25
CYP4F8	0.01	0.00	0.00	0.00	0.01	0.00	4	0.28
CYP4Z1	0.01	0.00	0.00	0.00	0.00	0.00	2	0.04
CYP51A1	0.09	0.09	0.00	0.29	0.09	0.33	42	1.86
CYP7A1	0.01	0.01	0.00	0.00	0.02	0.00	7	0.70
CYP7B1	0.02	0.06	0.00	0.00	0.07	0.00	22	0.11
CYP8B1	0.01	0.06	0.00	0.00	0.04	0.00	15	3.80
DDO	0.01	0.03	0.00	0.00	0.01	0.00	7	0.30
DHRS1	0.01	0.02	0.00	0.00	0.01	0.00	4	0.43
DHRS12	0.04	0.06	0.00	0.00	0.05	0.00	21	0.58
DHRS13	0.05	0.09	0.00	0.00	0.03	0.00	23	4.35
DHRS2	0.01	0.02	0.00	0.00	0.00	0.00	4	0.43
DHRS3	0.01	0.02	0.20	0.00	0.02	0.00	9	0.18
DHRS4	0.00	0.00	0.00	0.00	0.00	0.33	1	0.06
DHRS4-AS1	0.00	0.00	0.00	0.00	0.00	0.33	1	0.06
DHRS4L1	0.01	0.02	0.00	0.00	0.01	0.00	4	0.09
DHRS4L2	0.01	0.02	0.00	0.00	0.01	0.00	4	0.11
DHRS7	0.08	0.12	0.20	0.14	0.11	0.00	46	2.22
DHRS7B	0.01	0.03	0.00	0.00	0.00	0.00	4	0.06
DHRS7C	0.02	0.00	0.00	0.00	0.00	0.00	4	0.20
DHRS9	0.01	0.03	0.00	0.00	0.01	0.00	6	0.21
DHRSX	0.00	0.00	0.00	0.00	0.00	1.00	3	0.01
DPEP1	0.01	0.02	0.20	0.00	0.01	0.00	6	0.24
EPHX1	0.02	0.01	0.20	0.00	0.02	0.00	8	0.23
EPHX2	0.03	0.00	0.00	0.00	0.01	0.00	7	0.13
FMO1	0.02	0.00	0.00	0.00	0.02	0.00	6	0.16
FMO2	0.02	0.00	0.00	0.00	0.02	0.00	6	0.22
FMO3	0.02	0.00	0.00	0.00	0.02	0.00	6	0.22
FMO4	0.01	0.00	0.00	0.00	0.01	0.00	4	0.14

Gene	SA	SC	KS	FW	W	O	# ROHi	ROHi/kb
FMO5	0.04	0.02	0.00	0.00	0.04	0.00	14	0.34
FMO6P	0.02	0.00	0.00	0.00	0.02	0.00	6	0.25
GPX1	0.05	0.10	0.40	0.14	0.05	0.33	31	26.20
GPX2	0.04	0.02	0.00	0.00	0.02	0.00	13	3.46
GPX3	0.01	0.00	0.00	0.14	0.01	0.00	3	0.35
GPX4	0.00	0.00	0.20	0.00	0.00	0.00	1	0.35
GPX5	0.04	0.10	0.20	0.29	0.09	0.00	35	3.91
GPX6	0.04	0.10	0.20	0.29	0.09	0.00	36	2.88
GPX7	0.04	0.04	0.20	0.00	0.01	0.00	12	1.80
GSR	0.02	0.01	0.20	0.00	0.02	0.33	9	0.18
GSS	0.11	0.11	0.00	0.00	0.10	0.00	46	1.68
GSTA1	0.01	0.00	0.00	0.00	0.00	0.00	2	0.16
GSTA2	0.01	0.01	0.00	0.00	0.00	0.00	2	0.15
GSTA3	0.01	0.01	0.00	0.00	0.00	0.33	4	0.31
GSTA4	0.02	0.04	0.00	0.14	0.01	0.33	11	0.63
GSTA5	0.01	0.01	0.00	0.00	0.00	0.33	4	0.28
GSTCD	0.02	0.06	0.00	0.14	0.07	0.33	23	0.17
GSTK1	0.04	0.05	0.00	0.00	0.04	0.00	20	3.51
GSTM2	0.00	0.03	0.00	0.00	0.01	0.00	4	0.25
GSTM3	0.01	0.02	0.00	0.00	0.00	0.00	3	0.42
GSTM4	0.00	0.03	0.00	0.00	0.01	0.00	5	0.53
GSTM5	0.00	0.02	0.00	0.00	0.01	0.00	3	0.50
GSTO1	0.01	0.00	0.00	0.00	0.01	0.00	3	0.23
GSTO2	0.01	0.00	0.00	0.00	0.02	0.00	4	0.13
GSTT2	0.01	0.04	0.00	0.00	0.02	0.00	9	2.37
GSTZ1	0.02	0.01	0.00	0.14	0.01	0.00	8	0.75
HAGH	0.02	0.02	0.00	0.00	0.00	0.00	6	0.33
HNF4A	0.02	0.04	0.00	0.00	0.01	0.00	10	0.13
HNF4A-AS1	0.02	0.03	0.00	0.00	0.01	0.00	7	0.39
HNMT	0.01	0.06	0.00	0.00	0.01	0.00	10	0.19
HSD11B1	0.01	0.01	0.20	0.00	0.01	0.00	6	0.12
HSD17B11	0.04	0.03	0.00	0.00	0.01	0.00	12	0.22
HSD17B14	0.00	0.01	0.20	0.00	0.01	0.00	3	0.13
IAPP	0.02	0.02	0.00	0.00	0.01	0.00	8	1.12
KCNJ11	0.05	0.02	0.00	0.00	0.06	0.00	19	4.65
MAT1A	0.02	0.02	0.00	0.00	0.02	0.00	9	0.50
METAP1	0.01	0.04	0.20	0.00	0.06	0.00	17	0.25
MGST1	0.02	0.00	0.00	0.00	0.01	0.00	4	0.13
MGST2	0.01	0.01	0.20	0.00	0.01	0.00	4	0.05
MGST3	0.03	0.02	0.00	0.00	0.02	0.00	11	0.44
MPO	0.02	0.03	0.00	0.00	0.01	0.00	8	0.72
NNMT	0.01	0.01	0.00	0.00	0.01	0.00	4	0.24
NOS1	0.01	0.01	0.00	0.00	0.01	0.00	3	0.02
NOS2	0.01	0.03	0.00	0.00	0.03	0.00	9	0.21
NOS3	0.00	0.01	0.00	0.00	0.01	0.00	3	0.13

Gene	SA	SC	KS	FW	W	O	# ROHi	ROHi/kb
NR1I2	0.07	0.09	0.00	0.00	0.14	0.00	45	1.18
NR1I3	0.01	0.01	0.00	0.00	0.04	0.00	10	1.17
PDE3A	0.02	0.03	0.20	0.00	0.02	0.00	10	0.03
PDE3B	0.14	0.11	0.00	0.14	0.12	0.00	55	0.24
PLGLB1	0.00	0.00	0.00	0.00	0.00	0.67	2	0.18
PNMT	0.08	0.06	0.00	0.57	0.06	0.00	34	13.63
PON1	0.07	0.02	0.00	0.00	0.02	0.00	17	0.65
PON2	0.07	0.01	0.00	0.00	0.01	0.00	14	0.46
PON3	0.07	0.01	0.00	0.00	0.02	0.00	15	0.41
POR	0.01	0.02	0.00	0.00	0.01	0.00	4	0.06
PPARA	0.02	0.02	0.00	0.00	0.00	0.00	5	0.05
PPARD	0.06	0.05	0.20	0.00	0.07	0.00	27	0.32
PPARG	0.06	0.08	0.20	0.00	0.06	0.00	28	0.19
RXRA	0.00	0.00	0.00	0.00	0.00	0.33	1	0.01
SERPINA7	0.00	0.00	0.00	0.00	0.00	0.33	1	0.18
SLC10A1	0.02	0.01	0.00	0.00	0.02	0.00	7	0.33
SLC10A2	0.00	0.00	0.00	0.00	0.01	0.00	2	0.09
SLC13A1	0.06	0.05	0.20	0.00	0.06	0.00	26	0.30
SLC13A2	0.02	0.03	0.00	0.00	0.04	0.00	13	0.54
SLC13A3	0.01	0.00	0.20	0.00	0.00	0.00	2	0.02
SLC15A1	0.01	0.01	0.00	0.00	0.01	0.00	4	0.06
SLC16A1	0.02	0.05	0.00	0.00	0.03	0.00	14	0.31
SLC16A1-AS1	0.02	0.05	0.00	0.00	0.03	0.00	14	1.83
SLC19A1	0.01	0.01	0.00	0.00	0.01	0.00	5	0.18
SLC22A10	0.04	0.02	0.00	0.00	0.03	0.33	14	0.64
SLC22A11	0.04	0.01	0.00	0.00	0.02	0.00	11	0.69
SLC22A12	0.04	0.00	0.00	0.00	0.02	0.00	10	0.87
SLC22A13	0.04	0.04	0.00	0.00	0.06	0.00	21	1.68
SLC22A14	0.04	0.04	0.00	0.00	0.04	0.00	18	1.45
SLC22A15	0.04	0.05	0.20	0.00	0.04	0.33	21	0.22
SLC22A16	0.02	0.03	0.00	0.00	0.02	0.00	9	0.17
SLC22A17	0.02	0.01	0.00	0.00	0.00	0.00	5	0.76
SLC22A18	0.01	0.01	0.00	0.00	0.02	0.00	6	0.24
SLC22A18AS	0.01	0.01	0.00	0.00	0.02	0.00	6	0.38
SLC22A3	0.00	0.01	0.00	0.00	0.03	0.00	6	0.06
SLC22A4	0.04	0.00	0.00	0.00	0.01	0.00	8	0.16
SLC22A5	0.04	0.01	0.00	0.00	0.01	0.00	9	0.35
SLC22A7	0.02	0.04	0.00	0.00	0.02	0.33	12	1.65
SLC22A8	0.04	0.01	0.00	0.00	0.03	0.00	13	0.56
SLC22A9	0.03	0.01	0.00	0.00	0.03	0.33	12	0.30
SLC27A1	0.01	0.01	0.00	0.00	0.00	0.00	3	0.08
SLC28A1	0.02	0.01	0.20	0.00	0.02	0.00	9	0.15
SLC28A2	0.01	0.04	0.00	0.00	0.02	0.00	10	0.42
SLC28A3	0.01	0.01	0.40	0.00	0.01	0.00	7	0.08
SLC29A1	0.02	0.01	0.00	0.00	0.00	0.33	5	0.34

Gene	SA	SC	KS	FW	W	O	# ROHi	ROHi/kb
SLC29A2	0.04	0.04	0.00	0.14	0.02	0.33	17	1.83
SLC2A4	0.01	0.00	0.00	0.00	0.00	0.00	1	0.16
SLC2A5	0.01	0.01	0.00	0.00	0.01	0.00	3	0.09
SLC5A6	0.04	0.03	0.40	0.00	0.05	0.33	20	1.57
SLC6A6	0.00	0.02	0.00	0.00	0.00	0.00	2	0.02
SLC7A5	0.01	0.00	0.20	0.00	0.00	0.00	2	0.05
SLC7A7	0.01	0.01	0.00	0.00	0.00	0.00	3	0.06
SLC7A8	0.02	0.02	0.00	0.00	0.00	0.00	6	0.10
SLCO1A2	0.03	0.02	0.00	0.00	0.02	0.00	11	0.08
SLCO1C1	0.03	0.02	0.20	0.00	0.02	0.00	12	0.21
SLCO2A1	0.03	0.03	0.20	0.00	0.02	0.33	13	0.13
SLCO2B1	0.04	0.03	0.00	0.00	0.01	0.00	10	0.18
SLCO3A1	0.01	0.03	0.00	0.00	0.02	0.00	8	0.03
SLCO4A1	0.01	0.00	0.00	0.00	0.01	0.00	4	0.13
SLCO4C1	0.04	0.10	0.00	0.29	0.09	0.33	36	0.58
SLCO5A1	0.01	0.01	0.00	0.00	0.01	0.00	4	0.02
SLCO6A1	0.03	0.07	0.00	0.29	0.08	0.33	29	0.23
SLX1A-SULT1A3	0.01	0.04	0.00	0.00	0.01	0.33	8	0.81
SOD1	0.03	0.03	0.00	0.00	0.01	0.00	9	0.97
SOD2	0.02	0.02	0.00	0.00	0.01	0.00	6	0.42
SOD3	0.01	0.00	0.20	0.00	0.01	0.33	5	0.93
SULF1	0.02	0.00	0.00	0.00	0.01	0.00	5	0.03
SULT1A2	0.00	0.00	0.00	0.00	0.00	0.33	1	0.20
SULT1A3	0.01	0.04	0.00	0.00	0.01	0.33	8	1.57
SULT1B1	0.01	0.01	0.20	0.00	0.06	0.00	13	0.39
SULT1C2	0.04	0.01	0.20	0.14	0.03	0.00	14	0.66
SULT1E1	0.01	0.02	0.20	0.00	0.08	0.00	18	0.95
SULT2A1	0.00	0.02	0.20	0.00	0.00	0.00	3	0.19
SULT2B1	0.01	0.02	0.20	0.00	0.01	0.00	6	0.13
SULT4A1	0.02	0.04	0.20	0.14	0.06	0.00	20	0.53
TAP1	0.02	0.02	0.00	0.00	0.01	0.00	6	0.68
TAP2	0.02	0.03	0.00	0.00	0.01	0.00	8	0.47
UGT1A10	0.02	0.01	0.00	0.00	0.04	0.00	10	0.07
UGT1A3	0.02	0.00	0.00	0.00	0.04	0.00	9	0.20
UGT1A4	0.02	0.01	0.00	0.00	0.04	0.00	10	0.18
UGT1A5	0.02	0.01	0.00	0.00	0.04	0.00	10	0.17
UGT1A6	0.02	0.01	0.00	0.00	0.04	0.00	10	0.12
UGT1A7	0.02	0.01	0.00	0.00	0.04	0.00	10	0.11
UGT1A8	0.02	0.01	0.00	0.00	0.04	0.00	10	0.06
UGT1A9	0.02	0.01	0.00	0.00	0.04	0.00	10	0.10
UGT2A1	0.01	0.03	0.20	0.00	0.05	0.00	14	0.22
UGT2B10	0.04	0.17	0.40	0.00	0.10	0.00	45	2.81
UGT2B11	0.03	0.04	0.20	0.00	0.01	0.00	13	0.90
UGT2B28	0.04	0.01	0.20	0.00	0.02	0.00	11	0.76
UGT2B4	0.01	0.03	0.20	0.00	0.02	0.00	10	0.64

Gene	SA	SC	KS	FW	W	O	# ROHi	ROHi/kb
UGT8	0.05	0.04	0.00	0.00	0.06	0.00	22	0.28
XDH	0.06	0.05	0.20	0.00	0.06	0.33	27	0.34

S7 ADME High Impact variants HI-vars

The list of High Impact variants (HI-vars) identified in core and extended genes is displayed below. Variants are identified via their chromosomal location (GRCh37) and allele format, in order of ref allele then alternative allele (chr-pos-ref-alt). The dbSNP identifier is given for variants listed in dbSNP version 151. The allele frequency (AF) for the alternative allele is given for the combined joint called HAAD and KGA datasets (N=458 HAAD + N=506 KGA) in the following categories: Ultra-rare (<1%), Rare (1-5%), Common (5-20%) Very-common (>20%).

Table S8: ADME *Core* and *Extended* High Impact variants (HI-vars)

Gene	CHR	Variant	ID	AF
SLC2A5	1	1-9097751-G-A	rs368307795	Ultra-rare
SLC2A5	1	1-9098553-A-G	1-9098553-A-G	Ultra-rare
ALDH4A1	1	1-19199400-G-A	rs72953172	Rare
ALDH4A1	1	1-19203977-G-C	1-19203977-G-C	Ultra-rare
ALDH4A1	1	1-19208293-G-C	rs143835001	Ultra-rare
ALDH4A1	1	1-19209784-G-A	rs531481821	Ultra-rare
ALDH4A1	1	1-19211987-C-T	rs113846237	Rare
ALDH4A1	1	1-19212083-G-A	rs142923662	Ultra-rare
CDA	1	1-20915740-G-A	rs149993022	Ultra-rare
CDA	1	1-20931474-G-A	rs60369023	Ultra-rare
CYP4B1	1	1-47276622-G-T	rs116105604	Ultra-rare
CYP4B1	1	1-47279164-A-G	rs145508204	Ultra-rare
CYP4B1	1	1-47279699-T-C	rs151203772	Ultra-rare
CYP4B1	1	1-47279898-C-T	rs45446505	Rare
CYP4B1	1	1-47279899-G-A	rs138537119	Ultra-rare
CYP4B1	1	1-47280746-GAT-G	rs3215983	Rare
CYP4B1	1	1-47280852-A-C	rs12094024	Common
CYP4B1	1	1-47282755-G-C	rs59694031	Common
CYP4B1	1	1-47282772-C-T	rs2297809	Common
CYP4B1	1	1-47282784-C-A	1-47282784-C-A	Ultra-rare
CYP4B1	1	1-47283818-C-T	rs115133540	Ultra-rare
CYP4B1	1	1-47283845-C-T	rs199759345	Ultra-rare
CYP4B1	1	1-47284367-C-T	rs138678209	Ultra-rare
CYP4A11	1	1-47398654-C-T	rs186780775	Ultra-rare
CYP4A11	1	1-47402373-T-C	rs143639289	Ultra-rare
CYP4Z1	1	1-47533164-T-C	rs1225382713	Ultra-rare
CYP4Z1	1	1-47548050-C-T	rs146576339	Ultra-rare
CYP4Z1	1	1-47560310-G-A	rs557601500	Ultra-rare

CYP4Z1	1	1-47560343-T-C	1-47560343-T-C	Ultra-rare
GPX7	1	1-53068210-CCG-C	1-53068210-CCG-C	Ultra-rare
GPX7	1	1-53072432-G-C	1-53072432-G-C	Ultra-rare
GPX7	1	1-53072617-C-T	rs201645639	Ultra-rare
GPX7	1	1-53073964-TC-T	rs751752130	Ultra-rare
CYP2J2	1	1-60375500-C-A	rs141697212	Ultra-rare
CYP2J2	1	1-60377389-A-T	rs66515830	Ultra-rare
CYP2J2	1	1-60377841-CT-C	1-60377841-CT-C	Ultra-rare
CYP2J2	1	1-60377881-A-C	rs112414284	Ultra-rare
CYP2J2	1	1-60377965-C-T	rs112540103	Ultra-rare
ABCA4	1	1-94463662-C-T	rs545397722	Ultra-rare
ABCA4	1	1-94466624-C-T	rs62642564	Rare
ABCA4	1	1-94495983-C-T	rs200967229	Ultra-rare
ABCA4	1	1-94502720-G-C	rs531449824	Ultra-rare
ABCA4	1	1-94506959-C-T	rs544428779	Ultra-rare
ABCA4	1	1-94510248-C-G	rs61749455	Ultra-rare
ABCA4	1	1-94510253-A-G	rs61749454	Ultra-rare
ABCA4	1	1-94578549-G-A	rs143207212	Ultra-rare
DPYD	1	1-97770919-A-C	rs60511679	Ultra-rare
DPYD	1	1-97915614-C-T	rs3918290	Ultra-rare
DPYD	1	1-97981407-C-T	rs142619737	Ultra-rare
DPYD	1	1-98015291-G-A	rs72975710	Ultra-rare
DPYD	1	1-98348909-G-A	rs72549310	Ultra-rare
GSTM4	1	1-110200392-A-T	rs146365598	Ultra-rare
GSTM4	1	1-110200405-G-A	rs139656805	Ultra-rare
GSTM4	1	1-110201647-A-G	rs528464207	Ultra-rare
GSTM4	1	1-110203823-C-T	rs150650325	Rare
GSTM2	1	1-110211967-G-A	rs147235683	Ultra-rare
GSTM5	1	1-110255238-A-G	rs113627447	Ultra-rare
GSTM5	1	1-110257647-T-C	rs150881777	Ultra-rare
GSTM5	1	1-110257665-G-A	rs371246652	Ultra-rare
GSTM3	1	1-110280811-C-T	1-110280811-C-T	Ultra-rare
GSTM3	1	1-110280921-C-T	rs146952826	Ultra-rare
GSTM3	1	1-110283134-C-G	rs36210756	Rare
SLC16A1	1	1-113459965-C-T	rs140728650	Ultra-rare
SLC16A1	1	1-113460049-G-A	rs77373295	Ultra-rare
SLC16A1	1	1-113460439-G-A	rs574990589	Ultra-rare
SLC22A15	1	1-116562305-C-T	rs147916319	Ultra-rare
SLC22A15	1	1-116562311-G-A	rs559932803	Ultra-rare
SLC22A15	1	1-116563420-T-A	rs371564095	Ultra-rare
SLC22A15	1	1-116577948-GGTGA-G	rs770075792	Ultra-rare
FMO5	1	1-146658627-C-T	rs376858269	Ultra-rare
FMO5	1	1-146680559-C-T	rs142613638	Ultra-rare
FMO5	1	1-146680614-C-A	rs142716484	Ultra-rare
FMO5	1	1-146684074-G-A	rs143647812	Ultra-rare
FMO5	1	1-146684908-T-G	rs587652629	Ultra-rare
FMO5	1	1-146684977-C-A	1-146684977-C-A	Ultra-rare

NR1I3	1	1-161200948-G-A	rs1295789450	Ultra-rare
NR1I3	1	1-161205730-A-AGG	rs767358929	Ultra-rare
ALDH9A1	1	1-165638228-C-T	1-165638228-C-T	Ultra-rare
FMO3	1	1-171076972-T-C	rs564555482	Ultra-rare
FMO3	1	1-171077292-G-A	rs143406401	Ultra-rare
FMO3	1	1-171079979-G-A	rs774866097	Ultra-rare
FMO3	1	1-171083296-T-C	1-171083296-T-C	Ultra-rare
FMO3	1	1-171086373-G-C	rs550307887	Ultra-rare
FMO3	1	1-171086458-G-A	rs373733184	Ultra-rare
FMO6P	1	1-171112490-C-T	rs1736565	Very-Common
FMO6P	1	1-171123353-G-T	rs368734934	Ultra-rare
FMO2	1	1-171154495-AAAGGT-A	rs531458466	Rare
FMO2	1	1-171165802-TG-T	rs28369860	Very-Common
FMO2	1	1-171168607-CT-C	rs766403374	Ultra-rare
FMO2	1	1-171173005-T-C	rs142177103	Ultra-rare
FMO2	1	1-171173043-C-T	rs574231506	Ultra-rare
FMO2	1	1-171173044-G-A	rs147227176	Ultra-rare
FMO2	1	1-171173058-G-A	rs139888422	Ultra-rare
FMO2	1	1-171173121-C-T	rs2020866	Rare
FMO2	1	1-171176929-TGTA-T	rs1291645093	Ultra-rare
FMO2	1	1-171178090-T-C	rs6661174	Common
FMO1	1	1-171236784-C-G	rs1284949597	Ultra-rare
FMO1	1	1-171244594-T-C	rs1438470609	Ultra-rare
FMO1	1	1-171248003-C-T	rs146934200	Ultra-rare
FMO1	1	1-171251278-G-A	rs149030329	Ultra-rare
FMO1	1	1-171251347-A-G	rs145415338	Ultra-rare
FMO1	1	1-171254588-C-T	rs60639054	Rare
FMO4	1	1-171289074-T-C	rs72549338	Ultra-rare
FMO4	1	1-171310716-G-A	rs148222001	Ultra-rare
HSD11B1	1	1-209879288-T-A	1-209879288-T-A	Ultra-rare
EPHX1	1	1-226019508-G-A	rs58623835	Rare
EPHX1	1	1-226019583-A-G	rs753521411	Ultra-rare
EPHX1	1	1-226026444-G-A	rs1364453923	Ultra-rare
SLC5A6	2	2-27427681-G-A	rs151071040	Ultra-rare
XDH	2	2-31560538-C-T	rs138249576	Ultra-rare
XDH	2	2-31562462-G-A	rs116290580	Ultra-rare
XDH	2	2-31571222-G-A	rs536923494	Ultra-rare
XDH	2	2-31572992-G-A	rs669884	Ultra-rare
XDH	2	2-31588939-G-A	rs148904866	Ultra-rare
XDH	2	2-31589852-A-AT	rs141470276	Ultra-rare
XDH	2	2-31593309-C-T	rs1257515718	Ultra-rare
XDH	2	2-31595224-G-A	rs139772558	Ultra-rare
XDH	2	2-31598320-T-G	2-31598320-T-G	Ultra-rare
XDH	2	2-31621501-A-T	rs547147775	Ultra-rare
PLGLB1	2	2-87248903-C-A	rs558829030	Rare
CHST10	2	2-101009781-G-A	rs370119165	Ultra-rare
CHST10	2	2-101011972-G-A	rs751768449	Ultra-rare

SULT1C2	2	2-108910209-G-A	rs140199800	Ultra-rare
SULT1C2	2	2-108910259-A-C	rs867745735	Ultra-rare
SULT1C2	2	2-108910781-T-C	rs1037064021	Ultra-rare
SULT1C2	2	2-108924881-GA-G	rs143858235	Rare
ABCB11	2	2-169780296-G-A	rs555881834	Ultra-rare
ABCB11	2	2-169787318-G-A	rs72549396	Ultra-rare
DHRS9	2	2-169926638-A-T	rs144161279	Ultra-rare
DHRS9	2	2-169939847-G-C	2-169939847-G-C	Ultra-rare
DHRS9	2	2-169948319-G-T	2-169948319-G-T	Ultra-rare
DHRS9	2	2-169952173-G-C	rs11695788	Ultra-rare
DHRS9	2	2-169952195-C-T	2-169952195-C-T	Ultra-rare
AOX1	2	2-201467018-C-T	rs377316171	Ultra-rare
AOX1	2	2-201469485-C-T	rs538141326	Ultra-rare
AOX1	2	2-201499512-A-G	rs563669050	Ultra-rare
AOX1	2	2-201499577-C-T	rs141342059	Ultra-rare
AOX1	2	2-201501692-G-A	rs113582006	Ultra-rare
AOX1	2	2-201502978-G-C	rs377368396	Ultra-rare
AOX1	2	2-201505908-G-T	rs199507417	Ultra-rare
AOX1	2	2-201524002-G-A	rs139975106	Ultra-rare
AOX1	2	2-201527655-C-A	rs143571444	Ultra-rare
AOX1	2	2-201533366-G-A	rs368816835	Ultra-rare
AOX1	2	2-201533371-T-C	rs150568828	Ultra-rare
CYP20A1	2	2-204111581-C-T	rs150778410	Ultra-rare
CYP27A1	2	2-219674423-C-T	rs201114717	Ultra-rare
CYP27A1	2	2-219677301-C-T	rs114768494	Rare
CYP27A1	2	2-219677744-ACTGGC-A	rs1237201999	Ultra-rare
CYP27A1	2	2-219679347-G-A	rs370868184	Ultra-rare
ABCB6	2	2-220075736-C-T	rs757264353	Ultra-rare
ABCB6	2	2-220077194-C-T	2-220077194-C-T	Ultra-rare
ABCB6	2	2-220077961-AC-A	rs780847738	Ultra-rare
ABCB6	2	2-220078890-C-T	rs111677240	Ultra-rare
ABCB6	2	2-220080761-C-T	rs199906854	Ultra-rare
ABCB6	2	2-220083019-AC-A	rs377591749	Ultra-rare
UGT1A10	2	2-234545559-C-T	rs539495441	Ultra-rare
UGT1A10	2	2-234545928-C-T	rs61748821	Ultra-rare
UGT1A7	2	2-234590697-G-A	rs200190624	Ultra-rare
UGT1A7	2	2-234590826-C-A	rs149618508	Ultra-rare
UGT1A7	2	2-234590848-GA-G	rs755165543	Ultra-rare
UGT1A8	2	2-234638282-G-GT	rs544798573	Rare
UGT1A3	2	2-234638282-G-GT	rs544798573	Rare
UGT1A1	2	2-234680951-C-T	rs201427749	Ultra-rare
UGT1A8	2	2-234680951-C-T	rs201427749	Ultra-rare
UGT1A10	2	2-234680951-C-T	rs201427749	Ultra-rare
UGT1A9	2	2-234680951-C-T	rs201427749	Ultra-rare
UGT1A7	2	2-234680951-C-T	rs201427749	Ultra-rare
UGT1A6	2	2-234680951-C-T	rs201427749	Ultra-rare
UGT1A5	2	2-234680951-C-T	rs201427749	Ultra-rare

UGT1A4	2	2-234680951-C-T	rs201427749	Ultra-rare
UGT1A3	2	2-234680951-C-T	rs201427749	Ultra-rare
UGT1A1	2	2-234680973-T-A	rs527798161	Ultra-rare
UGT1A8	2	2-234680973-T-A	rs527798161	Ultra-rare
UGT1A10	2	2-234680973-T-A	rs527798161	Ultra-rare
UGT1A9	2	2-234680973-T-A	rs527798161	Ultra-rare
UGT1A7	2	2-234680973-T-A	rs527798161	Ultra-rare
UGT1A6	2	2-234680973-T-A	rs527798161	Ultra-rare
UGT1A5	2	2-234680973-T-A	rs527798161	Ultra-rare
UGT1A4	2	2-234680973-T-A	rs527798161	Ultra-rare
UGT1A3	2	2-234680973-T-A	rs527798161	Ultra-rare
SLC6A6	3	3-14485135-G-A	rs200896852	Ultra-rare
SLC22A13	3	3-38307637-C-T	rs201826365	Ultra-rare
SLC22A13	3	3-38316168-C-A	rs149591926	Ultra-rare
SLC22A13	3	3-38316184-A-G	rs369864611	Ultra-rare
SLC22A13	3	3-38316564-C-T	rs557335370	Ultra-rare
SLC22A13	3	3-38316881-G-A	rs149646211	Ultra-rare
SLC22A13	3	3-38317106-T-C	rs565634623	Ultra-rare
SLC22A13	3	3-38317561-G-A	3-38317561-G-A	Ultra-rare
SLC22A14	3	3-38347916-G-A	3-38347916-G-A	Ultra-rare
SLC22A14	3	3-38348743-A-C	rs753331	Ultra-rare
SLC22A14	3	3-38349064-C-G	rs77826783	Rare
SLC22A14	3	3-38350471-C-T	rs146067679	Ultra-rare
SLC22A14	3	3-38354498-C-T	rs116032919	Ultra-rare
CYP8B1	3	3-42917014-A-G	rs549215878	Ultra-rare
NR1I2	3	3-119529026-G-A	rs569524145	Ultra-rare
NR1I2	3	3-119533824-A-G	rs149853698	Ultra-rare
NR1I2	3	3-119534575-C-T	rs747428529	Ultra-rare
SLC15A2	3	3-121613331-CT-C	rs750091124	Ultra-rare
SLC15A2	3	3-121641623-T-C	rs766556713	Ultra-rare
SLC15A2	3	3-121641670-C-T	rs145317059	Ultra-rare
SLC15A2	3	3-121648196-G-A	rs539838680	Ultra-rare
CHST13	3	3-126260895-A-G	3-126260895-A-G	Ultra-rare
SLCO2A1	3	3-133661524-G-A	rs773837399	Ultra-rare
SLCO2A1	3	3-133664004-A-G	rs140319395	Ultra-rare
SLCO2A1	3	3-133666200-G-A	rs759989525	Ultra-rare
SLCO2A1	3	3-133698324-C-T	rs149946676	Ultra-rare
ABCC5	3	3-183681378-T-C	rs201252879	Ultra-rare
SOD3	4	4-24801509-C-A	rs755086508	Ultra-rare
UGT2B17	4	4-69403384-G-A	rs558443905	Ultra-rare
UGT2B17	4	4-69403599-A-G	rs138121512	Ultra-rare
UGT2B17	4	4-69416569-C-T	rs145097129	Ultra-rare
UGT2B17	4	4-69416590-A-T	4-69416590-A-T	Ultra-rare
UGT2B17	4	4-69426263-G-T	rs1373834972	Ultra-rare
UGT2B15	4	4-69512863-G-A	rs143480699	Rare
UGT2B15	4	4-69533802-A-C	rs141576110	Ultra-rare
UGT2B15	4	4-69535726-T-A	4-69535726-T-A	Ultra-rare

UGT2B10	4	4-69683837-T-C	4-69683837-T-C	Ultra-rare
UGT2B10	4	4-69687987-C-A	rs2942857	Very-Common
UGT2B10	4	4-69692127-G-C	rs535990845	Ultra-rare
UGT2B10	4	4-69692173-A-T	rs747382511	Ultra-rare
UGT2B10	4	4-69693141-GT-G	rs201232361	Rare
UGT2B10	4	4-69693267-G-A	rs201190671	Ultra-rare
UGT2B10	4	4-69696457-ACCTG-A	rs774188592	Ultra-rare
UGT2B10	4	4-69696457-ACCTG-A	4-69696457-ACCTG-A	Ultra-rare
UGT2B10	4	4-69696461-G-GAAGA	rs759207345	Ultra-rare
UGT2B10	4	4-69696539-GT-G	rs867759745	Ultra-rare
UGT2B11	4	4-70066376-C-G	rs144149579	Ultra-rare
UGT2B11	4	4-70066399-T-G	rs369662379	Ultra-rare
UGT2B11	4	4-70066434-A-T	4-70066434-A-T	Ultra-rare
UGT2B11	4	4-70070337-T-A	4-70070337-T-A	Ultra-rare
UGT2B11	4	4-70070367-C-T	rs146890806	Ultra-rare
UGT2B11	4	4-70071216-G-A	rs72551397	Ultra-rare
UGT2B11	4	4-70078393-C-T	rs138593124	Rare
UGT2B28	4	4-70148285-C-T	rs140267024	Ultra-rare
UGT2B28	4	4-70160367-G-C	rs373902503	Ultra-rare
UGT2B4	4	4-70346581-G-A	rs184647368	Ultra-rare
UGT2A1	4	4-70455305-G-A	rs139303872	Ultra-rare
UGT2A1	4	4-70460384-T-C	rs150970095	Ultra-rare
UGT2A1	4	4-70460903-C-A	4-70460903-C-A	Ultra-rare
UGT2A1	4	4-70460939-G-C	rs141445080	Ultra-rare
UGT2A1	4	4-70462042-C-T	rs4148301	Rare
UGT2A1	4	4-70464988-G-T	rs556762743	Ultra-rare
UGT2A1	4	4-70465031-C-T	rs374804650	Ultra-rare
UGT2A1	4	4-70504696-AT-A	rs571556296	Ultra-rare
UGT2A1	4	4-70504779-TC-T	4-70504779-TC-T	Ultra-rare
UGT2A1	4	4-70512787-A-T	rs111696697	Ultra-rare
SULT1B1	4	4-70599966-C-A	4-70599966-C-A	Ultra-rare
SULT1E1	4	4-70707802-A-AT	rs751033828	Ultra-rare
METAP1	4	4-99982364-C-T	rs200609570	Ultra-rare
ADH5	4	4-99997442-C-T	rs572412641	Ultra-rare
ADH4	4	4-100052674-GCA-G	rs29001206	Ultra-rare
ADH6	4	4-100126140-DEL8-G	rs541657822	Ultra-rare
ADH6	4	4-100134836-A-T	rs369787696	Ultra-rare
ADH1A	4	4-100201426-A-G	rs199556184	Ultra-rare
ADH1A	4	4-100203735-A-G	rs137874792	Ultra-rare
ADH1A	4	4-100205641-A-G	rs116276567	Ultra-rare
ADH1B	4	4-100229017-G-A	rs2066702	Common
ADH1B	4	4-100235171-C-A	rs1181667845	Ultra-rare
ADH1B	4	4-100237059-G-A	rs140615334	Ultra-rare
ADH1C	4	4-100268190-A-C	rs283413	Very-Common
ADH1C	4	4-100268940-C-CT	rs773744058	Ultra-rare
ADH1C	4	4-100273830-C-T	rs572969595	Ultra-rare
ADH7	4	4-100349052-C-T	rs146508025	Ultra-rare

GSTCD	4	4-106639039-T-G	rs1055999004	Ultra-rare
GSTCD	4	4-106639176-T-A	rs61736388	Ultra-rare
UGT8	4	4-115544172-C-G	rs150551951	Ultra-rare
SLCO4C1	5	5-101583135-G-C	rs571659153	Ultra-rare
SLCO4C1	5	5-101592985-C-T	rs546037634	Ultra-rare
SLCO4C1	5	5-101597690-A-G	rs139786996	Ultra-rare
SLCO4C1	5	5-101599452-C-T	5-101599452-C-T	Ultra-rare
SLCO6A1	5	5-101748731-G-A	rs149593558	Ultra-rare
SLCO6A1	5	5-101748782-T-C	rs571969326	Ultra-rare
ALDH7A1	5	5-125930856-GC-G	rs750693623	Ultra-rare
SLC22A4	5	5-131630380-T-C	5-131630380-T-C	Ultra-rare
SLC22A4	5	5-131662989-C-T	rs11568503	Ultra-rare
SLC22A5	5	5-131719994-G-T	rs386134200	Ultra-rare
SLC22A5	5	5-131722742-C-T	rs150278881	Ultra-rare
SLC22A5	5	5-131724671-C-T	rs142479732	Ultra-rare
SLC22A5	5	5-131728202-T-G	rs11568514	Ultra-rare
SLC22A5	5	5-131728303-C-A	rs569268772	Ultra-rare
TPMT	6	6-18130918-T-C	rs1142345	Rare
TPMT	6	6-18139201-G-A	rs112339338	Ultra-rare
ALDH5A1	6	6-24503570-G-A	rs369366567	Ultra-rare
ALDH5A1	6	6-24515483-G-T	rs756034595	Ultra-rare
ALDH5A1	6	6-24523143-C-T	rs536355368	Ultra-rare
ALDH5A1	6	6-24528372-G-A	rs1041467895	Ultra-rare
ALDH5A1	6	6-24533753-A-C	rs142482046	Ultra-rare
GPX6	6	6-28472175-A-G	rs766809704	Ultra-rare
GPX6	6	6-28474140-T-C	6-28474140-T-C	Ultra-rare
GPX5	6	6-28501909-A-T	6-28501909-A-T	Ultra-rare
CYP21A2	6	6-32007203-T-A	rs6475	Ultra-rare
CYP21A2	6	6-32008696-G-A	rs72552758	Ultra-rare
TAP2	6	6-32782196-G-A	6-32782196-G-A	Ultra-rare
TAP2	6	6-32782294-T-C	rs867329348	Ultra-rare
TAP2	6	6-32800433-C-A	6-32800433-C-A	Ultra-rare
TAP2	6	6-32802939-G-A	rs140532996	Ultra-rare
TAP1	6	6-32818230-G-A	rs147332077	Ultra-rare
PPARD	6	6-35391891-G-A	rs768763586	Ultra-rare
SLC22A7	6	6-43269942-G-A	rs1205738663	Ultra-rare
ABCC10	6	6-43400685-G-A	rs140348023	Ultra-rare
ABCC10	6	6-43400886-C-T	rs528202255	Ultra-rare
ABCC10	6	6-43400887-G-A	rs185652890	Ultra-rare
ABCC10	6	6-43403513-C-T	rs143947606	Ultra-rare
ABCC10	6	6-43409670-G-A	rs150052458	Ultra-rare
ABCC10	6	6-43412082-C-T	rs41281802	Rare
ABCC10	6	6-43413374-G-A	rs141037448	Ultra-rare
ABCC10	6	6-43413574-C-T	rs187313814	Ultra-rare
ABCC10	6	6-43413587-G-T	rs530304629	Ultra-rare
ABCC10	6	6-43413595-A-G	rs149560171	Ultra-rare
ABCC10	6	6-43415516-C-T	rs571262016	Ultra-rare

CYP39A1	6	6-46518175-CT-C	6-46518175-CT-C	Ultra-rare
CYP39A1	6	6-46554898-C-T	rs371505179	Ultra-rare
CYP39A1	6	6-46555791-C-A	rs772043783	Ultra-rare
CYP39A1	6	6-46563779-C-T	rs147961800	Ultra-rare
GSTA2	6	6-52617776-T-G	6-52617776-T-G	Ultra-rare
GSTA2	6	6-52619786-G-T	rs527479150	Ultra-rare
GSTA1	6	6-52659056-A-AT	rs1284050982	Ultra-rare
GSTA1	6	6-52661005-C-T	rs373523033	Ultra-rare
GSTA5	6	6-52697739-C-T	rs147886318	Ultra-rare
GSTA3	6	6-52768525-A-G	rs1223140731	Ultra-rare
GSTA4	6	6-52843339-G-T	rs1326100763	Ultra-rare
DDO	6	6-110714020-A-T	6-110714020-A-T	Ultra-rare
DDO	6	6-110714161-C-A	rs747822396	Ultra-rare
DDO	6	6-110714358-G-A	rs760181658	Ultra-rare
DDO	6	6-110714378-G-C	6-110714378-G-C	Ultra-rare
DDO	6	6-110714514-G-A	6-110714514-G-A	Ultra-rare
DDO	6	6-110729555-C-T	6-110729555-C-T	Ultra-rare
DDO	6	6-110736730-C-T	rs141023778	Rare
SLC22A16	6	6-110759997-C-T	rs149722400	Ultra-rare
SLC22A16	6	6-110760008-A-G	rs12210538	Ultra-rare
SLC22A16	6	6-110763914-C-T	rs369769480	Ultra-rare
SLC22A16	6	6-110763935-T-C	rs41288594	Ultra-rare
SLC22A16	6	6-110768093-G-A	rs370297042	Ultra-rare
ALDH8A1	6	6-135254132-C-T	rs142034907	Ultra-rare
SLC22A1	6	6-160543328-T-TG	rs111863691	Ultra-rare
SLC22A1	6	6-160560824-G-A	rs34130495	Ultra-rare
SLC22A1	6	6-160560897-DEL6-C	rs113569197	Very-Common
SLC22A1	6	6-160564603-G-A	6-160564603-G-A	Ultra-rare
SLC22A2	6	6-160645750-C-T	rs372435157	Ultra-rare
SLC22A2	6	6-160662547-C-T	rs151282335	Ultra-rare
SLC22A2	6	6-160664685-G-T	6-160664685-G-T	Ultra-rare
SLC22A2	6	6-160668263-T-A	rs149782050	Ultra-rare
SLC22A2	6	6-160671647-C-T	rs145289005	Ultra-rare
SLC22A3	6	6-160769825-TG-T	rs764599660	Ultra-rare
SLC22A3	6	6-160831795-C-G	6-160831795-C-G	Ultra-rare
SLC22A3	6	6-160858162-C-T	rs1046869631	Ultra-rare
SLC22A3	6	6-160864759-C-T	rs961213755	Ultra-rare
SLC22A3	6	6-160872065-CCA-C	rs1463659154	Ultra-rare
CHST12	7	7-2443271-DEL5-C	rs1159679670	Rare
AHR	7	7-17367452-G-C	rs200257782	Ultra-rare
AHR	7	7-17373671-C-T	rs140380733	Ultra-rare
ABCB5	7	7-20666235-G-C	rs17143187	Rare
ABCB5	7	7-20682892-C-T	rs141511940	Ultra-rare
ABCB5	7	7-20687233-C-T	rs150279505	Ultra-rare
ABCB5	7	7-20691193-G-T	7-20691193-G-T	Ultra-rare
ABCB5	7	7-20698221-CA-C	rs144050488	Ultra-rare
ABCB5	7	7-20721165-G-A	rs139913454	Ultra-rare

ABCB5	7	7-20725325-A-T	rs76179099	Rare
ABCB5	7	7-20738068-G-C	rs145758664	Ultra-rare
ABCB5	7	7-20738157-T-C	rs200222060	Ultra-rare
ABCB5	7	7-20739715-C-T	rs141490175	Ultra-rare
ABCB5	7	7-20744360-T-G	rs139933666	Ultra-rare
ABCB5	7	7-20744384-G-A	rs140688625	Ultra-rare
ABCB5	7	7-20766691-G-A	rs111647033	Ultra-rare
ABCB5	7	7-20766691-G-C	7-20766691-G-C	Ultra-rare
ABCB5	7	7-20782614-G-C	rs139479024	Ultra-rare
ABCB5	7	7-20782629-A-G	rs189610663	Ultra-rare
ABCB5	7	7-20784952-T-C	rs143407594	Ultra-rare
ABCB5	7	7-20785017-G-A	rs146066744	Ultra-rare
ABCB5	7	7-20793028-G-T	rs142824450	Ultra-rare
ABCB5	7	7-20793113-A-G	rs58795451	Common
ABCB5	7	7-20795056-C-T	rs146527949	Rare
POR	7	7-75608839-A-C	7-75608839-A-C	Ultra-rare
POR	7	7-75612857-C-A	rs72557937	Ultra-rare
POR	7	7-75614485-G-A	rs375535318	Ultra-rare
POR	7	7-75615107-G-A	rs569673150	Ultra-rare
POR	7	7-75615113-G-A	rs121912976	Ultra-rare
ABCB4	7	7-87037381-C-T	rs147171162	Ultra-rare
ABCB4	7	7-87037501-G-A	rs371152960	Ultra-rare
ABCB4	7	7-87041333-C-T	rs61730509	Ultra-rare
ABCB4	7	7-87042969-T-C	rs138595432	Ultra-rare
ABCB4	7	7-87049319-T-A	7-87049319-T-A	Ultra-rare
ABCB4	7	7-87049345-C-T	rs8187801	Common
ABCB4	7	7-87060844-C-T	rs45575636	Ultra-rare
ABCB4	7	7-87072692-A-C	rs201168284	Ultra-rare
ABCB4	7	7-87092204-CT-C	7-87092204-CT-C	Ultra-rare
ABCB1	7	7-87133706-G-T	7-87133706-G-T	Ultra-rare
ABCB1	7	7-87150095-T-G	rs148718120	Ultra-rare
ABCB1	7	7-87165800-C-T	rs200754866	Ultra-rare
ABCB1	7	7-87179855-G-T	7-87179855-G-T	Ultra-rare
PON1	7	7-94928288-CTG-C	rs779104502	Ultra-rare
PON1	7	7-94928294-C-A	rs368206333	Ultra-rare
PON1	7	7-94937498-DEL4-G	rs778908135	Ultra-rare
PON1	7	7-94940859-T-C	rs536888659	Ultra-rare
PON3	7	7-94993202-T-C	rs780945396	Ultra-rare
PON3	7	7-94996689-T-C	rs140234471	Ultra-rare
PON3	7	7-95001515-G-T	rs148345439	Ultra-rare
PON3	7	7-95001585-A-C	rs561570115	Ultra-rare
PON3	7	7-95025661-A-G	rs368482537	Ultra-rare
CYP3A5	7	7-99250393-T-TA	rs41303343	Common
CYP3A5	7	7-99260462-G-T	rs111371159	Ultra-rare
CYP3A5	7	7-99264299-A-G	rs142823108	Ultra-rare
CYP3A7	7	7-99308452-G-T	rs61737520	Ultra-rare
CYP3A7	7	7-99317941-G-A	rs116248418	Ultra-rare

CYP3A4	7	7-99358480-G-A	rs530166880	Ultra-rare
CYP3A4	7	7-99359763-C-G	7-99359763-C-G	Ultra-rare
CYP3A4	7	7-99370218-G-A	rs142296281	Ultra-rare
CYP3A43	7	7-99434077-TA-T	rs61469810	Very-Common
CYP3A43	7	7-99445181-G-A	rs78548296	Ultra-rare
CYP3A43	7	7-99459258-T-C	7-99459258-T-C	Ultra-rare
CYP3A43	7	7-99461160-G-T	rs143991326	Rare
CFTR	7	7-117171011-C-G	rs140502196	Ultra-rare
CFTR	7	7-117176664-T-C	rs201016820	Ultra-rare
CFTR	7	7-117232062-A-G	rs201124247	Ultra-rare
CFTR	7	7-117232713-T-C	rs1057516216	Ultra-rare
CFTR	7	7-117246808-G-A	rs75096551	Ultra-rare
CFTR	7	7-117250690-A-G	7-117250690-A-G	Ultra-rare
SLC13A1	7	7-122757625-A-G	7-122757625-A-G	Ultra-rare
SLC13A1	7	7-122757633-A-T	7-122757633-A-T	Ultra-rare
SLC13A1	7	7-122809322-C-T	rs188469513	Ultra-rare
SLC13A1	7	7-122821122-C-T	rs199650403	Ultra-rare
GSTK1	7	7-142961207-A-T	rs546809728	Ultra-rare
GSTK1	7	7-142961770-G-A	rs766555447	Ultra-rare
GSTK1	7	7-142962156-C-T	rs538829875	Ultra-rare
GSTK1	7	7-142964708-A-C	rs570471159	Ultra-rare
GSTK1	7	7-142965251-G-A	rs746245994	Ultra-rare
NOS3	7	7-150696390-T-G	rs201023253	Ultra-rare
NOS3	7	7-150698356-C-T	rs578101703	Ultra-rare
NOS3	7	7-150698385-G-A	rs563793183	Ultra-rare
NOS3	7	7-150698401-G-C	rs375829467	Ultra-rare
NOS3	7	7-150707251-C-T	rs148919189	Ultra-rare
ABCB8	7	7-150732812-C-T	rs17545756	Ultra-rare
ABCB8	7	7-150739187-C-T	rs201301011	Ultra-rare
ABCB8	7	7-150741183-C-T	rs147294765	Ultra-rare
ABCB8	7	7-150741247-G-A	rs755235496	Ultra-rare
NAT1	8	8-18068701-G-A	rs567557602	Ultra-rare
NAT1	8	8-18079983-C-T	8-18079983-C-T	Ultra-rare
NAT1	8	8-18080038-T-C	rs554793519	Ultra-rare
NAT1	8	8-18080115-C-T	rs5030839	Ultra-rare
NAT2	8	8-18257761-G-C	rs561124342	Ultra-rare
NAT2	8	8-18258082-A-G	rs374761885	Ultra-rare
NAT2	8	8-18258091-C-T	rs79050330	Ultra-rare
NAT2	8	8-18258102-C-T	rs375746304	Ultra-rare
NAT2	8	8-18258220-G-A	rs149460636	Ultra-rare
EPHX2	8	8-27358529-T-A	rs138702597	Ultra-rare
EPHX2	8	8-27361179-T-C	8-27361179-T-C	Ultra-rare
EPHX2	8	8-27362623-A-G	rs146049019	Ultra-rare
EPHX2	8	8-27373271-G-A	rs546857733	Ultra-rare
EPHX2	8	8-27373857-AG-A	8-27373857-AG-A	Ultra-rare
EPHX2	8	8-27373865-G-A	rs751141	Common
EPHX2	8	8-27394305-T-G	rs578026023	Ultra-rare

EPHX2	8	8-27399010-G-A	rs201410558	Ultra-rare
GSR	8	8-30538494-G-A	rs1213868590	Ultra-rare
GSR	8	8-30553995-G-A	rs145851500	Ultra-rare
GSR	8	8-30565624-G-A	rs8190955	Common
CYP7A1	8	8-59409292-C-T	rs139396617	Ultra-rare
CYP7A1	8	8-59412656-A-G	rs111811966	Ultra-rare
CYP7B1	8	8-65517252-A-G	rs769450032	Ultra-rare
ADHFE1	8	8-67356627-G-C	rs146390329	Ultra-rare
ADHFE1	8	8-67357506-C-A	rs139870830	Ultra-rare
ADHFE1	8	8-67357532-A-T	8-67357532-A-T	Ultra-rare
ADHFE1	8	8-67361139-T-C	rs577285802	Ultra-rare
ADHFE1	8	8-67364205-A-G	rs780608528	Ultra-rare
ADHFE1	8	8-67364259-G-A	rs147236271	Ultra-rare
ADHFE1	8	8-67366374-A-T	rs147833015	Ultra-rare
ADHFE1	8	8-67369088-G-C	rs141310107	Ultra-rare
SULF1	8	8-70514019-G-A	rs61747207	Ultra-rare
SULF1	8	8-70517163-G-A	rs148982651	Ultra-rare
SULF1	8	8-70550976-A-G	rs527934534	Ultra-rare
SLCO5A1	8	8-70585516-G-A	rs546509292	Ultra-rare
CYP11B1	8	8-143956570-T-C	rs201300785	Ultra-rare
CYP11B1	8	8-143957183-G-A	rs146124466	Ultra-rare
CYP11B1	8	8-143958274-C-T	rs200559974	Ultra-rare
ALDH1B1	9	9-38395993-C-T	9-38395993-C-T	Ultra-rare
ALDH1B1	9	9-38396773-CA-C	rs554947716	Ultra-rare
ALDH1A1	9	9-75516186-C-T	rs371621448	Ultra-rare
SLC28A3	9	9-86900443-G-GT	rs557666022	Ultra-rare
SLC28A3	9	9-86903030-T-G	rs147279405	Ultra-rare
SLC28A3	9	9-86905173-G-A	rs1357733836	Ultra-rare
SLC28A3	9	9-86912924-C-A	rs543523369	Ultra-rare
SLC28A3	9	9-86917301-T-C	rs10868138	Common
SLC28A3	9	9-86924542-A-G	rs113754177	Ultra-rare
ABCA1	9	9-107547916-C-T	9-107547916-C-T	Ultra-rare
ABCA1	9	9-107564426-G-A	rs74913556	Ultra-rare
ABCA1	9	9-107574880-C-T	rs576515619	Ultra-rare
ABCA1	9	9-107591346-T-A	9-107591346-T-A	Ultra-rare
ABCA1	9	9-107599797-C-A	rs370223805	Ultra-rare
ABCA1	9	9-107646756-G-A	rs145183203	Ultra-rare
MAT1A	10	10-82040047-T-C	10-82040047-T-C	Ultra-rare
MAT1A	10	10-82043687-C-G	10-82043687-C-G	Ultra-rare
CYP26C1	10	10-94821884-G-A	rs140814709	Ultra-rare
CYP26C1	10	10-94828279-C-T	rs377151786	Ultra-rare
CYP26C1	10	10-94828392-G-C	rs753971732	Ultra-rare
CYP26A1	10	10-94834090-G-T	rs562861556	Ultra-rare
CYP26A1	10	10-94834195-T-G	rs780047549	Ultra-rare
CYP2C18	10	10-96447561-A-AT	rs372279673	Ultra-rare
CYP2C18	10	10-96447562-T-A	rs41291550	Common
CYP2C18	10	10-96447920-C-T	rs117111102	Ultra-rare

CYP2C18	10	10-96484186-G-C	rs1243906358	Ultra-rare
CYP2C18	10	10-96484276-T-A	rs531098917	Ultra-rare
CYP2C19	10	10-96522463-A-G	rs28399504	Ultra-rare
CYP2C19	10	10-96540330-C-T	rs183701923	Ultra-rare
CYP2C19	10	10-96540410-G-A	rs4986893	Ultra-rare
CYP2C19	10	10-96541615-C-T	rs6413438	Ultra-rare
CYP2C19	10	10-96612493-A-T	rs146991374	Ultra-rare
CYP2C19	10	10-96612671-A-C	rs55640102	Ultra-rare
CYP2C9	10	10-96698440-A-G	rs114071557	Ultra-rare
CYP2C9	10	10-96701991-G-A	rs72558189	Ultra-rare
CYP2C9	10	10-96702047-C-T	rs1799853	Ultra-rare
CYP2C9	10	10-96707592-T-C	rs367922573	Ultra-rare
CYP2C9	10	10-96709038-GA-G	rs9332131	Rare
CYP2C8	10	10-96798695-C-A	rs141209951	Ultra-rare
CYP2C8	10	10-96802804-A-G	rs146806199	Ultra-rare
CYP2C8	10	10-96818144-T-C	rs141120323	Ultra-rare
CYP2C8	10	10-96824564-C-T	rs146962089	Ultra-rare
CYP2C8	10	10-96827075-C-T	rs369591911	Ultra-rare
CYP2C8	10	10-96829159-T-C	rs142470035	Ultra-rare
ABCC2	10	10-101556880-C-T	rs201145273	Ultra-rare
ABCC2	10	10-101559094-A-G	rs17222674	Ultra-rare
ABCC2	10	10-101591865-C-T	rs757051605	Ultra-rare
ABCC2	10	10-101594193-C-A	rs767201064	Ultra-rare
ABCC2	10	10-101595869-C-T	rs377550597	Ultra-rare
ABCC2	10	10-101595954-G-A	rs139188247	Ultra-rare
ABCC2	10	10-101595975-G-T	rs8187692	Common
ABCC2	10	10-101601738-G-T	rs778603543	Ultra-rare
ABCC2	10	10-101603611-T-C	rs557494144	Ultra-rare
ABCC2	10	10-101610554-G-C	10-101610554-G-C	Ultra-rare
CYP17A1	10	10-104590626-G-A	rs957099355	Ultra-rare
CYP2E1	10	10-135352321-G-C	rs199855848	Ultra-rare
SLC22A18AS	11	11-2909505-T-TG	rs763131197	Rare
SLC22A18AS	11	11-2921021-C-T	rs542746590	Ultra-rare
SLC22A18	11	11-2929523-G-A	rs80148696	Ultra-rare
SLC22A18	11	11-2930934-CG-C	rs558203402	Rare
SLC22A18	11	11-2943342-G-T	rs144116326	Ultra-rare
PDE3B	11	11-14810745-C-T	rs781318091	Ultra-rare
PDE3B	11	11-14891030-C-T	rs200520115	Ultra-rare
CYP2R1	11	11-14900720-G-A	rs199883994	Ultra-rare
CYP2R1	11	11-14907393-A-G	rs61495246	Ultra-rare
KCNJ11	11	11-17408737-C-A	rs74339576	Ultra-rare
ABCC8	11	11-17415836-T-G	rs80075294	Ultra-rare
ABCC8	11	11-17417226-G-T	11-17417226-G-T	Ultra-rare
ABCC8	11	11-17449835-C-T	11-17449835-C-T	Ultra-rare
ABCC8	11	11-17464807-A-G	rs202183744	Ultra-rare
ABCC8	11	11-17482129-C-A	11-17482129-C-A	Ultra-rare
ABCC8	11	11-17491664-GA-G	rs776444419	Ultra-rare

ABCC8	11	11-17496432-C-T	11-17496432-C-T	Ultra-rare
CAT	11	11-34477565-A-G	rs1169723754	Ultra-rare
CAT	11	11-34478267-G-A	rs139421991	Ultra-rare
CAT	11	11-34489880-C-T	rs373491820	Ultra-rare
SLC22A6	11	11-62748503-G-A	rs150409056	Ultra-rare
SLC22A6	11	11-62748503-G-T	11-62748503-G-T	Ultra-rare
SLC22A6	11	11-62751153-G-A	rs746271227	Ultra-rare
SLC22A8	11	11-62762124-G-A	rs142362788	Rare
SLC22A8	11	11-62767307-G-A	rs45566039	Ultra-rare
SLC22A10	11	11-63057925-G-A	rs1790218	Very-Common
SLC22A10	11	11-63064782-C-T	rs111381363	Ultra-rare
SLC22A10	11	11-63064876-G-A	rs111835134	Ultra-rare
SLC22A10	11	11-63064888-G-A	rs377529003	Ultra-rare
SLC22A10	11	11-63065124-C-T	rs181427889	Ultra-rare
SLC22A10	11	11-63066985-G-A	11-63066985-G-A	Ultra-rare
SLC22A10	11	11-63067103-T-C	rs1455875575	Ultra-rare
SLC22A10	11	11-63072236-A-AT	rs568900537	Ultra-rare
SLC22A10	11	11-63078478-GA-G	rs562147200	Rare
SLC22A10	11	11-63078478-GA-GAA	11-63078478-GA-GAA	Ultra-rare
SLC22A9	11	11-63137932-T-C	11-63137932-T-C	Ultra-rare
SLC22A9	11	11-63138694-G-A	rs138297035	Ultra-rare
SLC22A9	11	11-63149670-CA-CAA	rs564236291	Rare
SLC22A9	11	11-63149670-CA-C	11-63149670-CA-CAA	Ultra-rare
SLC22A9	11	11-63175679-C-A	rs147323107	Ultra-rare
SLC22A12	11	11-64366395-G-A	rs534253348	Ultra-rare
SLC22A12	11	11-64367854-G-A	rs147647315	Rare
SLC29A2	11	11-66131708-C-T	rs1450926367	Ultra-rare
GSTP1	11	11-67353974-C-T	rs45549733	Ultra-rare
ALDH3B2	11	11-67432786-C-T	rs113681988	Ultra-rare
ALDH3B2	11	11-67432828-G-A	rs149110344	Ultra-rare
ALDH3B2	11	11-67432961-G-C	rs754993637	Ultra-rare
ALDH3B2	11	11-67432973-G-T	rs113469233	Ultra-rare
ALDH3B2	11	11-67433014-G-A	rs528990160	Ultra-rare
ALDH3B2	11	11-67441906-A-C	rs7947754	Very-Common
ALDH3B1	11	11-67782795-C-T	rs201119062	Ultra-rare
ALDH3B1	11	11-67786064-A-AC	rs397777471	Very-Common
ALDH3B1	11	11-67789293-G-GC	rs58160034	Very-Common
SLCO2B1	11	11-74883454-G-A	rs562515081	Ultra-rare
SLCO2B1	11	11-74915617-C-T	rs142700667	Ultra-rare
NNMT	11	11-114182827-T-A	11-114182827-T-A	Ultra-rare
MGST1	12	12-16500833-G-T	rs565820971	Ultra-rare
SLCO1C1	12	12-20852549-C-A	rs532692732	Ultra-rare
SLCO1C1	12	12-20876148-G-T	12-20876148-G-T	Ultra-rare
SLCO1B3	12	12-21011421-A-G	rs144099822	Rare
SLCO1B3	12	12-21028208-G-C	rs60140950	Ultra-rare
SLCO1B3	12	12-21030807-T-TA	rs748726828	Ultra-rare
SLCO1B3	12	12-21054342-G-A	rs550941268	Ultra-rare

SLCO1B1	12	12-21325663-T-A	rs774451034	Ultra-rare
SLCO1B1	12	12-21327574-T-C	rs865965255	Ultra-rare
SLCO1B1	12	12-21329793-T-G	rs553054459	Ultra-rare
SLCO1B1	12	12-21329832-G-T	rs77271279	Rare
SLCO1B1	12	12-21377774-G-A	rs200994482	Ultra-rare
SLCO1A2	12	12-21445125-G-T	12-21445125-G-T	Ultra-rare
SLCO1A2	12	12-21454128-A-G	rs374011740	Ultra-rare
SLCO1A2	12	12-21457448-G-A	rs11568564	Ultra-rare
IAPP	12	12-21526269-AG-A	rs897927917	Ultra-rare
CYP27B1	12	12-58156930-T-C	12-58156930-T-C	Ultra-rare
CYP27B1	12	12-58158294-G-A	rs745757851	Ultra-rare
ALDH2	12	12-112220981-T-C	rs140347209	Ultra-rare
ALDH2	12	12-112229932-G-A	rs773112716	Ultra-rare
NOS1	12	12-117665343-G-A	rs372289883	Ultra-rare
NOS1	12	12-117665377-G-T	rs563920841	Ultra-rare
NOS1	12	12-117672378-G-A	rs770948047	Ultra-rare
NOS1	12	12-117768808-G-A	rs549377164	Ultra-rare
DHRS12	13	13-52348092-C-T	rs1379695909	Ultra-rare
DHRS12	13	13-52348118-A-C	rs151240287	Ultra-rare
ATP7B	13	13-52513300-C-G	13-52513300-C-G	Ultra-rare
ATP7B	13	13-52515330-A-G	rs60431989	Ultra-rare
ATP7B	13	13-52518281-G-T	rs76151636	Ultra-rare
ATP7B	13	13-52523886-G-C	rs185149827	Ultra-rare
ATP7B	13	13-52524504-G-A	rs539585071	Ultra-rare
ATP7B	13	13-52548844-A-G	rs936120525	Ultra-rare
ABCC4	13	13-95815456-CT-C	13-95815456-CT-C	Ultra-rare
ABCC4	13	13-95840727-C-T	rs368017938	Ultra-rare
ABCC4	13	13-95863008-C-A	rs11568658	Ultra-rare
SLC15A1	13	13-99339860-G-A	rs140606428	Ultra-rare
SLC15A1	13	13-99356589-C-A	rs554040423	Ultra-rare
SLC15A1	13	13-99361007-A-G	rs112181280	Ultra-rare
SLC15A1	13	13-99364811-G-A	rs578219578	Ultra-rare
SLC15A1	13	13-99374056-DEL7-A	rs1310096083	Ultra-rare
SLC7A7	14	14-23243709-T-TA	rs1254380334	Ultra-rare
SLC7A7	14	14-23248089-A-G	rs368416948	Ultra-rare
SLC7A7	14	14-23249200-A-C	14-23249200-A-C	Ultra-rare
SLC7A8	14	14-23596522-C-T	rs140596505	Ultra-rare
SLC7A8	14	14-23652062-G-T	rs369866663	Ultra-rare
SLC22A17	14	14-23818508-C-T	rs369249944	Ultra-rare
DHRS2	14	14-24108198-C-T	rs2295908	Ultra-rare
DHRS2	14	14-24108407-C-T	rs199596675	Ultra-rare
DHRS2	14	14-24113687-G-A	rs751752790	Ultra-rare
DHRS4	14	14-24424306-G-A	rs79086208	Ultra-rare
DHRS4	14	14-24435582-A-G	rs573605554	Ultra-rare
DHRS4L2	14	14-24458283-G-T	rs113351006	Common
DHRS4L2	14	14-24459396-G-A	rs145720846	Ultra-rare
DHRS4L2	14	14-24459407-G-A	rs61732730	Common

DHRS4L2	14	14-24459431-G-A	rs754318476	Ultra-rare
DHRS4L2	14	14-24464257-G-A	rs148508271	Common
DHRS4L2	14	14-24464282-T-A	rs113886442	Ultra-rare
DHRS4L2	14	14-24464325-G-A	rs149046158	Ultra-rare
DHRS4L2	14	14-24470138-C-T	rs1811890	Rare
DHRS4L2	14	14-24470294-G-C	rs745501085	Ultra-rare
DHRS4L2	14	14-24470294-G-A	14-24470294-G-C	Ultra-rare
DHRS4L2	14	14-24470690-T-TA	rs368539076	Rare
DHRS1	14	14-24760788-C-G	rs187365006	Ultra-rare
DHRS7	14	14-60619736-A-G	rs773531485	Ultra-rare
DHRS7	14	14-60619877-T-C	rs147318951	Ultra-rare
GPX2	14	14-65406402-G-A	rs17881652	Ultra-rare
SLC10A1	14	14-70243094-TTTTTG-T	rs746771881	Ultra-rare
SLC10A1	14	14-70245157-A-G	rs72547507	Ultra-rare
SLC10A1	14	14-70245220-G-C	rs541801766	Ultra-rare
ALDH6A1	14	14-74534247-A-G	14-74534247-A-G	Ultra-rare
ALDH6A1	14	14-74539000-G-A	rs560772628	Ultra-rare
ALDH6A1	14	14-74551097-T-C	rs74062580	Ultra-rare
SLC28A2	15	15-45557324-T-C	rs781745682	Ultra-rare
SLC28A2	15	15-45562415-C-T	rs149533268	Ultra-rare
CYP19A1	15	15-51514707-C-T	rs142652579	Ultra-rare
CYP19A1	15	15-51520065-C-A	15-51520065-C-A	Ultra-rare
CYP11A1	15	15-74632055-G-A	rs560966443	Ultra-rare
CYP1A1	15	15-75012838-G-A	rs148638069	Ultra-rare
CYP1A1	15	15-75012979-G-A	rs41279188	Ultra-rare
CYP1A1	15	15-75013106-C-A	rs578124762	Ultra-rare
CYP1A1	15	15-75013576-C-T	rs201174966	Ultra-rare
CYP1A1	15	15-75013621-C-A	rs200723875	Ultra-rare
CYP1A1	15	15-75013845-C-T	rs1382709172	Ultra-rare
CYP1A1	15	15-75014049-G-A	rs34260157	Ultra-rare
CYP1A1	15	15-75015036-G-A	rs45442501	Ultra-rare
CYP1A1	15	15-75015206-A-G	rs17861094	Common
CYP1A1	15	15-75015210-G-A	rs371190271	Ultra-rare
CYP1A1	15	15-75015367-C-T	rs145924908	Ultra-rare
CYP1A1	15	15-75017781-C-T	rs569966459	Ultra-rare
CYP1A2	15	15-75042233-G-A	rs376605220	Ultra-rare
CYP1A2	15	15-75042261-C-T	rs761818825	Ultra-rare
CYP1A2	15	15-75042296-G-A	rs45565238	Ultra-rare
CYP1A2	15	15-75042454-C-A	rs141543251	Ultra-rare
CYP1A2	15	15-75042692-T-G	rs45540640	Ultra-rare
CYP1A2	15	15-75042776-G-A	rs201537008	Ultra-rare
CYP1A2	15	15-75042848-A-T	rs561167723	Ultra-rare
CYP1A2	15	15-75047173-T-C	rs201485133	Ultra-rare
CYP1A2	15	15-75047363-C-A	rs143193369	Ultra-rare
SLC28A1	15	15-85430019-G-A	rs114615130	Common
SLC28A1	15	15-85431015-AAG-A	rs1180091851	Ultra-rare
SLC28A1	15	15-85451954-C-G	rs754483260	Ultra-rare

SLC28A1	15	15-85452026-G-T	rs529925221	Ultra-rare
SLC28A1	15	15-85467235-T-C	rs116218523	Ultra-rare
SLC28A1	15	15-85476443-TG-T	rs547592684	Rare
SLC28A1	15	15-85486730-T-C	rs45584739	Rare
SLC28A1	15	15-85487815-G-A	rs149246522	Ultra-rare
SLC28A1	15	15-85488079-T-G	rs200930274	Ultra-rare
SLCO3A1	15	15-92459413-C-T	15-92459413-C-T	Ultra-rare
SLCO3A1	15	15-92459620-C-T	15-92459620-C-T	Ultra-rare
SLCO3A1	15	15-92690320-T-INS3	rs752157340	Ultra-rare
HAGH	16	16-1869957-C-T	rs578067137	Ultra-rare
HAGH	16	16-1876776-G-A	rs568657526	Ultra-rare
ABCC1	16	16-16101812-G-A	rs945037085	Ultra-rare
ABCC1	16	16-16103682-C-T	rs8187844	Ultra-rare
ABCC1	16	16-16142137-C-T	rs532663494	Ultra-rare
ABCC1	16	16-16184314-T-C	rs368349372	Ultra-rare
ABCC1	16	16-16208715-C-T	rs201602318	Ultra-rare
ABCC1	16	16-16218659-C-T	rs769212105	Ultra-rare
ABCC1	16	16-16230363-G-A	rs183032276	Ultra-rare
ABCC6	16	16-16244000-C-T	rs1362558112	Ultra-rare
ABCC6	16	16-16248831-G-A	rs63750759	Ultra-rare
ABCC6	16	16-16251579-G-A	rs72653749	Ultra-rare
ABCC6	16	16-16253422-A-T	rs114017587	Ultra-rare
ABCC6	16	16-16257015-C-T	rs63750427	Ultra-rare
ABCC6	16	16-16272734-G-A	rs549658225	Ultra-rare
ABCC6	16	16-16272776-C-T	rs67561842	Ultra-rare
ABCC6	16	16-16284091-A-G	16-16284091-A-G	Ultra-rare
SULT1A2	16	16-28604599-A-G	rs138180373	Ultra-rare
SULT1A2	16	16-28604815-G-GT	rs557693940	Rare
SULT1A2	16	16-28606753-G-C	rs559346977	Ultra-rare
SULT1A2	16	16-28606963-A-G	rs546104628	Ultra-rare
SULT1A2	16	16-28607196-G-A	rs10797300	Rare
SULT1A2	16	16-28607257-T-C	rs371864637	Ultra-rare
SULT1A1	16	16-28617218-G-A	rs767993186	Ultra-rare
SULT1A1	16	16-28618278-C-T	rs141878102	Ultra-rare
SULT1A1	16	16-28619841-G-A	rs201320226	Ultra-rare
SULT1A3	16	16-30212594-CTG-C	16-30212594-CTG-C	Ultra-rare
ABCC12	16	16-48117819-C-G	rs1343529336	Ultra-rare
ABCC12	16	16-48120732-T-C	rs181148639	Ultra-rare
ABCC12	16	16-48121987-G-A	rs144720520	Rare
ABCC12	16	16-48130781-C-T	rs36102575	Ultra-rare
ABCC12	16	16-48138182-G-A	rs776755685	Ultra-rare
ABCC12	16	16-48138264-T-C	rs150804137	Ultra-rare
ABCC12	16	16-48145704-G-A	rs146815895	Ultra-rare
ABCC12	16	16-48149528-A-G	rs767824146	Ultra-rare
ABCC12	16	16-48151206-T-C	rs199628644	Ultra-rare
ABCC12	16	16-48172226-G-A	rs142926867	Ultra-rare
ABCC12	16	16-48173109-C-T	rs113496237	Ultra-rare

ABCC12	16	16-48173109-C-G	16-48173109-C-G	Ultra-rare
ABCC12	16	16-48173126-C-A	rs61741201	Rare
ABCC12	16	16-48173207-T-TCAAA	16-48173207-T-TCAAA	Ultra-rare
ABCC12	16	16-48174812-AT-A	rs769854900	Ultra-rare
ABCC12	16	16-48177897-CTT-C	rs148156488	Common
ABCC12	16	16-48177911-G-A	rs140339167	Ultra-rare
ABCC12	16	16-48180235-G-A	16-48180235-G-A	Ultra-rare
ABCC11	16	16-48204084-C-T	rs148715549	Ultra-rare
ABCC11	16	16-48204130-C-T	rs60681475	Ultra-rare
ABCC11	16	16-48209178-C-T	rs141466191	Rare
ABCC11	16	16-48209181-G-C	rs111697082	Ultra-rare
ABCC11	16	16-48209223-C-T	rs572621805	Ultra-rare
ABCC11	16	16-48210867-T-C	16-48210867-T-C	Ultra-rare
ABCC11	16	16-48221293-G-A	rs139927866	Ultra-rare
ABCC11	16	16-48234381-G-A	rs41282045	Ultra-rare
ABCC11	16	16-48237138-C-T	rs140932063	Ultra-rare
ABCC11	16	16-48249137-C-T	rs534254215	Ultra-rare
ABCC11	16	16-48250144-C-A	rs146029165	Ultra-rare
ABCC11	16	16-48256649-C-T	rs577177114	Ultra-rare
ABCC11	16	16-48258198-C-T	rs17822931	Ultra-rare
ABCC11	16	16-48258306-G-A	rs543285690	Ultra-rare
CES1	16	16-55853446-C-A	rs4513095	Ultra-rare
CES1	16	16-55855402-C-A	rs150241462	Ultra-rare
CES1	16	16-55857486-C-A	rs369300055	Ultra-rare
CES1	16	16-55857577-C-T	rs778421449	Ultra-rare
CES1	16	16-55862746-T-C	rs756584724	Ultra-rare
CES1	16	16-55862769-G-A	rs3177828	Ultra-rare
CES1	16	16-55862817-A-C	rs151291296	Ultra-rare
CES1	16	16-55866915-C-T	rs139063675	Rare
CES2	16	16-66972077-C-T	rs28382815	Ultra-rare
CES2	16	16-66972089-C-A	rs1310482417	Ultra-rare
CES2	16	16-66972128-A-G	rs142873931	Ultra-rare
CES2	16	16-66973260-C-T	rs201254381	Ultra-rare
CES2	16	16-66976570-C-A	rs149858755	Ultra-rare
CHST4	16	16-71570593-A-T	rs200919282	Ultra-rare
CHST4	16	16-71570772-G-T	rs535693595	Ultra-rare
CHST4	16	16-71570811-C-A	rs145974764	Rare
CHST4	16	16-71570827-A-T	rs201326146	Ultra-rare
CHST4	16	16-71570941-T-C	rs560301542	Ultra-rare
CHST4	16	16-71571421-C-G	rs188876913	Ultra-rare
CHST4	16	16-71571487-C-T	rs751240118	Ultra-rare
CHST5	16	16-75563048-C-G	rs572716805	Ultra-rare
CHST5	16	16-75563226-G-A	rs548379850	Ultra-rare
DPEP1	16	16-89687185-G-C	rs909748188	Ultra-rare
DPEP1	16	16-89702329-C-T	rs565633365	Ultra-rare
DPEP1	16	16-89702751-A-T	rs569391051	Ultra-rare
DPEP1	16	16-89702768-T-A	rs146843391	Ultra-rare

DPEP1	16	16-89702771-C-G	rs199613842	Ultra-rare
DPEP1	16	16-89703006-A-T	rs200211325	Ultra-rare
DPEP1	16	16-89703085-C-T	rs149167696	Ultra-rare
SLC2A4	17	17-7187632-C-G	17-7187632-C-G	Ultra-rare
SLC2A4	17	17-7187929-C-T	rs140743598	Ultra-rare
SLC2A4	17	17-7187930-G-A	rs150359014	Ultra-rare
SLC2A4	17	17-7189148-G-A	rs144216820	Ultra-rare
DHRS7C	17	17-9680599-A-T	rs200311450	Ultra-rare
DHRS7C	17	17-9683268-C-T	rs151324045	Ultra-rare
DHRS7C	17	17-9684824-A-C	17-9684824-A-C	Ultra-rare
ALDH3A2	17	17-19554905-G-A	17-19554905-G-A	Ultra-rare
ALDH3A2	17	17-19559733-C-T	17-19559733-C-T	Ultra-rare
ALDH3A1	17	17-19642928-C-T	rs544273331	Ultra-rare
ALDH3A1	17	17-19645447-T-C	17-19645447-T-C	Ultra-rare
ALDH3A1	17	17-19645452-A-G	rs149784212	Ultra-rare
ALDH3A1	17	17-19645458-T-C	rs145697414	Ultra-rare
ALDH3A1	17	17-19645922-G-A	rs146746671	Ultra-rare
DHRS7B	17	17-21075488-G-A	rs759070460	Ultra-rare
SLC13A2	17	17-26817373-C-T	rs569846341	Ultra-rare
DHRS13	17	17-27225548-GT-G	rs866243068	Ultra-rare
PNMT	17	17-37826569-C-T	rs150643373	Ultra-rare
ABCC3	17	17-48734516-G-A	rs140992360	Ultra-rare
ABCC3	17	17-48736703-G-C	rs1289908544	Ultra-rare
ABCC3	17	17-48741357-A-G	rs34346931	Ultra-rare
ABCC3	17	17-48741390-G-A	rs142924921	Rare
ABCC3	17	17-48742565-C-T	rs752594239	Ultra-rare
ABCC3	17	17-48746781-CG-C	rs781648694	Ultra-rare
ABCC3	17	17-48750362-C-T	rs376756717	Ultra-rare
ABCC3	17	17-48750500-G-A	rs577246238	Ultra-rare
ABCC3	17	17-48761326-G-A	rs141856639	Ultra-rare
ABCC3	17	17-48764949-C-T	rs547523159	Ultra-rare
MPO	17	17-56349029-G-A	rs145497027	Ultra-rare
MPO	17	17-56356523-G-INS1	17-56356523-G-INS1	Ultra-rare
MPO	17	17-56357221-G-A	rs181494077	Ultra-rare
CHST9	18	18-24496735-TG-T	18-24496735-TG-T	Ultra-rare
CHST9	18	18-24497273-C-CT	rs532223263	Ultra-rare
GPX4	19	19-1105450-C-T	rs370419921	Ultra-rare
CYP4F3	19	19-15756667-DEL2-G	rs761222710	Ultra-rare
CYP4F3	19	19-15757916-G-A	rs556714796	Ultra-rare
CYP4F3	19	19-15758022-T-A	rs369978218	Ultra-rare
CYP4F3	19	19-15758054-C-T	rs143175634	Ultra-rare
CYP4F3	19	19-15758096-T-C	rs1359889175	Ultra-rare
CYP4F3	19	19-15760722-G-A	rs551654869	Ultra-rare
CYP4F3	19	19-15760873-C-A	rs145216906	Ultra-rare
CYP4F3	19	19-15760973-G-T	rs753149678	Ultra-rare
CYP4F3	19	19-15760986-T-C	19-15760986-T-C	Ultra-rare
CYP4F3	19	19-15763446-G-T	rs376212056	Ultra-rare

CYP4F3	19	19-15770028-A-T	rs557594223	Ultra-rare
CYP4F3	19	19-15770183-GC-G	rs371410792	Rare
CYP4F12	19	19-15783946-G-A	rs7252926	Rare
CYP4F12	19	19-15791199-TAGG-T	19-15791199-TAGG-CAGG	Ultra-rare
CYP4F12	19	19-15791247-G-A	rs377107512	Ultra-rare
CYP4F12	19	19-15793205-T-A	rs554464390	Ultra-rare
CYP4F12	19	19-15794302-G-A	rs762064960	Ultra-rare
CYP4F12	19	19-15794455-C-G	19-15794455-C-G	Ultra-rare
CYP4F12	19	19-15795693-G-A	rs111563606	Rare
CYP4F12	19	19-15795890-C-T	rs199760551	Ultra-rare
CYP4F12	19	19-15795926-C-T	rs1200059809	Ultra-rare
CYP4F12	19	19-15806864-C-T	rs376201019	Ultra-rare
CYP4F12	19	19-15807036-G-A	rs552981017	Ultra-rare
CYP4F12	19	19-15807255-C-T	rs373149297	Ultra-rare
CYP4F12	19	19-15807722-T-C	rs1172762272	Ultra-rare
CYP4F12	19	19-15807724-C-INS9	rs1419100465	Ultra-rare
CYP4F12	19	19-15807728-G-T	rs10421387	Rare
CYP4F2	19	19-15989594-T-C	rs571754062	Ultra-rare
CYP4F2	19	19-15990239-C-A	rs201015006	Ultra-rare
CYP4F2	19	19-15990625-G-A	rs75222722	Ultra-rare
CYP4F11	19	19-16035683-G-A	rs572505030	Ultra-rare
CYP4F11	19	19-16038104-C-T	rs142657300	Ultra-rare
SLC27A1	19	19-17599816-G-A	rs150085935	Ultra-rare
SLC27A1	19	19-17612106-C-T	rs757218318	Ultra-rare
SLC27A1	19	19-17612165-A-T	19-17612165-A-T	Ultra-rare
SLC27A1	19	19-17612186-C-T	rs368454326	Ultra-rare
CYP2A6	19	19-41349786-G-T	rs145014075	Rare
CYP2A6	19	19-41351363-T-A	rs61605570	Ultra-rare
CYP2A6	19	19-41351908-G-A	rs528089983	Ultra-rare
CYP2A6	19	19-41354168-TG-T	rs561452609	Ultra-rare
CYP2A6	19	19-41354189-CTT-C	rs568811809	Ultra-rare
CYP2A6	19	19-41356225-C-A	rs558145012	Ultra-rare
CYP2A7	19	19-41381683-G-T	rs58682606	Common
CYP2A7	19	19-41383185-G-C	19-41383185-G-C	Ultra-rare
CYP2A7	19	19-41383220-G-A	rs142068831	Ultra-rare
CYP2A7	19	19-41383816-G-A	rs373592664	Ultra-rare
CYP2A7	19	19-41386516-C-G	rs376282662	Ultra-rare
CYP2A7	19	19-41387590-C-T	19-41387590-C-T	Ultra-rare
CYP2B6	19	19-41497292-G-GGC	rs781463375	Rare
CYP2B6	19	19-41497294-CCG-C	rs750679089	Rare
CYP2B6	19	19-41510026-C-T	rs183427203	Ultra-rare
CYP2B6	19	19-41510063-G-T	rs186335453	Ultra-rare
CYP2B6	19	19-41510243-C-T	rs370958436	Ultra-rare
CYP2B6	19	19-41515993-C-G	rs34698757	Ultra-rare
CYP2B6	19	19-41518244-C-T	rs34826503	Ultra-rare
CYP2B6	19	19-41518370-C-T	rs34097093	Ultra-rare
CYP2A13	19	19-41595975-C-G	19-41595975-C-G	Ultra-rare

CYP2A13	19	19-41595994-G-C	rs138225940	Ultra-rare
CYP2A13	19	19-41596016-G-C	rs372587042	Ultra-rare
CYP2A13	19	19-41597775-C-T	rs143140637	Ultra-rare
CYP2A13	19	19-41600191-C-T	rs138941528	Ultra-rare
CYP2A13	19	19-41600237-C-CA	rs762929559	Ultra-rare
CYP2A13	19	19-41601841-C-T	rs138870349	Ultra-rare
CYP2F1	19	19-41622107-G-GC	rs3833221	Very-Common
CYP2F1	19	19-41626270-G-A	rs199931801	Ultra-rare
CYP2F1	19	19-41626293-C-T	19-41626293-C-T	Ultra-rare
CYP2F1	19	19-41628821-C-T	rs369146753	Ultra-rare
CYP2F1	19	19-41633827-A-G	rs146190866	Ultra-rare
CYP2S1	19	19-41707230-C-T	rs145747863	Ultra-rare
SULT2B1	19	19-49096012-G-A	rs576779662	Ultra-rare
SULT2B1	19	19-49102445-G-C	rs201648343	Ultra-rare
HSD17B14	19	19-49335965-G-A	rs139341223	Rare
HSD17B14	19	19-49339082-A-T	rs141772243	Ultra-rare
HSD17B14	19	19-49339626-T-G	rs138723113	Ultra-rare
GSS	20	20-33519776-C-T	rs546916452	Ultra-rare
GSS	20	20-33519777-G-A	rs568602749	Ultra-rare
GSS	20	20-33519827-C-A	rs199916857	Ultra-rare
GSS	20	20-33529528-T-C	rs138659144	Ultra-rare
GSS	20	20-33533858-G-A	rs112712286	Ultra-rare
HNF4A	20	20-43043238-T-A	rs201086670	Ultra-rare
SLC13A3	20	20-45192192-T-C	20-45192192-T-C	Ultra-rare
SLC13A3	20	20-45194926-G-A	rs576353313	Ultra-rare
SLC13A3	20	20-45242291-G-A	rs200598488	Ultra-rare
SLC13A3	20	20-45242315-G-A	rs113419510	Ultra-rare
SLC13A3	20	20-45242355-A-C	rs144600005	Ultra-rare
SLC13A3	20	20-45242358-G-A	rs77731467	Common
CYP24A1	20	20-52786167-C-G	rs114579367	Ultra-rare
CYP24A1	20	20-52789538-C-A	rs114476330	Ultra-rare
SLCO4A1	20	20-61288378-A-G	rs144344332	Ultra-rare
SLCO4A1	20	20-61291813-G-A	rs570535583	Ultra-rare
CBR1	21	21-37442480-C-T	rs146570341	Ultra-rare
CBR3	21	21-37510186-C-T	rs144087608	Ultra-rare
ABCG1	21	21-43702382-A-G	21-43702382-A-G	Ultra-rare
ABCG1	21	21-43708163-C-T	rs577566323	Ultra-rare
SLC19A1	21	21-46957687-G-A	rs1418489942	Ultra-rare
GSTT2	22	22-24323227-G-A	rs5996646	Common
GSTT1	22	22-24376584-G-A	rs144686326	Ultra-rare
CYP2D6	22	22-42523483-C-T	rs61731586	Ultra-rare
CYP2D6	22	22-42523528-C-T	rs1058172	Ultra-rare
CYP2D6	22	22-42523582-A-G	rs1450231864	Ultra-rare
CYP2D6	22	22-42523592-G-A	rs72549347	Ultra-rare
CYP2D6	22	22-42523604-C-G	rs566108360	Ultra-rare
CYP2D6	22	22-42523855-G-A	rs140513104	Ultra-rare
CYP2D6	22	22-42524202-C-CA	rs767565288	Ultra-rare

CYP2D6	22	22-42524947-C-T	rs3892097	Rare
CYP2D6	22	22-42526640-G-A	rs536109057	Ultra-rare
CYP2D6	22	22-42526694-G-A	rs1065852	Common
CYB5R3	22	22-43019891-C-T	rs61745147	Ultra-rare
PPARA	22	22-46594460-T-TCCTG	22-46594460-T-TCCTG	Ultra-rare
PPARA	22	22-46611204-G-A	22-46611204-G-A	Ultra-rare
DHRSX	23	X-2161167-G-A	X-2161167-G-A	Ultra-rare
DHRSX	23	X-2161224-A-G	X-2161224-A-G	Ultra-rare
DHRSX	23	X-2161243-C-G	X-2161243-C-G	Ultra-rare
DHRSX	23	X-2343295-C-T	X-2343295-C-T	Ultra-rare
ABCB7	23	X-74289163-C-T	rs151288786	Ultra-rare
ATP7A	23	X-77244998-A-T	rs150526992	Ultra-rare
ATP7A	23	X-77254142-T-C	rs782589583	Ultra-rare
ATP7A	23	X-77264714-A-G	rs61742278	Rare

The longer insertions/deletions referred to below are

INS1	GGAGTCAGCTGATCAGTGGGGAAGCGCACGATCTCGTTGGAGACC
DEL2	GCTCCAGGTAGACACTGCACTGGCCA
INS3	TAATGGAATGCCACAGAATGTGTAATTAGG
DEL4	GTCCCACAGCAACAATA
DEL5	CGGGCGCGAGGTGAG
DEL6	CTGGTAAGT
DEL7	ACCTGGC
DEL8	GAGTAATT
INS9	CATCGGGCA

References

[1] Choudhury A, Aron S, Botigué L, Sengupta D, Botha G, Bensellak T, Wells G, Kumuthini J, Shriner D, Fakim YJ, Ghoorah AW, Dareng E, Odia T, Falola O, Adebiyi E, Hazelhurst S, Mazandu G, Nyangiri OA, Mbiyavanga M, Benkahla A, Kassim SK, Mulder N, Adebamowo SN, Chimusa ER, Gibbs RA, Muzny D, Metcalf G, TrypanoGEN Research Group, Rotimi C, Ramsay M, Adeyemo A, Lombard Z, Hanchard NA as members of the H3Africa Consortium. High Depth African Genomes Inform Human Migration and Health. In Preparation

[2] Alexander DH, Novembre J, Lange K. Fast model-based estimation of ancestry in unrelated individuals. Genome Research. 2009;19(9):1655–64. doi:10.1101/gr.094052.109.

[3] Jakobsson M, Rosenberg NA. CLUMPP: a cluster matching and permutation program for dealing with label switching and multimodality in analysis of population structure. Bioinformatics. 2007;23(14):1801–1806.

[4] Rentzsch P, Witten D, Cooper GM, Shendure J, Kircher M. CADD: Predicting the deleteriousness of variants throughout the human genome. Nucleic Acids Research. 2019;47(D1):D886–D894. doi:10.1093/nar/gky1016.

[5] Bhatia G, Patterson N, Sankararaman S, Price AL. Estimating and interpreting FST: The impact of rare variants. Genome Research. 2013;23(9):1514–1521. doi:10.1101/gr.154831.113.23.

[6] Ceballos FC, Joshi PK, Clark DW, Ramsay M. Runs of homozygosity : windows into population history and trait architecture. Nature Publishing Group. 2018;19(4):220–234. doi:10.1038/nrg.2017.109.

[7] Szpiech ZA, Mak ACY, White MJ, Hu D, Eng C, Burchard EG, et al. Ancestry-Dependent Enrichment of Deleterious Homozygotes in Runs of Homozygosity. The American Journal of Human Genetics. 2019;105(4):747–762. doi:10.1016/j.ajhg.2019.08.011.

[8] Ceballos FC, Hazelhurst S, Ramsay M. Runs of homozygosity in sub-Saharan African populations provide insights into complex demographic histories. Hum Genet. 2019;138(10):1123–1142.

[9] Chang CC, Chow CC, Tellier LC, Vattikuti S, Purcell SM, Lee JJ. Second-generation PLINK: rising to the challenge of larger and richer datasets. GigaScience. 2015;4(1):1–16. doi:10.1186/s13742-015-0047-8.
